# The Pteridaceae family diversity in Togo

**DOI:** 10.3897/BDJ.3.e5078

**Published:** 2015-07-15

**Authors:** Komla Elikplim Abotsi, Aboudou R. Radji, Germinal Rouhan, Jean-Yves Dubuisson, Kouami Kokou

**Affiliations:** ‡ Université de Lomé, Lomé, Togo; § Museum National d'Histoire Naturelle, Paris cedex 05, France

**Keywords:** Pteridaceae, diversity, revision, identification key, Togo

## Abstract

**Background:**

The Pteridaceae family is the largest fern family in Togo by its specific and generic diversity. Like all other families of ferns in the country, Pteridaceae are poorly studied and has no identification key. The objective of this study is to perform a taxonomic revision and list establishment of this family of leptosporangiate ferns in the light of current available knowledge about the family. Pteridaceae was also assessed in terms of its diversity and conservation status, this was conducted through the recent field data and the existing herbaria specimens. The current study permits to confirm the presence of *Pteris similis Kuhn*. which brought the number of Pteridaceae to 17 in Togo.

**New information:**

This study provides first local scientific information about the fern flora of Togo. It confirmed the presence of *Pteris similis* Kuhn. in Togo and brought the Pteridaceae family diversity to 17 species. A species identification key is provided for the easy identification of the Pteridaceae of Togo.

## Introduction

Ferns have been studied, in the past century, mainly using morphological traits whereas more light have been brought to the group with the recent development of cytology and molecular phylogeny (Pryer et al. 2001, Pryer et al. 2004, Smith et al. 2006, Smith et al. 2008, Christenhusz et al. 2011, Schuettpelz and Pryer 2007).

As a large family of leptosporangiate ferns (about 10% of the Monilophytes), Pteridaceae undergone several taxonomic and nomenclatural updates (Gastony and Rollo 1995, Gastony and Rollo 1998, Sanchez-Baracaldo 2004, Zhang et al. 2005, Kirkpatrick 2007, Prado et al. 2007, Schuettpelz et al. 2007, Windham et al. 2009, Beck et al. 2010, Hennequin et al. 2010, Schneider et al. 2013). The current family diversity is about 1000 species distributed in 53 genera (Schuettpelz et al. 2007, Christenhusz et al. 2011).

The Pteridaceae occupies aquatic and terrestrial environments. They are usually terrestrial, but sometimes aquatic, epilithic and epiphytic. However they abound in the humid tropics, the family includes several cosmopolitan species. They have a long or short rhizome, creeping, ascending, sub-erected or erected, and have splinters (more rarely hairs). They have a monomorphic limb, hemi- or rarely dimorphic, simple, pinnate, pedaled, sometimes decurrent; free and simple veins, or sometimes anastomosed, forming a network. The Sporangia are grouped in marginal or intra-marginal sori, without true indusium, often protected by the reflected margin of the lamina, or arranged along the vein. Each sporangia has a vertical and interrupted annulus, with a little or no visible receptacle.

The family is well present in Togo but few studies hitherto focused on it. The current data of the country's biodiversity (MERF 2009) shows a diversity represented almost in its entirety within the ecological zone 4 of Togo (the guineean forest zone of the country, according to Ern (1979) ecological classification of Togo) for all Monilophytes.

Recent revisions of the family are not taken into account neither at the herbarium of the University of Lomé nor in the flora of Togo. Many names have then been placed in synonymy in recent databases (The Plant List 2015) while others gender and/or family have been changed.

The current study aims at updating the Pteridaceae list and taxonomy in Togo. An identification key was created to facilitate the identification of the identified species.

## Materials and methods

### Study area

The study area is Togo. It is located in the Gulf of Guinea, and surface covers approximately 56 785 km^2^. Its forest surface is counts among the least in the West African sub-region.

**Figure 1.**
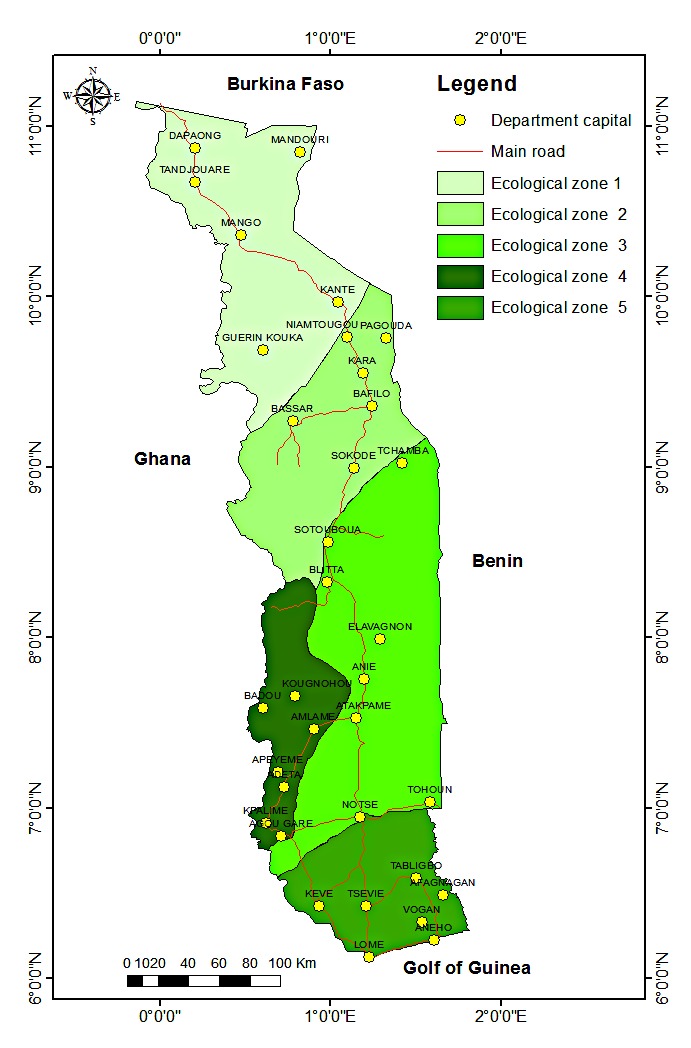
Togo ecological zones. Ecological Zone 1: Refers to the Northern Plains. It enjoys a Sudanese climate characterized by an average of 6 to 7 months of drought. Rainfall varies from 800 to 1000 mm and water per year. The temperatures are between 17 and 39° C. The predominant vegetation is the Sudan savanna with few islands of dry forests and gallery forests. The soil is tropical ferruginous, sandy.Ecological Zone 2: It is covered with a mosaic of dry forests of mountain and forest galleries. The climate is Sudano-Guinean. The rainy season extends from April to October and the dry season is characterized by the presence of the Harmattan, a dry and cold wind. Rainfall is irregular and reaches 1200-1300 mm of water per year. The soils are thin and contain a high proportion of coarse elements. Ferruginous tropical soils are also present.Ecological Zone 3: It corresponds to the Guinean savannas of central area plains enjoying a tropical climate with a rainy season extends from May to October. Rainfall varies between 1200 and 1500 mm per year. The temperature is between 25 and 40° C. The soils are mainly ferruginous. Semi-deciduous forests are found in the southern part and dry forests in the northern part.Ecological Zone 4: This corresponds to the southern part of the Mount "Togo" and has a sub-equatorial climate of transition, characterized by a long rainy season from March to October with a small interruption in August or September. Rainfall varies between 1300 and 1600 mm per year. The vegetation is constitute of ​​rainforests, on deep red lateritic soils.Ecological Zone 5: It is the South of the coastal plains area, with a sub-equatorial climate, characterized by a lack of rainfall in it's southern part. Rainfall varies from 800 mm at the coast up to 1200 mm per year at the northern limit of the zone. The vegetation is composed by a mosaic of savannah, farmland and dry forests (Kokou and Caballé 2000).

Three main types of climate are found in Togo. In the North, there is the tropical climate with variations increasingly wet, going to the South. The southern coastal area has a sub-equatorial climate. A transitional climate between these two types is found in the mountains of the south. Togo has varied soils. There are five main soil classes (Levêque 1979) which are: 1 / the tropical ferruginous soils, leached, indurated or waterlogged, 2 / slightly evolved alluvial soils or erosion intake, 3 / vertisols rich in swelling clay and mineral elements, 4 / ferralitic soils characterized by the persistence of iron and aluminum and other cations leaching (these are the best soils in the country), 5 / hydromorphic gley soils, clayey, poorly drained, encountered the edge of river mouths and some depressions. The relief consists essentially of two large plains separated by a strip of mountains which takes the country in scarf from the Southwest to the Northeast. The plains are mostly covered with savanna and patches of dry forest. The "Togo" Mounts are covered by dry forest in their northern part and rain forest in the southern part. This last part constitute the Togolese portion of the West African's rainforest and is the ecological zone 4 (Ern 1979). Indeed, Togo is divided into five ecological zones characterized by their climate, vegetation and soil type (Fig. 1).

There have been new harvest in the ecologic zone 4 of Togo in order to enrich the plant materials available for this study.

### Materials

The study is based on new harvests from the ecological zone 4 (215 specimens) and specimens from herbaria of the University of Lomé (TOGO) (87 specimens) and the National Museum of Natural History in Paris (P) (122 specimens).

### Methodology

New harvests were conducted along the banks of the natural ecosystem's streams in the study area along a topographic gradient. As many others authors, Moran (1995) works on the ecology of African ferns have indeed demonstrated fern’s preference for mountainous rainforests and the banks of rivers.

Data from this new inventory is coupled with ecological and geographical data from previously performed harvests between 1973 and 1994 across the country and available in the RIHA data base (African Herbaria Computer Network) of the herbarium of Lomé.

Each harvest consists of one or more photos of the whole plant in its habitat, a herbarium specimen represented by the whole plant (or a fragment then comprising parts of all organs) and a piece of lamina in silica gel (Gaudeul and Rouhan 2013) for future genetic studies. For epiphytic taxa, a fertile sample of the porophyte is also collected. The identification of new specimens was then performed thanks to the flora of Cameroon (Tardieu-Blot 1964) and Southern Africa (Roux 2001). The specimens are then identified and associated with specimens already available in herbaria of Paris and Lomé. The observed classification of the taxonomy is the one established by Christenhusz et al. (2011). The list of synonyms used comes from the online database of “The Plant List” (The Plant List 2015).

The morphological observations of all available specimens (new specimens and old herbarium collections) enabled the establishment of a list of morphological descriptors which have been added to the ecological characteristics of each species (Table 1). These descriptors are integrated in a knowledge base through the Xper² software.

Xper² is a free software for management, editing, and analysis of taxonomic knowledge bases (Ung et al. 2010).

All species identified as a result of the study and the identification of all available specimens were then associated with the states of the descriptors that characterize them. This same approach was also used by Corvez and Grand (2014) in order to identify better descriptors for ferns's taxonomy and systematics. The software has permit to classify the descriptors according to their ability to discriminate the different species identified and incorporated into the knowledge base. This discriminating power is given by the XPER index, provided by the software itself. This index has the advantage of not being a simple index of similarity or dissimilarity unlike indices of Jaccard or Sokal & Michener also provided by the software. It allows thanks to its calculation method to highlight the descriptors whose states allow better identification of concerned taxa. A descriptor is then more efficient if it exists in at least two exclusive modalities within the studied species group. The relevance of the descriptors is then given by their discriminating power. The best descriptors are those which have the highest index. The index of XPER range from 0 to 1. It is calculated by the ratio between the number of couples with no common values and the total number of couples. A descriptor with little or no discriminating power can however become very decisive when the state of the descriptor which conditioned its applicability is verified. All first order descriptors (not conditioned by the effectivity of any other descriptor) which have no discriminating power are then eliminated. This easily allowed to identify similarities and differences between species on one hand, and on the other hand between genera.

The different states of retained descriptors were used to create a dichotomous identification key of the Togolese Pteridaceae family species. For this purpose, the specimens were segregated into two groups according to their similarities and dissimilarities. Within each group, two groups are newly created and so on until the species rank. The key nodes are defined by the dissimilarities while the branches are defined by the similarities between species.

Geographic data were collected from herbarium specimen labels and new harvests sheets in order to edit a distribution map of the Pteridaceae family in Togo.

## Checklists

### Checklist of Pteridaceae from Togo

#### Acrostichum aureum

L.

Acrostichum inaequale Willd, *A. guineense* Gaudich, *Chrysodium inaequale* (Willd.) Fée, *C. vulgare* Fée, *C. aureum* (L) Mett

##### Materials

- **Type status:** Other material. **Occurrence:** catalogNumber: 11958; recordNumber: 4877; recordedBy: J.-F. Brunel; lifeStage: Adult; reproductiveCondition: Fertile; **Taxon:** nameAccordingToID: The Plant List; scientificName: *Acrostichum aureum* L.; kingdom: Plantae; phylum: Monilophyta; class: Polypodiidae; order: Polypodiales; family: Pteridaceae; genus: Acrostichum; specificEpithet: aureum; taxonRank: species; scientificNameAuthorship: L.; **Location:** continent: Africa; country: Togo; stateProvince: Maritime; decimalLatitude: 6.2333333; decimalLongitude: 1.2227778; geodeticDatum: WGS 1984; **Identification:** identifiedBy: J.-F. Brunel; **Record Level:** institutionID: Herbarium togoense; collectionID: TOGO
- **Type status:** Other material. **Occurrence:** catalogNumber: 11960; recordNumber: 102; recordedBy: K. Akpagana; lifeStage: Adult; reproductiveCondition: Fertile; **Taxon:** nameAccordingToID: The Plant List; scientificName: Acrostichum aureum L.; kingdom: Plantae; phylum: Monilophyta; class: Polypodiidae; order: Polypodiales; family: Pteridaceae; genus: Acrostichum; specificEpithet: aureum; taxonRank: species; scientificNameAuthorship: L.; **Location:** continent: Africa; country: Togo; stateProvince: Maritime; decimalLatitude: 6.2333333; decimalLongitude: 1.2227778; geodeticDatum: WGS 1984; **Identification:** identifiedBy: J.-F. Brunel; dateIdentified: /5/1983; **Event:** eventDate: /5/1983; **Record Level:** institutionID: Herbarium togoense; collectionID: TOGO

##### Ecological interactions

###### Native status

Native

###### Conservation status

Least Concern

##### Distribution

Togo (Ecological Zone 5), USA, U.S. Virgin Isl., Costa Rica, Galapagos, Puerto Rico, Hispaniola, Jamaica, Cuba, Nicaragua, Barbados, Mexico, Belize, Guatemala, Honduras, Panama, Colombia, Venezuela, Ecuador, Peru, Brazil, Trinidad, Guadeloupe, Martinique, St. Lucia, Grenada, Barbados, Guyana, Surinam, French Guiana, Cayman Isl., Bahamas, Turks & Caicos Isl., Isla del Coco, Australia, South Africa, Zambia, Mozambique, SE-Zimbabwe, Kenya, Tanzania, Zanzibar, Pemba Isl., Principe Isl., Sao Tomé, Bioko Isl., Senegal, Guinea-Bissau, Guinea, Gambia, Sierra Leone, Liberia, Ivory Coast, Ghana, Nigeria, Cameroon, Equatorial Guinea, Gabon, Angola, Madagascar, Mauritius, La Réunion, Micronesia, Palau Isl., Fiji, Western Samoa, American Samoa, Marianas, Society Isl., Tonga, New Caledonia, Niue, Austral Isl., China, Taiwan, Ryukyu Isl., peninsular Malaysia, India, Andaman Isl., Nicobar Isl., Philippines, Sri Lanka, Vietnam, Thailand, Cambodia, Sulawesi

##### Notes

*Acrostichum aureum* is a fern with erect rhizome and fronds in clumps. Young fronds are entire while adults are pinnate, measuring 1 to over 2m in length. The petiole is 30-50 cm long, rigid and naked. The leaf blade is oblong-lanceolate, about 50 cm to 2 m long, with lanceolate pinnae. The pinnae are 20-30 cm long for 3-5 cm wide, alternate, spaced about 3 cm, with cuneate-oblique base. The upper pinnae are sessile while basal pinnae are stalked (Fig. 4a). Fertile pinnae are slightly narrower and completely covered by sporangia on their underside (Fig. 4b). This fern grows in the backward of mangroves, forming almost impenetrable bushes.

#### Actiniopteris radiata

(Sw.) Link.

Acrostichum dichotomum Forssk., *A. radiatum* (Sw.) Poir, *Acropteris radiata* (Sw.) Link, *Pteris radiata* (Sw.) Bojer, *Asplenium polydactylon* Webb., *A. radiatum* Sw, *Actiniopteris australis* Sim., A. australis var. radiata (Sw.) C. Chr

##### Materials

- **Type status:** Other material. **Occurrence:** catalogNumber: 11961; recordNumber: 5001; recordedBy: J.-F. Brunel; lifeStage: Adult; reproductiveCondition: Fertile; **Taxon:** nameAccordingToID: The Plant List; scientificName: *Actinopteris radiata* Koen ex Roxb.; kingdom: Plantae; phylum: Monilophyta; class: Polypodiidae; order: Polypodiales; family: Pteridaceae; genus: Actinopteris; specificEpithet: radiata; taxonRank: species; scientificNameAuthorship: Koehn ex Roxb.; **Location:** continent: Africa; country: Togo; stateProvince: Kara; decimalLatitude: 9.5833333; decimalLongitude: 1.1666667; geodeticDatum: WGS 1984; **Identification:** identifiedBy: J.-F. Brunel; dateIdentified: /6/1973; **Event:** eventDate: /6/1973; **Record Level:** institutionID: Herbarium togoense; collectionID: TOGO

##### Ecological interactions

###### Native status

Native

##### Distribution

Togo (Ecological Zone 2), South Africa, Namibia, Swaziland, Botswana, Zimbabwe, Mozambique, Zambia, D.R.Congo, Zimbabwe, Uganda, Kenya, Tanzania, Zanzibar, Djibouti, Mali, Nigeria, Cameroon, Somalia, Madagascar, Comores, Seychelles, La Réunion, Mauritius, Angola, Sudan, SE-Egypt, Ethiopia, N-Yemen, Saudi Arabia, India, Sri Lanka, Iran, Afghanistan, Pakistan, Nepal, Burma, Cape Verde Isl.

##### Notes

Small fern with an erect rhizome and fronds in clumps, with lanceolate scales toothed at their base, *Actiniopteris radiata* has a petiole of 5-15 cm long, sparsely scaly (Fig. 5a). The scales are marginate and reddish at the base. The lamina is flabellate, 2 to 4 cm long and 3-5 cm wide, semi-circular. It is divided into linear lobes, toothed at the top (Fig. 5b). The texture of the leaf is leathery. *Actiniopteris radiata* usually grows on rocks, cliff edges and rocky terrain, especially in dry forests.

#### Adiantum incisum

Forssk.

Adiantum capillus-gorgonis Webb, *A. caudatum* sensu Hook, A. caudatum var. hirsutum Kuhn, *A. zollingeri* sensu Carruth.

##### Materials

- **Type status:** Other material. **Occurrence:** catalogNumber: 11988; recordNumber: 100bis; recordedBy: J.-F. Brunel; lifeStage: Adult; reproductiveCondition: Fertile; **Taxon:** nameAccordingToID: The Plant List; scientificName: *Adiantum incisum* Forsk.; kingdom: Plantae; phylum: Monilophyta; class: Polypodiidae; order: Polypodiales; family: Pteridaceae; genus: Adiantum; specificEpithet: incisum; taxonRank: species; scientificNameAuthorship: Forsk.; **Location:** continent: Africa; country: Togo; stateProvince: Plateaux; decimalLatitude: 7; decimalLongitude: 0.75; geodeticDatum: WGS 1984; **Identification:** identifiedBy: C.A. Meyer; dateIdentified: /4/1980; **Event:** eventDate: 1973; **Record Level:** institutionID: Herbarium togoense; collectionID: TOGO
- **Type status:** Other material. **Occurrence:** catalogNumber: 30096; recordNumber: 306; recordedBy: ABOTSI, SODJINOU & MINGOU; lifeStage: Adult; reproductiveCondition: Fertile; **Taxon:** nameAccordingToID: The Plant List; scientificName: *Adiantum incisum* Forsk.; kingdom: Plantae; phylum: Monilophyta; class: Polypodiidae; order: Polypodiales; family: Pteridaceae; genus: Adiantum; specificEpithet: incisum; taxonRank: species; scientificNameAuthorship: Forsk.; **Location:** continent: Africa; country: Togo; stateProvince: Plateaux; decimalLatitude: 6.84667183; decimalLongitude: 0.74863042; geodeticDatum: WGS 1984; **Identification:** identifiedBy: K. E. Abotsi; dateIdentified: /05/2013; **Event:** eventDate: 04-16-13; **Record Level:** institutionID: Herbarium togoense; collectionID: TOGO
- **Type status:** Other material. **Occurrence:** catalogNumber: 30097; recordNumber: 307; recordedBy: ABOTSI, SODJINOU & MINGOU; lifeStage: Adult; reproductiveCondition: Fertile; **Taxon:** nameAccordingToID: The Plant List; scientificName: *Adiantum incisum* Forsk.; kingdom: Plantae; phylum: Monilophyta; class: Polypodiidae; order: Polypodiales; family: Pteridaceae; genus: Adiantum; specificEpithet: incisum; taxonRank: species; scientificNameAuthorship: Forsk.; **Location:** continent: Africa; country: Togo; stateProvince: Plateaux; decimalLatitude: 6.84667183; decimalLongitude: 0.74863042; geodeticDatum: WGS 1984; **Identification:** identifiedBy: K. E. Abotsi; dateIdentified: /05/2013; **Event:** eventDate: 04-16-13; **Record Level:** institutionID: Herbarium togoense; collectionID: TOGO
- **Type status:** Other material. **Occurrence:** catalogNumber: 30104; recordNumber: 340; recordedBy: ABOTSI, SODJINOU & MINGOU; lifeStage: Adult; reproductiveCondition: Fertile; **Taxon:** nameAccordingToID: The Plant List; scientificName: *Adiantum incisum* Forsk.; kingdom: Plantae; phylum: Monilophyta; class: Polypodiidae; order: Polypodiales; family: Pteridaceae; genus: Adiantum; specificEpithet: incisum; taxonRank: species; scientificNameAuthorship: Forsk.; **Location:** continent: Africa; country: Togo; stateProvince: Plateaux; decimalLatitude: 8.01889124; decimalLongitude: 0.62737159; geodeticDatum: WGS 1984; **Identification:** identifiedBy: K. E. Abotsi; dateIdentified: /05/2013; **Event:** eventDate: 05-11-13; **Record Level:** institutionID: Herbarium togoense; collectionID: TOGO
- **Type status:** Other material. **Occurrence:** catalogNumber: 30106; recordNumber: 348; recordedBy: ABOTSI, SODJINOU & MINGOU; lifeStage: Adult; reproductiveCondition: Fertile; **Taxon:** nameAccordingToID: The Plant List; scientificName: *Adiantum incisum* Forsk.; kingdom: Plantae; phylum: Monilophyta; class: Polypodiidae; order: Polypodiales; family: Pteridaceae; genus: Adiantum; specificEpithet: incisum; taxonRank: species; scientificNameAuthorship: Forsk.; **Location:** continent: Africa; country: Togo; stateProvince: Plateaux; decimalLatitude: 6.95154473; decimalLongitude: 0.59787902; geodeticDatum: WGS 1984; **Identification:** identifiedBy: K. E. Abotsi; dateIdentified: /05/2013; **Event:** eventDate: 05-21-13; **Record Level:** institutionID: Herbarium togoense; collectionID: TOGO
- **Type status:** Other material. **Occurrence:** catalogNumber: 30107; recordNumber: 351; recordedBy: ABOTSI, SODJINOU & MINGOU; lifeStage: Adult; reproductiveCondition: Fertile; **Taxon:** nameAccordingToID: The Plant List; scientificName: *Adiantum incisum* Forsk.; kingdom: Plantae; phylum: Monilophyta; class: Polypodiidae; order: Polypodiales; family: Pteridaceae; genus: Adiantum; specificEpithet: incisum; taxonRank: species; scientificNameAuthorship: Forsk.; **Location:** continent: Africa; country: Togo; stateProvince: Plateaux; decimalLatitude: 7.5118567; decimalLongitude: 0.60163398; geodeticDatum: WGS 1984; **Identification:** identifiedBy: K. E. Abotsi; dateIdentified: /05/2013; **Event:** eventDate: 04-03-14; **Record Level:** institutionID: Herbarium togoense; collectionID: TOGO
- **Type status:** Other material. **Occurrence:** catalogNumber: 30108; recordNumber: 352; recordedBy: ABOTSI, SODJINOU & MINGOU; lifeStage: Adult; reproductiveCondition: Fertile; **Taxon:** nameAccordingToID: The Plant List; scientificName: *Adiantum incisum* Forsk.; kingdom: Plantae; phylum: Monilophyta; class: Polypodiidae; order: Polypodiales; family: Pteridaceae; genus: Adiantum; specificEpithet: incisum; taxonRank: species; scientificNameAuthorship: Forsk.; **Location:** continent: Africa; country: Togo; stateProvince: Plateaux; decimalLatitude: 7.5118567; decimalLongitude: 0.60163398; geodeticDatum: WGS 1984; **Identification:** identifiedBy: K. E. Abotsi; dateIdentified: /05/2013; **Event:** eventDate: 04-03-14; **Record Level:** institutionID: Herbarium togoense; collectionID: TOGO

##### Ecological interactions

###### Native status

Native

##### Distribution

Togo (Ecological Zone 4), South Africa, Swaziland, Namibia, Zimbabwe, Zambia, Malawi, Mozambique, Ghana, Nigeria, Uganda, Kenya, Tanzania, Somalia, Madagascar, Ivory Coast, Angola, Ethiopia, N-Yemen, S-Yemen, Oman, Saudi Arabia, Pakistan, Galapagos, Jammu & Kashmir, India, Cape Verde Isl.

##### Notes

*Adiantum incisum* has a short and erect rhizome (Fig. 6a), with linear scales at the base, 4 to 5 mm long over 0.2 mm wide, blackish, with a narrow margin pale. The fronds, erect or drooping, are pinnate and are arranged in tufts. They are 10 to 40 cm long. The rachis and stem are dark brown. The lower half of the stem is densely hairy, and sometimes wears narrow pale brown scales. Pinnae, 0.7 to 2.3 cm long and 4-11 mm wide, become smaller and smaller at the top (Fig. 6b). They are alternate, oblong to obliquely triangular, slightly to deeply incised or lobed at the outer and lower margins, entire at the lower margin. The base is cuneiform or rarely rounded, generally, sparsely to densely covered with white or brown hairs (Fig. 6c). The sori are circular or oblong, 1 to 3.5 mm wide (Fig. 6d). *Adiantum incisum* usually grows on damp rocks and mountainsides in forest areas.

#### Adiantum lunulatum

Burm.

Adiantum philippense L., *A. lunulata* Burm., *A. lunatum* Cav., *A. arcuatum* Sw.

##### Materials

- **Type status:** Other material. **Occurrence:** catalogNumber: 12005; recordNumber: 8689; recordedBy: J.-F. Brunel; lifeStage: Adult; reproductiveCondition: Fertile; **Taxon:** nameAccordingToID: The Plant List; scientificName: *Adiantum lunulatum* Burm.; kingdom: Plantae; phylum: Monilophyta; class: Polypodiidae; order: Polypodiales; family: Pteridaceae; genus: Adiantum; specificEpithet: lunulatum; taxonRank: species; scientificNameAuthorship: Burm.; **Location:** continent: Africa; country: Togo; stateProvince: Centrale; decimalLatitude: 8.7; decimalLongitude: 0.7666667; geodeticDatum: WGS 1984; **Identification:** identifiedBy: J.-F. Brunel; dateIdentified: 7/8/1984; **Event:** eventDate: 7/8/1984; **Record Level:** institutionID: Herbarium togoense; collectionID: TOGO
- **Type status:** Other material. **Occurrence:** catalogNumber: 12007; recordNumber: 6920; recordedBy: J.-F. Brunel; lifeStage: Adult; reproductiveCondition: Fertile; **Taxon:** nameAccordingToID: The Plant List; scientificName: *Adiantum lunulatum* Burm.; kingdom: Plantae; phylum: Monilophyta; class: Polypodiidae; order: Polypodiales; family: Pteridaceae; genus: Adiantum; specificEpithet: lunulatum; taxonRank: species; scientificNameAuthorship: Burm.; **Location:** continent: Africa; country: Togo; stateProvince: Kara; decimalLatitude: 9.5155556; decimalLongitude: 1.04; geodeticDatum: WGS 1984; **Identification:** identifiedBy: J.-F. Brunel; **Record Level:** institutionID: Herbarium togoense; collectionID: TOGO
- **Type status:** Other material. **Occurrence:** catalogNumber: 12008; recordNumber: 533; recordedBy: K. Akpagana; lifeStage: Adult; reproductiveCondition: Fertile; **Taxon:** nameAccordingToID: The Plant List; scientificName: *Adiantum lunulatum* Burm.; kingdom: Plantae; phylum: Monilophyta; class: Polypodiidae; order: Polypodiales; family: Pteridaceae; genus: Adiantum; specificEpithet: lunulatum; taxonRank: species; scientificNameAuthorship: Burm.; **Location:** continent: Africa; country: Togo; stateProvince: Plateaux; decimalLatitude: 6.6666667; decimalLongitude: 1.1333333; geodeticDatum: WGS 1984; **Identification:** identifiedBy: K. Akpagana; dateIdentified: /7/1986; **Event:** eventDate: /7/1986; **Record Level:** institutionID: Herbarium togoense; collectionID: TOGO
- **Type status:** Other material. **Occurrence:** catalogNumber: 12011; recordNumber: 9101; recordedBy: J.-F. Brunel; lifeStage: Adult; reproductiveCondition: Fertile; **Taxon:** nameAccordingToID: The Plant List; scientificName: *Adiantum lunulatum* Burm.; kingdom: Plantae; phylum: Monilophyta; class: Polypodiidae; order: Polypodiales; family: Pteridaceae; genus: Adiantum; specificEpithet: lunulatum; taxonRank: species; scientificNameAuthorship: Burm.; **Location:** continent: Africa; country: Togo; stateProvince: Centrale; decimalLatitude: 8.75; decimalLongitude: 0.6666667; geodeticDatum: WGS 1984; **Identification:** identifiedBy: J.-F. Brunel; dateIdentified: /11/1984; **Event:** eventDate: /11/1984; **Record Level:** institutionID: Herbarium togoense; collectionID: TOGO
- **Type status:** Other material. **Occurrence:** catalogNumber: 12012; recordNumber: 1018; recordedBy: K. Akpagana; lifeStage: Adult; reproductiveCondition: Fertile; **Taxon:** nameAccordingToID: The Plant List; scientificName: *Adiantum lunulatum* Burm.; kingdom: Plantae; phylum: Monilophyta; class: Polypodiidae; order: Polypodiales; family: Pteridaceae; genus: Adiantum; specificEpithet: lunulatum; taxonRank: species; scientificNameAuthorship: Burm.; **Location:** continent: Africa; country: Togo; stateProvince: Centrale; decimalLatitude: 8.2166667; decimalLongitude: 0.8833333; geodeticDatum: WGS 1984; **Identification:** identifiedBy: K. Akpagana; dateIdentified: /10/1986; **Event:** eventDate: /10/1986; **Record Level:** institutionID: Herbarium togoense; collectionID: TOGO
- **Type status:** Other material. **Occurrence:** catalogNumber: 12015; recordNumber: 56bis; recordedBy: K. Akpagana; lifeStage: Adult; reproductiveCondition: Fertile; **Taxon:** nameAccordingToID: The Plant List; scientificName: *Adiantum lunulatum* Burm.; kingdom: Plantae; phylum: Monilophyta; class: Polypodiidae; order: Polypodiales; family: Pteridaceae; genus: Adiantum; specificEpithet: lunulatum; taxonRank: species; scientificNameAuthorship: Burm.; **Location:** continent: Africa; country: Togo; stateProvince: Centrale; decimalLatitude: 8.1833333; decimalLongitude: 0.65; geodeticDatum: WGS 1984; **Identification:** identifiedBy: K. Akpagana; dateIdentified: /10/1982; **Event:** eventDate: /10/1982; **Record Level:** institutionID: Herbarium togoense; collectionID: TOGO
- **Type status:** Other material. **Occurrence:** catalogNumber: 12016; recordNumber: 7795; recordedBy: J.-F. Brunel; lifeStage: Adult; reproductiveCondition: Fertile; **Taxon:** nameAccordingToID: The Plant List; scientificName: *Adiantum lunulatum* Burm.; kingdom: Plantae; phylum: Monilophyta; class: Polypodiidae; order: Polypodiales; family: Pteridaceae; genus: Adiantum; specificEpithet: lunulatum; taxonRank: species; scientificNameAuthorship: Burm.; **Location:** continent: Africa; country: Togo; stateProvince: Centrale; decimalLatitude: 8.05; decimalLongitude: 0.7833333; geodeticDatum: WGS 1984; **Identification:** identifiedBy: J.-F. Brunel; dateIdentified: /12/1982; **Event:** eventDate: /12/1982; **Record Level:** institutionID: Herbarium togoense; collectionID: TOGO
- **Type status:** Other material. **Occurrence:** catalogNumber: 12017; recordNumber: 27; recordedBy: K. Akpagana; lifeStage: Adult; reproductiveCondition: Fertile; **Taxon:** nameAccordingToID: The Plant List; scientificName: *Adiantum lunulatum* Burm.; kingdom: Plantae; phylum: Monilophyta; class: Polypodiidae; order: Polypodiales; family: Pteridaceae; genus: Adiantum; specificEpithet: lunulatum; taxonRank: species; scientificNameAuthorship: Burm.; **Location:** continent: Africa; country: Togo; stateProvince: Centrale; decimalLatitude: 8.05; decimalLongitude: 0.7833333; geodeticDatum: WGS 1984; **Identification:** identifiedBy: K. Akpagana; dateIdentified: /6/1982; **Event:** eventDate: /6/1982; **Record Level:** institutionID: Herbarium togoense; collectionID: TOGO
- **Type status:** Other material. **Occurrence:** catalogNumber: 12018; recordNumber: 7741; recordedBy: J.-F. Brunel; lifeStage: Adult; reproductiveCondition: Fertile; **Taxon:** nameAccordingToID: The Plant List; scientificName: *Adiantum lunulatum* Burm.; kingdom: Plantae; phylum: Monilophyta; class: Polypodiidae; order: Polypodiales; family: Pteridaceae; genus: Adiantum; specificEpithet: lunulatum; taxonRank: species; scientificNameAuthorship: Burm.; **Location:** continent: Africa; country: Togo; stateProvince: Centrale; decimalLatitude: 8.05; decimalLongitude: 0.7833333; geodeticDatum: WGS 1984; **Identification:** identifiedBy: J.-F. Brunel; dateIdentified: /6/1982; **Event:** eventDate: /6/1982; **Record Level:** institutionID: Herbarium togoense; collectionID: TOGO
- **Type status:** Other material. **Occurrence:** catalogNumber: 12019; recordNumber: 8009; recordedBy: J.-F. Brunel; lifeStage: Adult; reproductiveCondition: Fertile; **Taxon:** nameAccordingToID: The Plant List; scientificName: *Adiantum lunulatum* Burm.; kingdom: Plantae; phylum: Monilophyta; class: Polypodiidae; order: Polypodiales; family: Pteridaceae; genus: Adiantum; specificEpithet: lunulatum; taxonRank: species; scientificNameAuthorship: Burm.; **Location:** continent: Africa; country: Togo; stateProvince: Centrale; decimalLatitude: 8.7; decimalLongitude: 0.7666667; geodeticDatum: WGS 1984; **Identification:** identifiedBy: J.-F. Brunel; dateIdentified: /6/1983; **Event:** eventDate: /6/1983; **Record Level:** institutionID: Herbarium togoense; collectionID: TOGO
- **Type status:** Other material. **Occurrence:** catalogNumber: 12020; recordNumber: s.n.; recordedBy: Ayéna; lifeStage: Adult; reproductiveCondition: Fertile; **Taxon:** nameAccordingToID: The Plant List; scientificName: *Adiantum lunulatum* Burm.; kingdom: Plantae; phylum: Monilophyta; class: Polypodiidae; order: Polypodiales; family: Pteridaceae; genus: Adiantum; specificEpithet: lunulatum; taxonRank: species; scientificNameAuthorship: Burm.; **Location:** continent: Africa; country: Togo; stateProvince: Plateaux; decimalLatitude: 7; decimalLongitude: 0.75; geodeticDatum: WGS 1984; **Identification:** identifiedBy: J.-F. Brunel; dateIdentified: 24/3/1984; **Event:** eventDate: 24/3/1984; **Record Level:** institutionID: Herbarium togoense; collectionID: TOGO
- **Type status:** Other material. **Occurrence:** catalogNumber: 12021; recordNumber: 3524; recordedBy: Roussel; lifeStage: Adult; reproductiveCondition: Fertile; **Taxon:** nameAccordingToID: The Plant List; scientificName: *Adiantum lunulatum* Burm.; kingdom: Plantae; phylum: Monilophyta; class: Polypodiidae; order: Polypodiales; family: Pteridaceae; genus: Adiantum; specificEpithet: lunulatum; taxonRank: species; scientificNameAuthorship: Burm.; **Location:** continent: Africa; country: Togo; stateProvince: Centrale; decimalLatitude: 9.25; decimalLongitude: 1.2; geodeticDatum: WGS 1984; **Identification:** identifiedBy: Roussel; dateIdentified: 4/7/1987; **Event:** eventDate: 4/7/1987; **Record Level:** institutionID: Herbarium togoense; collectionID: TOGO
- **Type status:** Other material. **Occurrence:** catalogNumber: 12024; recordNumber: 99; recordedBy: J.-F. Brunel; lifeStage: Adult; reproductiveCondition: Fertile; **Taxon:** nameAccordingToID: The Plant List; scientificName: *Adiantum lunulatum* Burm.; kingdom: Plantae; phylum: Monilophyta; class: Polypodiidae; order: Polypodiales; family: Pteridaceae; genus: Adiantum; specificEpithet: lunulatum; taxonRank: species; scientificNameAuthorship: Burm.; **Location:** continent: Africa; country: Togo; stateProvince: Plateaux; decimalLatitude: 7.0166667; decimalLongitude: 0.65; geodeticDatum: WGS 1984; **Identification:** identifiedBy: C.A. Meyer; dateIdentified: /4/1980; **Event:** eventDate: 1973; **Record Level:** institutionID: Herbarium togoense; collectionID: TOGO
- **Type status:** Other material. **Occurrence:** catalogNumber: 12026; recordNumber: 113; recordedBy: K. Akpagana; lifeStage: Adult; reproductiveCondition: Fertile; **Taxon:** nameAccordingToID: The Plant List; scientificName: *Adiantum lunulatum* Burm.; kingdom: Plantae; phylum: Monilophyta; class: Polypodiidae; order: Polypodiales; family: Pteridaceae; genus: Adiantum; specificEpithet: lunulatum; taxonRank: species; scientificNameAuthorship: Burm.; **Location:** continent: Africa; country: Togo; stateProvince: Plateaux; decimalLatitude: 6.5833333; decimalLongitude: 0.75; geodeticDatum: WGS 1984; **Identification:** identifiedBy: K. Akpagana; dateIdentified: 1983; **Event:** eventDate: 1983; **Record Level:** institutionID: Herbarium togoense; collectionID: TOGO
- **Type status:** Other material. **Occurrence:** catalogNumber: 30034; recordNumber: 28; recordedBy: ABOTSI, SODJINOU & MINGOU; lifeStage: Adult; reproductiveCondition: Fertile; **Taxon:** nameAccordingToID: The Plant List; scientificName: *Adiantum lunulatum* Burm.; kingdom: Plantae; phylum: Monilophyta; class: Polypodiidae; order: Polypodiales; family: Pteridaceae; genus: Adiantum; specificEpithet: lunulatum; taxonRank: species; scientificNameAuthorship: Burm.; **Location:** continent: Africa; country: Togo; stateProvince: Plateaux; decimalLatitude: 7.51470436; decimalLongitude: 0.59350388; geodeticDatum: WGS 1984; **Identification:** identifiedBy: K. E. Abotsi; dateIdentified: /05/2013; **Event:** eventDate: 04-03-13; **Record Level:** institutionID: Herbarium togoense; collectionID: TOGO
- **Type status:** Other material. **Occurrence:** catalogNumber: 30044; recordNumber: 84; recordedBy: ABOTSI, SODJINOU & MINGOU; lifeStage: Adult; reproductiveCondition: Fertile; **Taxon:** nameAccordingToID: The Plant List; scientificName: *Adiantum lunulatum* Burm.; kingdom: Plantae; phylum: Monilophyta; class: Polypodiidae; order: Polypodiales; family: Pteridaceae; genus: Adiantum; specificEpithet: lunulatum; taxonRank: species; scientificNameAuthorship: Burm.; **Location:** continent: Africa; country: Togo; stateProvince: Plateaux; decimalLatitude: 7.51365336; decimalLongitude: 0.61454758; geodeticDatum: WGS 1984; **Identification:** identifiedBy: K. E. Abotsi; dateIdentified: /05/2013; **Event:** eventDate: 04-04-13; **Record Level:** institutionID: Herbarium togoense; collectionID: TOGO
- **Type status:** Other material. **Occurrence:** catalogNumber: 30046; recordNumber: 91; recordedBy: ABOTSI, SODJINOU & MINGOU; lifeStage: Adult; reproductiveCondition: Fertile; **Taxon:** nameAccordingToID: The Plant List; scientificName: *Adiantum lunulatum* Burm.; kingdom: Plantae; phylum: Monilophyta; class: Polypodiidae; order: Polypodiales; family: Pteridaceae; genus: Adiantum; specificEpithet: lunulatum; taxonRank: species; scientificNameAuthorship: Burm.; **Location:** continent: Africa; country: Togo; stateProvince: Plateaux; decimalLatitude: 7.51402623; decimalLongitude: 0.6149712; geodeticDatum: WGS 1984; **Identification:** identifiedBy: K. E. Abotsi; dateIdentified: /05/2013; **Event:** eventDate: 04-04-13; **Record Level:** institutionID: Herbarium togoense; collectionID: TOGO
- **Type status:** Other material. **Occurrence:** catalogNumber: 30049; recordNumber: 96; recordedBy: ABOTSI, SODJINOU & MINGOU; lifeStage: Adult; reproductiveCondition: Fertile; **Taxon:** nameAccordingToID: The Plant List; scientificName: *Adiantum lunulatum* Burm.; kingdom: Plantae; phylum: Monilophyta; class: Polypodiidae; order: Polypodiales; family: Pteridaceae; genus: Adiantum; specificEpithet: lunulatum; taxonRank: species; scientificNameAuthorship: Burm.; **Location:** continent: Africa; country: Togo; stateProvince: Plateaux; decimalLatitude: 7.51544442; decimalLongitude: 0.61815133; geodeticDatum: WGS 1984; **Identification:** identifiedBy: K. E. Abotsi; dateIdentified: /05/2013; **Event:** eventDate: 04-04-13; **Record Level:** institutionID: Herbarium togoense; collectionID: TOGO
- **Type status:** Other material. **Occurrence:** catalogNumber: 30058; recordNumber: 130; recordedBy: ABOTSI, SODJINOU & MINGOU; lifeStage: Adult; reproductiveCondition: Fertile; **Taxon:** nameAccordingToID: The Plant List; scientificName: *Adiantum lunulatum* Burm.; kingdom: Plantae; phylum: Monilophyta; class: Polypodiidae; order: Polypodiales; family: Pteridaceae; genus: Adiantum; specificEpithet: lunulatum; taxonRank: species; scientificNameAuthorship: Burm.; **Location:** continent: Africa; country: Togo; stateProvince: Plateaux; decimalLatitude: 8.17923156; decimalLongitude: 0.65314643; geodeticDatum: WGS 1984; **Identification:** identifiedBy: K. E. Abotsi; dateIdentified: /05/2013; **Event:** eventDate: 05-08-13; **Record Level:** institutionID: Herbarium togoense; collectionID: TOGO
- **Type status:** Other material. **Occurrence:** catalogNumber: 30072; recordNumber: 192; recordedBy: ABOTSI, SODJINOU & MINGOU; lifeStage: Adult; reproductiveCondition: Fertile; **Taxon:** nameAccordingToID: The Plant List; scientificName: *Adiantum lunulatum* Burm.; kingdom: Plantae; phylum: Monilophyta; class: Polypodiidae; order: Polypodiales; family: Pteridaceae; genus: Adiantum; specificEpithet: lunulatum; taxonRank: species; scientificNameAuthorship: Burm.; **Location:** continent: Africa; country: Togo; stateProvince: Plateaux; decimalLatitude: 8.01912192; decimalLongitude: 0.6313327; geodeticDatum: WGS 1984; **Identification:** identifiedBy: K. E. Abotsi; dateIdentified: /05/2013; **Event:** eventDate: 05-11-13; **Record Level:** institutionID: Herbarium togoense; collectionID: TOGO
- **Type status:** Other material. **Occurrence:** catalogNumber: 30082; recordNumber: 273; recordedBy: ABOTSI, SODJINOU & MINGOU; lifeStage: Adult; reproductiveCondition: Fertile; **Taxon:** nameAccordingToID: The Plant List; scientificName: *Adiantum lunulatum* Burm.; kingdom: Plantae; phylum: Monilophyta; class: Polypodiidae; order: Polypodiales; family: Pteridaceae; genus: Adiantum; specificEpithet: lunulatum; taxonRank: species; scientificNameAuthorship: Burm.; **Location:** continent: Africa; country: Togo; stateProvince: Plateaux; decimalLatitude: 7.1058468; decimalLongitude: 0.60876719; geodeticDatum: WGS 1984; **Identification:** identifiedBy: K. E. Abotsi; dateIdentified: /05/2013; **Event:** eventDate: 04-11-13; **Record Level:** institutionID: Herbarium togoense; collectionID: TOGO
- **Type status:** Other material. **Occurrence:** catalogNumber: 30091; recordNumber: 294; recordedBy: ABOTSI, SODJINOU & MINGOU; lifeStage: Adult; reproductiveCondition: Fertile; **Taxon:** nameAccordingToID: The Plant List; scientificName: *Adiantum lunulatum* Burm.; kingdom: Plantae; phylum: Monilophyta; class: Polypodiidae; order: Polypodiales; family: Pteridaceae; genus: Adiantum; specificEpithet: lunulatum; taxonRank: species; scientificNameAuthorship: Burm.; **Location:** continent: Africa; country: Togo; stateProvince: Plateaux; decimalLatitude: 6.9544104; decimalLongitude: 0.58024464; geodeticDatum: WGS 1984; **Identification:** identifiedBy: K. E. Abotsi; dateIdentified: /05/2013; **Event:** eventDate: 04-15-13; **Record Level:** institutionID: Herbarium togoense; collectionID: TOGO
- **Type status:** Other material. **Occurrence:** catalogNumber: 30094; recordNumber: 302; recordedBy: ABOTSI, SODJINOU & MINGOU; lifeStage: Adult; reproductiveCondition: Fertile; **Taxon:** nameAccordingToID: The Plant List; scientificName: *Adiantum lunulatum* Burm.; kingdom: Plantae; phylum: Monilophyta; class: Polypodiidae; order: Polypodiales; family: Pteridaceae; genus: Adiantum; specificEpithet: lunulatum; taxonRank: species; scientificNameAuthorship: Burm.; **Location:** continent: Africa; country: Togo; stateProvince: Plateaux; decimalLatitude: 6.94609034; decimalLongitude: 0.57926515; geodeticDatum: WGS 1984; **Identification:** identifiedBy: K. E. Abotsi; dateIdentified: /05/2013; **Event:** eventDate: 04-15-13; **Record Level:** institutionID: Herbarium togoense; collectionID: TOGO
- **Type status:** Other material. **Occurrence:** catalogNumber: 30098; recordNumber: 308; recordedBy: ABOTSI, SODJINOU & MINGOU; lifeStage: Adult; reproductiveCondition: Fertile; **Taxon:** nameAccordingToID: The Plant List; scientificName: *Adiantum lunulatum* Burm.; kingdom: Plantae; phylum: Monilophyta; class: Polypodiidae; order: Polypodiales; family: Pteridaceae; genus: Adiantum; specificEpithet: lunulatum; taxonRank: species; scientificNameAuthorship: Burm.; **Location:** continent: Africa; country: Togo; stateProvince: Plateaux; decimalLatitude: 6.84914487; decimalLongitude: 0.74784103; geodeticDatum: WGS 1984; **Identification:** identifiedBy: K. E. Abotsi; dateIdentified: /05/2013; **Event:** eventDate: 04-16-13; **Record Level:** institutionID: Herbarium togoense; collectionID: TOGO
- **Type status:** Other material. **Occurrence:** catalogNumber: 30102; recordNumber: 323; recordedBy: ABOTSI, SODJINOU & MINGOU; lifeStage: Adult; reproductiveCondition: Fertile; **Taxon:** nameAccordingToID: The Plant List; scientificName: *Adiantum lunulatum* Burm.; kingdom: Plantae; phylum: Monilophyta; class: Polypodiidae; order: Polypodiales; family: Pteridaceae; genus: Adiantum; specificEpithet: lunulatum; taxonRank: species; scientificNameAuthorship: Burm.; **Location:** continent: Africa; country: Togo; stateProvince: Plateaux; decimalLatitude: 6.87057362; decimalLongitude: 0.74717051; geodeticDatum: WGS 1984; **Identification:** identifiedBy: K. E. Abotsi; dateIdentified: /05/2013; **Event:** eventDate: 04-16-13; **Record Level:** institutionID: Herbarium togoense; collectionID: TOGO
- **Type status:** Other material. **Occurrence:** catalogNumber: 30103; recordNumber: 339; recordedBy: ABOTSI, SODJINOU & MINGOU; lifeStage: Adult; reproductiveCondition: Fertile; **Taxon:** nameAccordingToID: The Plant List; scientificName: *Adiantum lunulatum* Burm.; kingdom: Plantae; phylum: Monilophyta; class: Polypodiidae; order: Polypodiales; family: Pteridaceae; genus: Adiantum; specificEpithet: lunulatum; taxonRank: species; scientificNameAuthorship: Burm.; **Location:** continent: Africa; country: Togo; stateProvince: Plateaux; decimalLatitude: 8.01889124; decimalLongitude: 0.62737159; geodeticDatum: WGS 1984; **Identification:** identifiedBy: K. E. Abotsi; dateIdentified: /05/2013; **Event:** eventDate: 05-11-13; **Record Level:** institutionID: Herbarium togoense; collectionID: TOGO

##### Ecological interactions

###### Native status

Native

##### Distribution

Togo (Ecological Zones 3 and 4), China, Taiwan, Australia, Jammu & Kashmir, India, Andaman Isl., Myanmar, Philippines, Thailand, Laos, Cambodia, Vietnam, peninsular Malaysia, Sulawesi, Sri Lanka, Nepal, Moluccas, Oman, S-Yemen, Zimbabwe, Mozambique, South Africa, Zambia, Malawi, Chad, Tanzania, Sao Tomé, Bioko Isl., Senegal, Guinea-Bissau, Guinea, Sierra Leone, Liberia, Ivory Coast, Ghana, Benin, Niger, Nigeria, Cameroon, Central African Republic, Sao Tome, Congo, D.R.Congo, Angola, Ethiopia, Sudan, Madagascar, Comores, Burkina Faso, Mali, Sudan, Southern Marianas, Fiji, Micronesia, Palau Isl., Western Samoa, Cuba, Cape Verde Isl.

##### Notes

*Adiantum lunulatum* has a short rhizome, slightly erect or creeping, wearing dark brown scales of about 3 mm long. The fronds are arched and arranged in tufts (Fig. 7a). The petiole is black, shiny, hairless, 10 to over 15 cm long. The lamina is lanceolate, pinnate and has a herbaceous texture. The pinnae are alternate, long-stalked, semi-elliptical, dimidiate (Fig. 7b). Their upper base is truncated. Their top margin is serrated on the sterile frond but slightly lobed on the fertile frond. The terminal pinnae is obtriangular (Fig. 7c). The sori are crescent shaped (Fig. 7d). *Adiantum lunulatum* grows almost everywhere, in the shade of trees, on various soils, with sufficient moisture.

#### Adiantum schweinfurthii

Kuhn.

Adiantum chevalieri Christ.

##### Materials

- **Type status:** Other material. **Occurrence:** catalogNumber: 11991; recordNumber: 8010; recordedBy: J.-F. Brunel; lifeStage: Adult; reproductiveCondition: Fertile; **Taxon:** nameAccordingToID: The Plant List; scientificName: *Adiantum schweinfurthii* Kuhn; kingdom: Plantae; phylum: Monilophyta; class: Polypodiidae; order: Polypodiales; family: Pteridaceae; genus: Adiantum; specificEpithet: schweinfurthii; taxonRank: species; scientificNameAuthorship: Kuhn.; **Location:** continent: Africa; country: Togo; stateProvince: Centrale; decimalLatitude: 8.7; decimalLongitude: 0.7666667; geodeticDatum: WGS 1984; **Identification:** identifiedBy: J.-F. Brunel; dateIdentified: /6/1983; **Event:** eventDate: /6/1983; **Record Level:** institutionID: Herbarium togoense; collectionID: TOGO
- **Type status:** Other material. **Occurrence:** catalogNumber: 12004; recordNumber: 8688; recordedBy: J.-F. Brunel; lifeStage: Adult; reproductiveCondition: Fertile; **Taxon:** nameAccordingToID: The Plant List; scientificName: *Adiantum schweinfurthii* Kuhn; kingdom: Plantae; phylum: Monilophyta; class: Polypodiidae; order: Polypodiales; family: Pteridaceae; genus: Adiantum; specificEpithet: schweinfurthii; taxonRank: species; scientificNameAuthorship: Kuhn.; **Location:** continent: Africa; country: Togo; stateProvince: Centrale; decimalLatitude: 8.7; decimalLongitude: 0.7666667; geodeticDatum: WGS 1984; **Identification:** identifiedBy: J.-F. Brunel; dateIdentified: /9/1984; **Event:** eventDate: /9/1984; **Record Level:** institutionID: Herbarium togoense; collectionID: TOGO
- **Type status:** Other material. **Occurrence:** catalogNumber: 12022; recordNumber: 1086; recordedBy: K. Akpagana; lifeStage: Adult; reproductiveCondition: Fertile; **Taxon:** nameAccordingToID: The Plant List; scientificName: *Adiantum schweinfurthii* Kuhn; kingdom: Plantae; phylum: Monilophyta; class: Polypodiidae; order: Polypodiales; family: Pteridaceae; genus: Adiantum; specificEpithet: schweinfurthii; taxonRank: species; scientificNameAuthorship: Kuhn.; **Location:** continent: Africa; country: Togo; stateProvince: Centrale; decimalLatitude: 8.2166667; decimalLongitude: 0.8833333; geodeticDatum: WGS 1984; **Identification:** identifiedBy: K. Akpagana; dateIdentified: /10/1986; **Event:** eventDate: /10/1986; **Record Level:** institutionID: Herbarium togoense; collectionID: TOGO
- **Type status:** Other material. **Occurrence:** catalogNumber: 30073; recordNumber: 193; recordedBy: ABOTSI, SODJINOU & MINGOU; lifeStage: Adult; reproductiveCondition: Fertile; **Taxon:** nameAccordingToID: The Plant List; scientificName: *Adiantum schweinfurthii* Kuhn; kingdom: Plantae; phylum: Monilophyta; class: Polypodiidae; order: Polypodiales; family: Pteridaceae; genus: Adiantum; specificEpithet: schweinfurthii; taxonRank: species; scientificNameAuthorship: Kuhn.; **Location:** continent: Africa; country: Togo; stateProvince: Plateaux; decimalLatitude: 8.01949931; decimalLongitude: 0.63094968; geodeticDatum: WGS 1984; **Identification:** identifiedBy: K. E. Abotsi; dateIdentified: /05/2013; **Event:** eventDate: 05-11-13; **Record Level:** institutionID: Herbarium togoense; collectionID: TOGO

##### Ecological interactions

###### Native status

Native

##### Distribution

Togo (Ecological Zones 2 and 4), Tanzania, Chad, Mali, Senegal, Sudan, Central African Republic, Guinea, Nigeria, D.R.Congo, Angola, Cameroon

##### Notes

*Adiantum schweinfurthii* is a fern with a short rhizome, with brown scales, linear-lanceolate, long-acuminate, entire. The fronds are in clumps, pinnate, oblong-lanceolate, 12-34 cm long (Fig. 8a). The stem and rachis are purple-black and shiny, hairless. The pinnae are pale green, oblong or oblong-lanceolate, 1 to 2.2 cm long and 3-8 mm wide, oblique at the base; higher margins are lobed and serrated but not deeply incised. The lower margin is entire and glabrous. Each pinnae has 4-7 pairs of oblong-reniform sori, often 1 by a lobule (Fig. 8b).

#### Adiantum vogelii

Mett. ex Keyserl.

Adiantum prionophyllum var. *γ* Hook., *A. tetraphyllum* sensu Hook & Bak., A. tetraphyllum var. obtusum Kuhn., A. tetraphyllum var. vogelii (Keyserl.) Bonap.

##### Materials

- **Type status:** Other material. **Occurrence:** catalogNumber: 11992; recordNumber: 8178; recordedBy: J.-F. Brunel; lifeStage: Adult; reproductiveCondition: Fertile; **Taxon:** nameAccordingToID: The Plant List; scientificName: *Adiantum vogelii* Mett. ex Keyserl.; kingdom: Plantae; phylum: Monilophyta; class: Polypodiidae; order: Polypodiales; family: Pteridaceae; genus: Adiantum; specificEpithet: vogelii; taxonRank: species; scientificNameAuthorship: Mett. ex Keyserl.; **Location:** continent: Africa; country: Togo; stateProvince: Plateaux; decimalLatitude: 6.6666667; decimalLongitude: 1.1333333; geodeticDatum: WGS 1984; **Identification:** identifiedBy: J.-F. Brunel; dateIdentified: /1/1984; **Event:** eventDate: /1/1984; **Record Level:** institutionID: Herbarium togoense; collectionID: TOGO
- **Type status:** Other material. **Occurrence:** catalogNumber: 11994; recordNumber: 4859; recordedBy: J.-F. Brunel; lifeStage: Adult; reproductiveCondition: Fertile; **Taxon:** nameAccordingToID: The Plant List; scientificName: *Adiantum vogelii* Mett. ex Keyserl.; kingdom: Plantae; phylum: Monilophyta; class: Polypodiidae; order: Polypodiales; family: Pteridaceae; genus: Adiantum; specificEpithet: vogelii; taxonRank: species; scientificNameAuthorship: Mett. ex Keyserl.; **Location:** continent: Africa; country: Togo; stateProvince: Plateaux; decimalLatitude: 7; decimalLongitude: 0.75; geodeticDatum: WGS 1984; **Identification:** identifiedBy: C.A. Meyer; dateIdentified: /4/1980; **Event:** eventDate: /3/1978; **Record Level:** institutionID: Herbarium togoense; collectionID: TOGO
- **Type status:** Other material. **Occurrence:** catalogNumber: 11995; recordNumber: 72; recordedBy: J.-F. Brunel; lifeStage: Adult; reproductiveCondition: Fertile; **Taxon:** nameAccordingToID: The Plant List; scientificName: *Adiantum vogelii* Mett. ex Keyserl.; kingdom: Plantae; phylum: Monilophyta; class: Polypodiidae; order: Polypodiales; family: Pteridaceae; genus: Adiantum; specificEpithet: vogelii; taxonRank: species; scientificNameAuthorship: Mett. ex Keyserl.; **Location:** continent: Africa; country: Togo; stateProvince: Plateaux; decimalLatitude: 7; decimalLongitude: 0.75; geodeticDatum: WGS 1984; **Identification:** identifiedBy: C.A. Meyer; dateIdentified: /4/1980; **Event:** eventDate: /1/1973; **Record Level:** institutionID: Herbarium togoense; collectionID: TOGO
- **Type status:** Other material. **Occurrence:** catalogNumber: 11997; recordNumber: 25; recordedBy: K. Akpagana; lifeStage: Adult; reproductiveCondition: Fertile; **Taxon:** nameAccordingToID: The Plant List; scientificName: *Adiantum vogelii* Mett. ex Keyserl.; kingdom: Plantae; phylum: Monilophyta; class: Polypodiidae; order: Polypodiales; family: Pteridaceae; genus: Adiantum; specificEpithet: vogelii; taxonRank: species; scientificNameAuthorship: Mett. ex Keyserl.; **Location:** continent: Africa; country: Togo; stateProvince: Centrale; decimalLatitude: 8.05; decimalLongitude: 0.7833333; geodeticDatum: WGS 1984; **Identification:** identifiedBy: K. Akpagana; dateIdentified: /6/1982; **Event:** eventDate: /6/1982; **Record Level:** institutionID: Herbarium togoense; collectionID: TOGO
- **Type status:** Other material. **Occurrence:** catalogNumber: 11999; recordNumber: 524; recordedBy: K. Akpagana; lifeStage: Adult; reproductiveCondition: Fertile; **Taxon:** nameAccordingToID: The Plant List; scientificName: *Adiantum vogelii* Mett. ex Keyserl.; kingdom: Plantae; phylum: Monilophyta; class: Polypodiidae; order: Polypodiales; family: Pteridaceae; genus: Adiantum; specificEpithet: vogelii; taxonRank: species; scientificNameAuthorship: Mett. ex Keyserl.; **Location:** continent: Africa; country: Togo; stateProvince: Plateaux; decimalLatitude: 6.6666667; decimalLongitude: 1.1333333; geodeticDatum: WGS 1984; **Identification:** identifiedBy: K. Akpagana; dateIdentified: /7/1986; **Event:** eventDate: /7/1986; **Record Level:** institutionID: Herbarium togoense; collectionID: TOGO
- **Type status:** Other material. **Occurrence:** catalogNumber: 30028; recordNumber: 13; recordedBy: ABOTSI, SODJINOU & MINGOU; lifeStage: Adult; reproductiveCondition: Fertile; **Taxon:** nameAccordingToID: The Plant List; scientificName: *Adiantum vogelii* Mett. ex Keyserl.; kingdom: Plantae; phylum: Monilophyta; class: Polypodiidae; order: Polypodiales; family: Pteridaceae; genus: Adiantum; specificEpithet: vogelii; taxonRank: species; scientificNameAuthorship: Mett. ex Keyserl.; **Location:** continent: Africa; country: Togo; stateProvince: Plateaux; decimalLatitude: 7.513791; decimalLongitude: 0.59505754; geodeticDatum: WGS 1984; **Identification:** identifiedBy: K. E. Abotsi; dateIdentified: /05/2013; **Event:** eventDate: 04-03-13; **Record Level:** institutionID: Herbarium togoense; collectionID: TOGO
- **Type status:** Other material. **Occurrence:** catalogNumber: 30029; recordNumber: 14; recordedBy: ABOTSI, SODJINOU & MINGOU; lifeStage: Adult; reproductiveCondition: Fertile; **Taxon:** nameAccordingToID: The Plant List; scientificName: *Adiantum vogelii* Mett. ex Keyserl.; kingdom: Plantae; phylum: Monilophyta; class: Polypodiidae; order: Polypodiales; family: Pteridaceae; genus: Adiantum; specificEpithet: vogelii; taxonRank: species; scientificNameAuthorship: Mett. ex Keyserl.; **Location:** continent: Africa; country: Togo; stateProvince: Plateaux; decimalLatitude: 7.513791; decimalLongitude: 0.59505754; geodeticDatum: WGS 1984; **Identification:** identifiedBy: K. E. Abotsi; dateIdentified: /05/2013; **Event:** eventDate: 04-03-13; **Record Level:** institutionID: Herbarium togoense; collectionID: TOGO
- **Type status:** Other material. **Occurrence:** catalogNumber: 30030; recordNumber: 15; recordedBy: ABOTSI, SODJINOU & MINGOU; lifeStage: Adult; reproductiveCondition: Fertile; **Taxon:** nameAccordingToID: The Plant List; scientificName: *Adiantum vogelii* Mett. ex Keyserl.; kingdom: Plantae; phylum: Monilophyta; class: Polypodiidae; order: Polypodiales; family: Pteridaceae; genus: Adiantum; specificEpithet: vogelii; taxonRank: species; scientificNameAuthorship: Mett. ex Keyserl.; **Location:** continent: Africa; country: Togo; stateProvince: Plateaux; decimalLatitude: 7.513791; decimalLongitude: 0.59505754; geodeticDatum: WGS 1984; **Identification:** identifiedBy: K. E. Abotsi; dateIdentified: /05/2013; **Event:** eventDate: 04-03-13; **Record Level:** institutionID: Herbarium togoense; collectionID: TOGO
- **Type status:** Other material. **Occurrence:** catalogNumber: 30056; recordNumber: 126; recordedBy: ABOTSI, SODJINOU & MINGOU; lifeStage: Adult; reproductiveCondition: Fertile; **Taxon:** nameAccordingToID: The Plant List; scientificName: *Adiantum vogelii* Mett. ex Keyserl.; kingdom: Plantae; phylum: Monilophyta; class: Polypodiidae; order: Polypodiales; family: Pteridaceae; genus: Adiantum; specificEpithet: vogelii; taxonRank: species; scientificNameAuthorship: Mett. ex Keyserl.; **Location:** continent: Africa; country: Togo; stateProvince: Plateaux; decimalLatitude: 8.17550638; decimalLongitude: 0.65912791; geodeticDatum: WGS 1984; **Identification:** identifiedBy: K. E. Abotsi; dateIdentified: /05/2013; **Event:** eventDate: 05-08-13; **Record Level:** institutionID: Herbarium togoense; collectionID: TOGO
- **Type status:** Other material. **Occurrence:** catalogNumber: 30064; recordNumber: 160; recordedBy: ABOTSI, SODJINOU & MINGOU; lifeStage: Adult; reproductiveCondition: Fertile; **Taxon:** nameAccordingToID: The Plant List; scientificName: *Adiantum vogelii* Mett. ex Keyserl.; kingdom: Plantae; phylum: Monilophyta; class: Polypodiidae; order: Polypodiales; family: Pteridaceae; genus: Adiantum; specificEpithet: vogelii; taxonRank: species; scientificNameAuthorship: Mett. ex Keyserl.; **Location:** continent: Africa; country: Togo; stateProvince: Plateaux; decimalLatitude: 8.19652029; decimalLongitude: 0.61902733; geodeticDatum: WGS 1984; **Identification:** identifiedBy: K. E. Abotsi; dateIdentified: /05/2013; **Event:** eventDate: 05-09-13; **Record Level:** institutionID: Herbarium togoense; collectionID: TOGO
- **Type status:** Other material. **Occurrence:** catalogNumber: 30105; recordNumber: 344; recordedBy: ABOTSI, SODJINOU & MINGOU; lifeStage: Adult; reproductiveCondition: Fertile; **Taxon:** nameAccordingToID: The Plant List; scientificName: *Adiantum vogelii* Mett. ex Keyserl.; kingdom: Plantae; phylum: Monilophyta; class: Polypodiidae; order: Polypodiales; family: Pteridaceae; genus: Adiantum; specificEpithet: vogelii; taxonRank: species; scientificNameAuthorship: Mett. ex Keyserl.; **Location:** continent: Africa; country: Togo; stateProvince: Plateaux; decimalLatitude: 8.02205753; decimalLongitude: 0.62162262; geodeticDatum: WGS 1984; **Identification:** identifiedBy: K. E. Abotsi; dateIdentified: /05/2013; **Event:** eventDate: 05-11-13; **Record Level:** institutionID: Herbarium togoense; collectionID: TOGO

##### Ecological interactions

###### Native status

Native

##### Distribution

Togo (Ecological Zones 4 and 5), Uganda, Zanzibar, Principe Isl., Bioko Isl., Senegal, Guinea, Sierra Leone, Liberia, Ivory Coast, Ghana, Benin, Nigeria, Cameroon, Central African Republic, Equatorial Guinea, Gabon, Congo, D.R.Congo, Angola

##### Notes

*Adiantum vogelii* is a fairly large fern with developed creeping rhizome, up to 70 cm long, bearing piliform black scales of 2 to 3 mm long (Fig. 9a). The fronds are between 20 and 60 cm long and are closely spaced. The petiole is blackish, channeled, 5 to 25 cm long, usually angular, hairy. The rachis, not winged, is shaggy. The lamina is bipinnate, deltoid-lanceolate and measures 10-15 cm for 8-10 cm wide. Pinnae and pinnules are alternate. The lamina is composed of 2 to 4 pairs of lateral pinnae similar to the terminal, short-stalked, linear, 8-10 cm long and 2-3 cm wide, with deltoid apex, shortly tapered. The pinnae are rhomboid-dimidiate, with upper base incised on about 1/3 of its width. The lobes are truncated and toothed. The ribs are single or bifurcated towards the lower 1/3. The sori are crescent shaped (Fig. 9b). *Adiantum vogelii* generally grows in forests, on fairly moist and rich soils.

#### Ceratopteris thalictroides

(L.) Brongn.

Acrostichum siliquosum L., *Pteris thalictroides* (L.) Sw., *P. siliquosum* (L.) P. Beauv., *P. cornuta* P. Beauv., *Ceratopteris gaudichaudii* Brongn., *C. cornuta* (P. Beauv.) Lepr, *C. siliquosa* (L.) Copel, C. thalictroides Schelpe var. thalictroides, C. thalictroides var. cornuta (P. Beauv.) Schelpe

##### Materials

- **Type status:** Other material. **Occurrence:** catalogNumber: 11963; recordNumber: 4671; recordedBy: K. Kulo; lifeStage: Adult; reproductiveCondition: Fertile; **Taxon:** nameAccordingToID: The Plant List; scientificName: *Ceratopteris thalictroides* (L.) Brongn.; kingdom: Plantae; phylum: Monilophyta; class: Polypodiidae; order: Polypodiales; family: Pteridaceae; genus: Ceratopteris; specificEpithet: thalictroides; taxonRank: species; scientificNameAuthorship: (L.) Brongn.; **Location:** continent: Africa; country: Togo; stateProvince: Maritime; decimalLatitude: 6.3422222; decimalLongitude: 1.1116667; geodeticDatum: WGS 1984; **Identification:** identifiedBy: J.-F. Brunel; dateIdentified: /4/1978; **Event:** eventDate: /4/1978; **Record Level:** institutionID: Herbarium togoense; collectionID: TOGO
- **Type status:** Other material. **Occurrence:** catalogNumber: 11964; recordNumber: 60; recordedBy: K. Kokou; lifeStage: Adult; reproductiveCondition: Fertile; **Taxon:** nameAccordingToID: The Plant List; scientificName: *Ceratopteris thalictroides* (L.) Brongn.; kingdom: Plantae; phylum: Monilophyta; class: Polypodiidae; order: Polypodiales; family: Pteridaceae; genus: Ceratopteris; specificEpithet: thalictroides; taxonRank: species; scientificNameAuthorship: (L.) Brongn.; **Location:** continent: Africa; country: Togo; stateProvince: Plateaux; decimalLatitude: 7.4333333; decimalLongitude: 1.4166667; geodeticDatum: WGS 1984; **Identification:** identifiedBy: K. Kokou; dateIdentified: 8/11/1989; **Event:** eventDate: 8/11/1989; **Record Level:** institutionID: Herbarium togoense; collectionID: TOGO
- **Type status:** Other material. **Occurrence:** catalogNumber: 11965; recordNumber: 86bis; recordedBy: K. Akpagana; lifeStage: Adult; reproductiveCondition: Fertile; **Taxon:** nameAccordingToID: The Plant List; scientificName: *Ceratopteris thalictroides* (L.) Brongn.; kingdom: Plantae; phylum: Monilophyta; class: Polypodiidae; order: Polypodiales; family: Pteridaceae; genus: Ceratopteris; specificEpithet: thalictroides; taxonRank: species; scientificNameAuthorship: (L.) Brongn.; **Location:** continent: Africa; country: Togo; stateProvince: Plateaux; decimalLatitude: 7.4333333; decimalLongitude: 1.4166667; geodeticDatum: WGS 1984; **Identification:** identifiedBy: K. Akpagana; dateIdentified: 1982; **Event:** eventDate: 1982; **Record Level:** institutionID: Herbarium togoense; collectionID: TOGO
- **Type status:** Other material. **Occurrence:** catalogNumber: 11966; recordNumber: 63; recordedBy: K. Kokou; lifeStage: Adult; reproductiveCondition: Fertile; **Taxon:** nameAccordingToID: The Plant List; scientificName: *Ceratopteris thalictroides* (L.) Brongn.; kingdom: Plantae; phylum: Monilophyta; class: Polypodiidae; order: Polypodiales; family: Pteridaceae; genus: Ceratopteris; specificEpithet: thalictroides; taxonRank: species; scientificNameAuthorship: (L.) Brongn.; **Location:** continent: Africa; country: Togo; stateProvince: Plateaux; decimalLatitude: 7.4333333; decimalLongitude: 1.4166667; geodeticDatum: WGS 1984; **Identification:** identifiedBy: K. Kokou; dateIdentified: 8/11/1989; **Event:** eventDate: 8/11/1989; **Record Level:** institutionID: Herbarium togoense; collectionID: TOGO
- **Type status:** Other material. **Occurrence:** catalogNumber: 11969; recordNumber: 7677; recordedBy: J.-F. Brunel; lifeStage: Adult; reproductiveCondition: Fertile; **Taxon:** nameAccordingToID: The Plant List; scientificName: *Ceratopteris thalictroides* (L.) Brongn.; kingdom: Plantae; phylum: Monilophyta; class: Polypodiidae; order: Polypodiales; family: Pteridaceae; genus: Ceratopteris; specificEpithet: thalictroides; taxonRank: species; scientificNameAuthorship: (L.) Brongn.; **Location:** continent: Africa; country: Togo; stateProvince: Plateaux; decimalLatitude: 7.4333333; decimalLongitude: 1.4166667; geodeticDatum: WGS 1984; **Identification:** identifiedBy: J.-F. Brunel; dateIdentified: /6/1982; **Event:** eventDate: /6/1977; **Record Level:** institutionID: Herbarium togoense; collectionID: TOGO

##### Ecological interactions

###### Native status

Native

###### Conservation status

Least Concern

##### Distribution

Togo (Ecological Zones 2, 3 and 5), USA, Costa Rica, Nicaragua, Mexico, Guatemala, El Salvador, Panama, Colombia, Venezuela, Puerto Rico, Jamaica, Hispaniola, Ecuador, Brazil, Guyana, Surinam, French Guiana, Australia, Taiwan, China, Japan, Ryukyu Isl., India, Andaman Isl., Nicobar Isl., Sri Lanka, Myanmar, Nepal, Philippines, Vietnam, Thailand, Laos, Cambodia, Sulawesi, Hawaii (I) (Kauai (I), Oahu (I)), Fiji (I), Micronesia, Palau Isl., Southern Marianas, Uganda, Tanzania, Kenya, Zanzibar, Somalia, Sudan, Congo, Angola, Zambia, Zimbabwe, Mozambique, Ethiopia

##### Notes

*Ceratopteris thalictroides* is an annual aquatic fern, with dimorphic pale-green fronds, irregularly pinnate. Sterile fronds are wider than the fertile fronds. They have a thin texture and are traversed by anastomosing veins. The sterile fronds have a stem of 8 to 25 cm long, ovate to deltoid lamina, 20 to 40 cm long and 7-30 cm wide, pinnate, pinnatifid or 2-3-pinnatifid, with winding lobes. The terminal segments are triangular to lanceolate, hairless. The fertile fronds have a stem of more than 40 cm long, a limb of 24-50 cm long and 12-30 cm wide, 2-4-pinnate, with linear ultimate segments of more than 4 cm long for 1-2 mm wide, glabrous. The sporangia are located along the veins. They are protected by a marginal entire pseudo-indusium, membranous, formed by the reflected margin of the lamina, with 30-70 thickened cells (Fig. 10). *Ceratopteris thalictroides* usually grows in marshies and low flowing rivers.

#### Doryopteris concolorvar.nicklesii

(Tard) Schelpe.

Doryopteris nicklesii Tardieu., *Pellaea geraniifolia* (Raddi) Fée

##### Materials

- **Type status:** Other material. **Occurrence:** catalogNumber: 11985; recordNumber: 16; recordedBy: K. Akpagana; lifeStage: Adult; reproductiveCondition: Fertile; **Taxon:** nameAccordingToID: The Plant List; scientificName: Doryopteris concolor var nicklesii (Tard.) Schelpe.; kingdom: Plantae; phylum: Monilophyta; class: Polypodiidae; order: Polypodiales; family: Pteridaceae; genus: Doryopteris; specificEpithet: concolor; infraspecificEpithet: nicklesii; taxonRank: variety; scientificNameAuthorship: (Tard.) Schelpe; **Location:** continent: Africa; country: Togo; stateProvince: Plateaux; decimalLatitude: 7.4333333; decimalLongitude: 1.4166667; geodeticDatum: WGS 1984; **Identification:** identifiedBy: K. Akpagana; dateIdentified: 27/5/1982; **Event:** eventDate: 27/5/1982; **Record Level:** institutionID: Herbarium togoense; collectionID: TOGO
- **Type status:** Other material. **Occurrence:** catalogNumber: 11986; recordNumber: 7720; recordedBy: J.-F. Brunel; lifeStage: Adult; reproductiveCondition: Fertile; **Taxon:** nameAccordingToID: The Plant List; scientificName: Doryopteris concolor var nicklesii (Tard.) Schelpe.; kingdom: Plantae; phylum: Monilophyta; class: Polypodiidae; order: Polypodiales; family: Pteridaceae; genus: Doryopteris; specificEpithet: concolor; infraspecificEpithet: nicklesii; taxonRank: variety; scientificNameAuthorship: (Tard.) Schelpe; **Location:** continent: Africa; country: Togo; stateProvince: Centrale; decimalLatitude: 8.05; decimalLongitude: 0.7833333; geodeticDatum: WGS 1984; **Identification:** identifiedBy: J.-F. Brunel; dateIdentified: /6/1982; **Event:** eventDate: /6/1982; **Record Level:** institutionID: Herbarium togoense; collectionID: TOGO
- **Type status:** Other material. **Occurrence:** catalogNumber: 11987; recordNumber: 7617; recordedBy: J.-F. Brunel; lifeStage: Adult; reproductiveCondition: Fertile; **Taxon:** nameAccordingToID: The Plant List; scientificName: Doryopteris concolor var nicklesii (Tard.) Schelpe.; kingdom: Plantae; phylum: Monilophyta; class: Polypodiidae; order: Polypodiales; family: Pteridaceae; genus: Doryopteris; specificEpithet: concolor; infraspecificEpithet: nicklesii; taxonRank: variety; scientificNameAuthorship: (Tard.) Schelpe; **Location:** continent: Africa; country: Togo; stateProvince: Plateaux; decimalLatitude: 7.4333333; decimalLongitude: 1.4166667; geodeticDatum: WGS 1984; **Identification:** identifiedBy: J.-F. Brunel; dateIdentified: /6/1982; **Event:** eventDate: /6/1982; **Record Level:** institutionID: Herbarium togoense; collectionID: TOGO

##### Ecological interactions

###### Native status

Native

##### Distribution

Togo (Ecological Zones 3 and 4), Zambia, Zimbabwe, Malawi, Tanzania, D.R.Congo, Madagascar, Ghana, Nigeria, Central African Republic

##### Notes

Doryopteris concolor var. nicklesii is a terrestrial fern, with closely spaced fronds, deltoids, measuring 10-35 cm long and 5-15 cm wide. The lamina is bipinnatifid. Pinnae measure 2-6 cm long and 0.7 to 3 cm wide. The petiole is covered by scattered scales. The sori, linear, protected by false indusia, are continuous at the edges of the leaf blade except at the bottom of sinus. Doryopteris concolor var. nicklesii usually grows on the banks of rivers in rainforest.

#### Doryopteris kirkii

(Hook.) Alston.

Doryopteris concolor var kirkii (Hook.) Wiss., *D. concolor* (Langsd. & Fisch.) Kuhn, *Cheilanthes kirkii* Hook, *C. argentea* sensu Peter F.D., *Pellaea geraniifolia* sensu Oliv, *Adiantum palmatum* Schumach. Beskr.

##### Materials

- **Type status:** Other material. **Occurrence:** catalogNumber: 11971; recordNumber: 1188; recordedBy: K. Akpagana; lifeStage: Adult; reproductiveCondition: Fertile; **Taxon:** nameAccordingToID: The Plant List; scientificName: *Doryopteris kirkii* (Hook.) Alston; kingdom: Plantae; phylum: Monilophyta; class: Polypodiidae; order: Polypodiales; family: Pteridaceae; genus: Doryopteris; specificEpithet: kirkii; taxonRank: species; scientificNameAuthorship: (Hook.) Alston.; **Location:** continent: Africa; country: Togo; stateProvince: Plateaux; decimalLatitude: 6.6666667; decimalLongitude: 1.1333333; geodeticDatum: WGS 1984; **Identification:** identifiedBy: K. Akpagana; dateIdentified: /11/1986; **Event:** eventDate: /11/1986; **Record Level:** institutionID: Herbarium togoense; collectionID: TOGO
- **Type status:** Other material. **Occurrence:** catalogNumber: 11972; recordNumber: 3992; recordedBy: Roussel; lifeStage: Adult; reproductiveCondition: Fertile; **Taxon:** nameAccordingToID: The Plant List; scientificName: *Doryopteris kirkii* (Hook.) Alston; kingdom: Plantae; phylum: Monilophyta; class: Polypodiidae; order: Polypodiales; family: Pteridaceae; genus: Doryopteris; specificEpithet: kirkii; taxonRank: species; scientificNameAuthorship: (Hook.) Alston.; **Location:** continent: Africa; country: Togo; stateProvince: Plateaux; decimalLatitude: 7.5333333; decimalLongitude: 0.9; geodeticDatum: WGS 1984; **Identification:** identifiedBy: Roussel; dateIdentified: 20/6/1988; **Event:** eventDate: 20/6/1988; **Record Level:** institutionID: Herbarium togoense; collectionID: TOGO
- **Type status:** Other material. **Occurrence:** catalogNumber: 11973; recordNumber: 10946; recordedBy: J.-F. Brunel; lifeStage: Adult; reproductiveCondition: Fertile; **Taxon:** nameAccordingToID: The Plant List; scientificName: *Doryopteris kirkii* (Hook.) Alston; kingdom: Plantae; phylum: Monilophyta; class: Polypodiidae; order: Polypodiales; family: Pteridaceae; genus: Doryopteris; specificEpithet: kirkii; taxonRank: species; scientificNameAuthorship: (Hook.) Alston.; **Location:** continent: Africa; country: Togo; stateProvince: Centrale; decimalLatitude: 8.05; decimalLongitude: 0.7833333; geodeticDatum: WGS 1984; **Identification:** identifiedBy: J.-F. Brunel; dateIdentified: /4/1987; **Event:** eventDate: /4/1987; **Record Level:** institutionID: Herbarium togoense; collectionID: TOGO
- **Type status:** Other material. **Occurrence:** catalogNumber: 11974; recordNumber: 1524; recordedBy: K. Akpagana; lifeStage: Adult; reproductiveCondition: Fertile; **Taxon:** nameAccordingToID: The Plant List; scientificName: *Doryopteris kirkii* (Hook.) Alston; kingdom: Plantae; phylum: Monilophyta; class: Polypodiidae; order: Polypodiales; family: Pteridaceae; genus: Doryopteris; specificEpithet: kirkii; taxonRank: species; scientificNameAuthorship: (Hook.) Alston.; **Location:** continent: Africa; country: Togo; stateProvince: Plateaux; decimalLatitude: 7; decimalLongitude: 0.75; geodeticDatum: WGS 1984; **Identification:** identifiedBy: K. Akpagana; dateIdentified: /3/1987; **Event:** eventDate: /3/1987; **Record Level:** institutionID: Herbarium togoense; collectionID: TOGO
- **Type status:** Other material. **Occurrence:** catalogNumber: 11975; recordNumber: 355bis; recordedBy: K. Akpagana, M. Kaman; lifeStage: Adult; reproductiveCondition: Fertile; **Taxon:** nameAccordingToID: The Plant List; scientificName: *Doryopteris kirkii* (Hook.) Alston; kingdom: Plantae; phylum: Monilophyta; class: Polypodiidae; order: Polypodiales; family: Pteridaceae; genus: Doryopteris; specificEpithet: kirkii; taxonRank: species; scientificNameAuthorship: (Hook.) Alston.; **Location:** continent: Africa; country: Togo; stateProvince: Plateaux; decimalLatitude: 7.5833333; decimalLongitude: 0.6; geodeticDatum: WGS 1984; **Identification:** identifiedBy: K. Akpagana; dateIdentified: 1984; **Event:** eventDate: 1984; **Record Level:** institutionID: Herbarium togoense; collectionID: TOGO
- **Type status:** Other material. **Occurrence:** catalogNumber: 11977; recordNumber: 1612; recordedBy: K. Akpagana; lifeStage: Adult; reproductiveCondition: Fertile; **Taxon:** nameAccordingToID: The Plant List; scientificName: *Doryopteris kirkii* (Hook.) Alston; kingdom: Plantae; phylum: Monilophyta; class: Polypodiidae; order: Polypodiales; family: Pteridaceae; genus: Doryopteris; specificEpithet: kirkii; taxonRank: species; scientificNameAuthorship: (Hook.) Alston.; **Location:** continent: Africa; country: Togo; stateProvince: Centrale; decimalLatitude: 8.05; decimalLongitude: 0.7833333; geodeticDatum: WGS 1984; **Identification:** identifiedBy: K. Akpagana; dateIdentified: /4/1987; **Event:** eventDate: /4/1987; **Record Level:** institutionID: Herbarium togoense; collectionID: TOGO
- **Type status:** Other material. **Occurrence:** catalogNumber: 11978; recordNumber: 527; recordedBy: K. Akpagana; lifeStage: Adult; reproductiveCondition: Fertile; **Taxon:** nameAccordingToID: The Plant List; scientificName: *Doryopteris kirkii* (Hook.) Alston; kingdom: Plantae; phylum: Monilophyta; class: Polypodiidae; order: Polypodiales; family: Pteridaceae; genus: Doryopteris; specificEpithet: kirkii; taxonRank: species; scientificNameAuthorship: (Hook.) Alston.; **Location:** continent: Africa; country: Togo; stateProvince: Plateaux; decimalLatitude: 6.6666667; decimalLongitude: 1.1333333; geodeticDatum: WGS 1984; **Identification:** identifiedBy: K. Akpagana; dateIdentified: /7/1986; **Event:** eventDate: /7/1986; **Record Level:** institutionID: Herbarium togoense; collectionID: TOGO
- **Type status:** Other material. **Occurrence:** catalogNumber: 11979; recordNumber: 1100; recordedBy: K. Akpagana; lifeStage: Adult; reproductiveCondition: Fertile; **Taxon:** nameAccordingToID: The Plant List; scientificName: *Doryopteris kirkii* (Hook.) Alston; kingdom: Plantae; phylum: Monilophyta; class: Polypodiidae; order: Polypodiales; family: Pteridaceae; genus: Doryopteris; specificEpithet: kirkii; taxonRank: species; scientificNameAuthorship: (Hook.) Alston.; **Location:** continent: Africa; country: Togo; stateProvince: Plateaux; decimalLatitude: 7.5833333; decimalLongitude: 0.6; geodeticDatum: WGS 1984; **Identification:** identifiedBy: K. Akpagana; dateIdentified: /11/1986; **Event:** eventDate: /11/1986; **Record Level:** institutionID: Herbarium togoense; collectionID: TOGO
- **Type status:** Other material. **Occurrence:** catalogNumber: 11981; recordNumber: 61; recordedBy: K. Akpagana; lifeStage: Adult; reproductiveCondition: Fertile; **Taxon:** nameAccordingToID: The Plant List; scientificName: *Doryopteris kirkii* (Hook.) Alston; kingdom: Plantae; phylum: Monilophyta; class: Polypodiidae; order: Polypodiales; family: Pteridaceae; genus: Doryopteris; specificEpithet: kirkii; taxonRank: species; scientificNameAuthorship: (Hook.) Alston.; **Location:** continent: Africa; country: Togo; stateProvince: Centrale; decimalLatitude: 8.1833333; decimalLongitude: 0.65; geodeticDatum: WGS 1984; **Identification:** identifiedBy: K. Akpagana; dateIdentified: /10/1982; **Event:** eventDate: /10/1982; **Record Level:** institutionID: Herbarium togoense; collectionID: TOGO
- **Type status:** Other material. **Occurrence:** catalogNumber: 11982; recordNumber: 22; recordedBy: K. Akpagana; lifeStage: Adult; reproductiveCondition: Fertile; **Taxon:** nameAccordingToID: The Plant List; scientificName: *Doryopteris kirkii* (Hook.) Alston; kingdom: Plantae; phylum: Monilophyta; class: Polypodiidae; order: Polypodiales; family: Pteridaceae; genus: Doryopteris; specificEpithet: kirkii; taxonRank: species; scientificNameAuthorship: (Hook.) Alston.; **Location:** continent: Africa; country: Togo; stateProvince: Centrale; decimalLatitude: 8.1833333; decimalLongitude: 0.65; geodeticDatum: WGS 1984; **Identification:** identifiedBy: K. Akpagana; dateIdentified: /6/1982; **Event:** eventDate: /6/1982; **Record Level:** institutionID: Herbarium togoense; collectionID: TOGO
- **Type status:** Other material. **Occurrence:** catalogNumber: 30047; recordNumber: 92; recordedBy: ABOTSI, SODJINOU & MINGOU; lifeStage: Adult; reproductiveCondition: Fertile; **Taxon:** nameAccordingToID: The Plant List; scientificName: *Doryopteris kirkii* (Hook.) Alston; kingdom: Plantae; phylum: Monilophyta; class: Polypodiidae; order: Polypodiales; family: Pteridaceae; genus: Doryopteris; specificEpithet: kirkii; taxonRank: species; scientificNameAuthorship: (Hook.) Alston.; **Location:** continent: Africa; country: Togo; stateProvince: Plateaux; decimalLatitude: 7.51394573; decimalLongitude: 0.6151256; geodeticDatum: WGS 1984; **Identification:** identifiedBy: K. E. Abotsi; dateIdentified: /05/2013; **Event:** eventDate: 04-04-13; **Record Level:** institutionID: Herbarium togoense; collectionID: TOGO
- **Type status:** Other material. **Occurrence:** catalogNumber: 30057; recordNumber: 127; recordedBy: ABOTSI, SODJINOU & MINGOU; **Taxon:** nameAccordingToID: The Plant List; scientificName: *Doryopteris kirkii* (Hook.) Alston; kingdom: Plantae; phylum: Monilophyta; class: Polypodiidae; order: Polypodiales; family: Pteridaceae; genus: Doryopteris; specificEpithet: kirkii; taxonRank: species; scientificNameAuthorship: (Hook.) Alston.; **Location:** continent: Africa; country: Togo; stateProvince: Plateaux; decimalLatitude: 8.17588129; decimalLongitude: 0.65987863; geodeticDatum: WGS 1984; **Identification:** identifiedBy: K. E. Abotsi; dateIdentified: /05/2013; **Event:** eventDate: 05-08-13; **Record Level:** institutionID: Herbarium togoense; collectionID: TOGO
- **Type status:** Other material. **Occurrence:** catalogNumber: 30061; recordNumber: 154; recordedBy: ABOTSI, SODJINOU & MINGOU; **Taxon:** nameAccordingToID: The Plant List; scientificName: *Doryopteris kirkii* (Hook.) Alston; kingdom: Plantae; phylum: Monilophyta; class: Polypodiidae; order: Polypodiales; family: Pteridaceae; genus: Doryopteris; specificEpithet: kirkii; taxonRank: species; scientificNameAuthorship: (Hook.) Alston.; **Location:** continent: Africa; country: Togo; stateProvince: Plateaux; decimalLatitude: 8.19615874; decimalLongitude: 0.61596339; geodeticDatum: WGS 1984; **Identification:** identifiedBy: K. E. Abotsi; dateIdentified: /05/2013; **Event:** eventDate: 05-09-13; **Record Level:** institutionID: Herbarium togoense; collectionID: TOGO
- **Type status:** Other material. **Occurrence:** catalogNumber: 30067; recordNumber: 166; recordedBy: ABOTSI, SODJINOU & MINGOU; **Taxon:** nameAccordingToID: The Plant List; scientificName: *Doryopteris kirkii* (Hook.) Alston; kingdom: Plantae; phylum: Monilophyta; class: Polypodiidae; order: Polypodiales; family: Pteridaceae; genus: Doryopteris; specificEpithet: kirkii; taxonRank: species; scientificNameAuthorship: (Hook.) Alston.; **Location:** continent: Africa; country: Togo; stateProvince: Plateaux; decimalLatitude: 8.01421749; decimalLongitude: 0.63662012; geodeticDatum: WGS 1984; **Identification:** identifiedBy: K. E. Abotsi; dateIdentified: /05/2013; **Event:** eventDate: 05-11-13; **Record Level:** institutionID: Herbarium togoense; collectionID: TOGO
- **Type status:** Other material. **Occurrence:** catalogNumber: 30068; recordNumber: 169; recordedBy: ABOTSI, SODJINOU & MINGOU; **Taxon:** nameAccordingToID: The Plant List; scientificName: *Doryopteris kirkii* (Hook.) Alston; kingdom: Plantae; phylum: Monilophyta; class: Polypodiidae; order: Polypodiales; family: Pteridaceae; genus: Doryopteris; specificEpithet: kirkii; taxonRank: species; scientificNameAuthorship: (Hook.) Alston.; **Location:** continent: Africa; country: Togo; stateProvince: Plateaux; decimalLatitude: 8.01361557; decimalLongitude: 0.63410289; geodeticDatum: WGS 1984; **Identification:** identifiedBy: K. E. Abotsi; dateIdentified: /05/2013; **Event:** eventDate: 05-11-13; **Record Level:** institutionID: Herbarium togoense; collectionID: TOGO
- **Type status:** Other material. **Occurrence:** catalogNumber: 30074; recordNumber: 196; recordedBy: ABOTSI, SODJINOU & MINGOU; **Taxon:** nameAccordingToID: The Plant List; scientificName: *Doryopteris kirkii* (Hook.) Alston; kingdom: Plantae; phylum: Monilophyta; class: Polypodiidae; order: Polypodiales; family: Pteridaceae; genus: Doryopteris; specificEpithet: kirkii; taxonRank: species; scientificNameAuthorship: (Hook.) Alston.; **Location:** continent: Africa; country: Togo; stateProvince: Plateaux; decimalLatitude: 8.01967741; decimalLongitude: 0.63048622; geodeticDatum: WGS 1984; **Identification:** identifiedBy: K. E. Abotsi; dateIdentified: /05/2013; **Event:** eventDate: 05-11-13; **Record Level:** institutionID: Herbarium togoense; collectionID: TOGO
- **Type status:** Other material. **Occurrence:** catalogNumber: 30075; recordNumber: 231; recordedBy: ABOTSI, SODJINOU & MINGOU; lifeStage: Adult; reproductiveCondition: Fertile; **Taxon:** nameAccordingToID: The Plant List; scientificName: *Doryopteris kirkii* (Hook.) Alston; kingdom: Plantae; phylum: Monilophyta; class: Polypodiidae; order: Polypodiales; family: Pteridaceae; genus: Doryopteris; specificEpithet: kirkii; taxonRank: species; scientificNameAuthorship: (Hook.) Alston.; **Location:** continent: Africa; country: Togo; stateProvince: Plateaux; decimalLatitude: 7.1264737; decimalLongitude: 0.65850971; geodeticDatum: WGS 1984; **Identification:** identifiedBy: K. E. Abotsi; dateIdentified: /05/2013; **Event:** eventDate: 04-09-13; **Record Level:** institutionID: Herbarium togoense; collectionID: TOGO
- **Type status:** Other material. **Occurrence:** catalogNumber: 30078; recordNumber: 256; recordedBy: ABOTSI, SODJINOU & MINGOU; **Taxon:** nameAccordingToID: The Plant List; scientificName: *Doryopteris kirkii* (Hook.) Alston; kingdom: Plantae; phylum: Monilophyta; class: Polypodiidae; order: Polypodiales; family: Pteridaceae; genus: Doryopteris; specificEpithet: kirkii; taxonRank: species; scientificNameAuthorship: (Hook.) Alston.; **Location:** continent: Africa; country: Togo; stateProvince: Plateaux; decimalLatitude: 7.23763756; decimalLongitude: 0.69907833; geodeticDatum: WGS 1984; **Identification:** identifiedBy: K. E. Abotsi; dateIdentified: /05/2013; **Event:** eventDate: 04-10-13; **Record Level:** institutionID: Herbarium togoense; collectionID: TOGO
- **Type status:** Other material. **Occurrence:** catalogNumber: 30081; recordNumber: 272; recordedBy: ABOTSI, SODJINOU & MINGOU; **Taxon:** nameAccordingToID: The Plant List; scientificName: *Doryopteris kirkii* (Hook.) Alston; kingdom: Plantae; phylum: Monilophyta; class: Polypodiidae; order: Polypodiales; family: Pteridaceae; genus: Doryopteris; specificEpithet: kirkii; taxonRank: species; scientificNameAuthorship: (Hook.) Alston.; **Location:** continent: Africa; country: Togo; stateProvince: Plateaux; decimalLatitude: 7.10634408; decimalLongitude: 0.60880081; geodeticDatum: WGS 1984; **Identification:** identifiedBy: K. E. Abotsi; dateIdentified: /05/2013; **Event:** eventDate: 04-11-13; **Record Level:** institutionID: Herbarium togoense; collectionID: TOGO
- **Type status:** Other material. **Occurrence:** catalogNumber: 30083; recordNumber: 274; recordedBy: ABOTSI, SODJINOU & MINGOU; **Taxon:** nameAccordingToID: The Plant List; scientificName: *Doryopteris kirkii* (Hook.) Alston; kingdom: Plantae; phylum: Monilophyta; class: Polypodiidae; order: Polypodiales; family: Pteridaceae; genus: Doryopteris; specificEpithet: kirkii; taxonRank: species; scientificNameAuthorship: (Hook.) Alston.; **Location:** continent: Africa; country: Togo; stateProvince: Plateaux; decimalLatitude: 7.1058468; decimalLongitude: 0.60876719; geodeticDatum: WGS 1984; **Identification:** identifiedBy: K. E. Abotsi; dateIdentified: /05/2013; **Event:** eventDate: 04-11-13; **Record Level:** institutionID: Herbarium togoense; collectionID: TOGO
- **Type status:** Other material. **Occurrence:** catalogNumber: 30085; recordNumber: 283; recordedBy: ABOTSI, SODJINOU & MINGOU; **Taxon:** nameAccordingToID: The Plant List; scientificName: *Doryopteris kirkii* (Hook.) Alston; kingdom: Plantae; phylum: Monilophyta; class: Polypodiidae; order: Polypodiales; family: Pteridaceae; genus: Doryopteris; specificEpithet: kirkii; taxonRank: species; scientificNameAuthorship: (Hook.) Alston.; **Location:** continent: Africa; country: Togo; stateProvince: Plateaux; decimalLatitude: 6.94840325; decimalLongitude: 0.57909955; geodeticDatum: WGS 1984; **Identification:** identifiedBy: K. E. Abotsi; dateIdentified: /05/2013; **Event:** eventDate: 04-15-13; **Record Level:** institutionID: Herbarium togoense; collectionID: TOGO
- **Type status:** Other material. **Occurrence:** catalogNumber: 30086; recordNumber: 284; recordedBy: ABOTSI, SODJINOU & MINGOU; **Taxon:** nameAccordingToID: The Plant List; scientificName: *Doryopteris kirkii* (Hook.) Alston; kingdom: Plantae; phylum: Monilophyta; class: Polypodiidae; order: Polypodiales; family: Pteridaceae; genus: Doryopteris; specificEpithet: kirkii; taxonRank: species; scientificNameAuthorship: (Hook.) Alston.; **Location:** continent: Africa; country: Togo; stateProvince: Plateaux; decimalLatitude: 6.94840325; decimalLongitude: 0.57909955; geodeticDatum: WGS 1984; **Identification:** identifiedBy: K. E. Abotsi; dateIdentified: /05/2013; **Event:** eventDate: 04-15-13; **Record Level:** institutionID: Herbarium togoense; collectionID: TOGO
- **Type status:** Other material. **Occurrence:** catalogNumber: 30088; recordNumber: 289; recordedBy: ABOTSI, SODJINOU & MINGOU; **Taxon:** nameAccordingToID: The Plant List; scientificName: *Doryopteris kirkii* (Hook.) Alston; kingdom: Plantae; phylum: Monilophyta; class: Polypodiidae; order: Polypodiales; family: Pteridaceae; genus: Doryopteris; specificEpithet: kirkii; taxonRank: species; scientificNameAuthorship: (Hook.) Alston.; **Location:** continent: Africa; country: Togo; stateProvince: Plateaux; decimalLatitude: 6.95458625; decimalLongitude: 0.58282158; geodeticDatum: WGS 1984; **Identification:** identifiedBy: K. E. Abotsi; dateIdentified: /05/2013; **Event:** eventDate: 04-15-13; **Record Level:** institutionID: Herbarium togoense; collectionID: TOGO
- **Type status:** Other material. **Occurrence:** catalogNumber: 30092; recordNumber: 295; recordedBy: ABOTSI, SODJINOU & MINGOU; **Taxon:** nameAccordingToID: The Plant List; scientificName: *Doryopteris kirkii* (Hook.) Alston; kingdom: Plantae; phylum: Monilophyta; class: Polypodiidae; order: Polypodiales; family: Pteridaceae; genus: Doryopteris; specificEpithet: kirkii; taxonRank: species; scientificNameAuthorship: (Hook.) Alston.; **Location:** continent: Africa; country: Togo; stateProvince: Plateaux; decimalLatitude: 6.9544104; decimalLongitude: 0.58024464; geodeticDatum: WGS 1984; **Identification:** identifiedBy: K. E. Abotsi; dateIdentified: /05/2013; **Event:** eventDate: 04-15-13; **Record Level:** institutionID: Herbarium togoense; collectionID: TOGO

##### Ecological interactions

###### Native status

Native

##### Distribution

Togo (Ecological Zone 4), South Africa, Namibia, Swaziland, Botswana, Zambia, Zimbabwe, Malawi, Mozambique, Angola, Cameroon, Central African Republic, Liberia, Tanzania, Uganda, Madagascar, Comores, Guinea, Ivory Coast, Ghana, Nigeria, Socotra, Ethiopia, S-India, Sri Lanka, Philippines, Vietnam, Sulawesi, Fiji, Society Isl., Tonga, New Caledonia, Western Samoa, American Samoa, Marquesas Isl., Austral Isl., Australia, Taiwan, China

##### Notes

*Doryopteris kirkii* has a short rhizome, closely spaced fronds, wearing very thickened scales in the center and very thin at the edges (Fig. 11a, b). There is a dimorphism within fronds. The petiole of sterile fronds is black, 15 to 20 cm long, scaly at the extreme base, channeled. The sterile blade is 7 to 10 cm long as wide. It is deltoid, bipinnatifid, all pinnae are opposite and connected by a large wing. The lower pinnae are sickle-shaped, about 6-7 cm long, very developed basiscopically, divided down to costa in acute pinnules, also lobed. The texture of the leaf blade is sub-coriaceous and its surface is glabrous. The fertile fronds have a slightly longer stalk than the sterile fronds, with the same shape, but more deeply lobed. The rachis and midrib are black and hairless. The sori are linear, marginal, interrupted and slightly covered by a pale and narrow lobe of the lamina, as a false indusium (Fig. 11c, d). The plant usually grows on rocks, cliffs, banks of rivers and roadsides in forest areas.

#### Haplopteris guineensisvar.guineensis

(Desv.) Crane

Vittaria guineensis Desv, *V. congoensis* H.Christ, V. guineensis var. cancellata Hieron. *Pteris guineensis* (Desv) Desv

##### Materials

- **Type status:** Other material. **Occurrence:** catalogNumber: 12466; recordNumber: 8174; recordedBy: J.-F. Brunel; lifeStage: Adult; reproductiveCondition: Fertile; **Taxon:** nameAccordingToID: The Plant List; scientificName: Haplopteris guineensis (Desv.) Crane var. guineensis; kingdom: Plantae; phylum: Monilophyta; class: Polypodiidae; order: Polypodiales; family: Pteridaceae; genus: Haplopteris; specificEpithet: guineensis; infraspecificEpithet: guineensis; taxonRank: variety; scientificNameAuthorship: (Desv.) Crane; **Location:** continent: Africa; country: Togo; stateProvince: Plateaux; decimalLatitude: 7.5833333; decimalLongitude: 0.6; geodeticDatum: WGS 1984; **Identification:** identifiedBy: J.-F. Brunel; dateIdentified: /1/1984; **Event:** eventDate: /1/1984; **Record Level:** institutionID: Herbarium togoense; collectionID: TOGO
- **Type status:** Other material. **Occurrence:** catalogNumber: 12467; recordNumber: 5592; recordedBy: J.-F. Brunel; lifeStage: Adult; reproductiveCondition: Fertile; **Taxon:** nameAccordingToID: The Plant List; scientificName: Haplopteris guineensis (Desv.) Crane var. guineensis; kingdom: Plantae; phylum: Monilophyta; class: Polypodiidae; order: Polypodiales; family: Pteridaceae; genus: Haplopteris; specificEpithet: guineensis; infraspecificEpithet: guineensis; taxonRank: variety; scientificNameAuthorship: (Desv.) Crane; **Location:** continent: Africa; country: Togo; stateProvince: Plateaux; decimalLatitude: 7; decimalLongitude: 0.75; geodeticDatum: WGS 1984; **Identification:** identifiedBy: J.-F. Brunel; **Record Level:** institutionID: Herbarium togoense; collectionID: TOGO

##### Ecological interactions

###### Native status

Native

##### Distribution

Togo (Ecological Zone 4), Congo, Guinea, Sao Tomé, Principe Isl., Bioko Isl., Sierra Leone, Liberia, Ivory Coast, Ghana, Nigeria, Cameroon, Central African Republic, Equatorial Guinea, Gabon, Congo, Angola, Uganda

##### Notes

Haplopteris guineensis var. guineensis is a small epiphytic fern, with a shortly creeping rhizome covered by scales, black and lanceolate, darker in the center, with a colorless light. The frond is linear-lanceolate, 10 to 60 cm long and 0.4 to 1.2 cm width. The stipe is 2-5 cm long, blackish at the base. The end of the leaf blade is acute, mucronnate, with terminal hydathode. Midrib only appears only at the base of the frond. The lateral ribs are not visible, except for the young fronds. The sori are marginal, immersed. The plant usually grows on the trunks of trees in the gallery forests and rainforests.

#### Pellaea dura

(Willd.) Hook.

Pellaea doniana Hook, *Pteris doniana* (Hook.) Kuhn, *Pteridella doniana* (Hook.) Kuhn.

##### Materials

- **Type status:** Other material. **Occurrence:** catalogNumber: 12027; recordNumber: 131; recordedBy: K. Akpagana; lifeStage: Adult; reproductiveCondition: Fertile; **Taxon:** nameAccordingToID: The Plant List; scientificName: *Pellaea dura* (Willd.) Hook.; kingdom: Plantae; phylum: Monilophyta; class: Polypodiidae; order: Polypodiales; family: Pteridaceae; genus: Pellaea; specificEpithet: dura; taxonRank: species; scientificNameAuthorship: (Willd.) Hook.; **Location:** continent: Africa; country: Togo; stateProvince: Plateaux; decimalLatitude: 7.0166667; decimalLongitude: 0.65; geodeticDatum: WGS 1984; **Identification:** identifiedBy: K. Akpagana; dateIdentified: 1984; **Event:** eventDate: 1984; **Record Level:** institutionID: Herbarium togoense; collectionID: TOGO
- **Type status:** Other material. **Occurrence:** catalogNumber: 12028; recordNumber: 7743; recordedBy: J.-F. Brunel; lifeStage: Adult; reproductiveCondition: Fertile; **Taxon:** nameAccordingToID: The Plant List; scientificName: *Pellaea dura* (Willd.) Hook.; kingdom: Plantae; phylum: Monilophyta; class: Polypodiidae; order: Polypodiales; family: Pteridaceae; genus: Pellaea; specificEpithet: dura; taxonRank: species; scientificNameAuthorship: (Willd.) Hook.; **Location:** continent: Africa; country: Togo; stateProvince: Centrale; decimalLatitude: 8.05; decimalLongitude: 0.7833333; geodeticDatum: WGS 1984; **Identification:** identifiedBy: J.-F. Brunel; dateIdentified: /6/1982; **Event:** eventDate: /6/1982; **Record Level:** institutionID: Herbarium togoense; collectionID: TOGO
- **Type status:** Other material. **Occurrence:** catalogNumber: 12030; recordNumber: 533bis; recordedBy: K. Akpagana; lifeStage: Adult; reproductiveCondition: Fertile; **Taxon:** nameAccordingToID: The Plant List; scientificName: *Pellaea dura* (Willd.) Hook.; kingdom: Plantae; phylum: Monilophyta; class: Polypodiidae; order: Polypodiales; family: Pteridaceae; genus: Pellaea; specificEpithet: dura; taxonRank: species; scientificNameAuthorship: (Willd.) Hook.; **Location:** continent: Africa; country: Togo; stateProvince: Plateaux; decimalLatitude: 6.6666667; decimalLongitude: 1.1333333; geodeticDatum: WGS 1984; **Identification:** identifiedBy: K. Akpagana; dateIdentified: /7/1986; **Event:** eventDate: /7/1986; **Record Level:** institutionID: Herbarium togoense; collectionID: TOGO
- **Type status:** Other material. **Occurrence:** catalogNumber: 12031; recordNumber: 26; recordedBy: A.K. Guelly; lifeStage: Adult; reproductiveCondition: Fertile; **Taxon:** nameAccordingToID: The Plant List; scientificName: *Pellaea dura* (Willd.) Hook.; kingdom: Plantae; phylum: Monilophyta; class: Polypodiidae; order: Polypodiales; family: Pteridaceae; genus: Pellaea; specificEpithet: dura; taxonRank: species; scientificNameAuthorship: (Willd.) Hook.; **Location:** continent: Africa; country: Togo; stateProvince: Plateaux; decimalLatitude: 7; decimalLongitude: 0.75; geodeticDatum: WGS 1984; **Identification:** identifiedBy: A.K. Guelly; dateIdentified: 24/1/1984; **Event:** eventDate: 24/1/1984; **Record Level:** institutionID: Herbarium togoense; collectionID: TOGO
- **Type status:** Other material. **Occurrence:** catalogNumber: 12032; recordNumber: 101bis; recordedBy: J.-F. Brunel; lifeStage: Adult; reproductiveCondition: Fertile; **Taxon:** nameAccordingToID: The Plant List; scientificName: *Pellaea dura* (Willd.) Hook.; kingdom: Plantae; phylum: Monilophyta; class: Polypodiidae; order: Polypodiales; family: Pteridaceae; genus: Pellaea; specificEpithet: dura; taxonRank: species; scientificNameAuthorship: (Willd.) Hook.; **Location:** continent: Africa; country: Togo; stateProvince: Plateaux; decimalLatitude: 7.0166667; decimalLongitude: 0.65; geodeticDatum: WGS 1984; **Identification:** identifiedBy: C.A. Meyer; dateIdentified: 1980; **Event:** eventDate: 1973; **Record Level:** institutionID: Herbarium togoense; collectionID: TOGO
- **Type status:** Other material. **Occurrence:** catalogNumber: 12035; recordNumber: s.n.; recordedBy: Ayéna; lifeStage: Adult; reproductiveCondition: Fertile; **Taxon:** nameAccordingToID: The Plant List; scientificName: *Pellaea dura* (Willd.) Hook.; kingdom: Plantae; phylum: Monilophyta; class: Polypodiidae; order: Polypodiales; family: Pteridaceae; genus: Pellaea; specificEpithet: dura; taxonRank: species; scientificNameAuthorship: (Willd.) Hook.; **Location:** continent: Africa; country: Togo; stateProvince: Plateaux; decimalLatitude: 7.0166667; decimalLongitude: 0.65; geodeticDatum: WGS 1984; **Identification:** identifiedBy: J.-F. Brunel; dateIdentified: 24/2/1984; **Event:** eventDate: 24/2/1984; **Record Level:** institutionID: Herbarium togoense; collectionID: TOGO
- **Type status:** Other material. **Occurrence:** catalogNumber: 12037; recordNumber: 8184; recordedBy: J.-F. Brunel; lifeStage: Adult; reproductiveCondition: Fertile; **Taxon:** nameAccordingToID: The Plant List; scientificName: *Pellaea dura* (Willd.) Hook.; kingdom: Plantae; phylum: Monilophyta; class: Polypodiidae; order: Polypodiales; family: Pteridaceae; genus: Pellaea; specificEpithet: dura; taxonRank: species; scientificNameAuthorship: (Willd.) Hook.; **Location:** continent: Africa; country: Togo; stateProvince: Plateaux; decimalLatitude: 6.6666667; decimalLongitude: 1.1333333; geodeticDatum: WGS 1984; **Identification:** identifiedBy: J.-F. Brunel; dateIdentified: /1/1984; **Event:** eventDate: /1/1984; **Record Level:** institutionID: Herbarium togoense; collectionID: TOGO
- **Type status:** Other material. **Occurrence:** catalogNumber: 12063; recordNumber: 1043; recordedBy: K. Akpagana; lifeStage: Adult; reproductiveCondition: Fertile; **Taxon:** nameAccordingToID: The Plant List; scientificName: *Pellaea dura* (Willd.) Hook.; kingdom: Plantae; phylum: Monilophyta; class: Polypodiidae; order: Polypodiales; family: Pteridaceae; genus: Pellaea; specificEpithet: dura; taxonRank: species; scientificNameAuthorship: (Willd.) Hook.; **Location:** continent: Africa; country: Togo; stateProvince: Centrale; decimalLatitude: 8.2166667; decimalLongitude: 0.8833333; geodeticDatum: WGS 1984; **Identification:** identifiedBy: K. Akpagana; dateIdentified: /10/1986; **Event:** eventDate: /10/1986; **Record Level:** institutionID: Herbarium togoense; collectionID: TOGO
- **Type status:** Other material. **Occurrence:** catalogNumber: 30041; recordNumber: 61; recordedBy: ABOTSI, SODJINOU & MINGOU; **Taxon:** nameAccordingToID: The Plant List; scientificName: *Pellaea dura* (Willd.) Hook.; kingdom: Plantae; phylum: Monilophyta; class: Polypodiidae; order: Polypodiales; family: Pteridaceae; genus: Pellaea; specificEpithet: dura; taxonRank: species; scientificNameAuthorship: (Willd.) Hook.; **Location:** continent: Africa; country: Togo; stateProvince: Plateaux; decimalLatitude: 8.17456938; decimalLongitude: 0.65808114; geodeticDatum: WGS 1984; **Identification:** identifiedBy: K. E. Abotsi; dateIdentified: /05/2013; **Event:** eventDate: 05-08-13; **Record Level:** institutionID: Herbarium togoense; collectionID: TOGO
- **Type status:** Other material. **Occurrence:** catalogNumber: 30050; recordNumber: 97; recordedBy: ABOTSI, SODJINOU & MINGOU; **Taxon:** nameAccordingToID: The Plant List; scientificName: *Pellaea dura* (Willd.) Hook.; kingdom: Plantae; phylum: Monilophyta; class: Polypodiidae; order: Polypodiales; family: Pteridaceae; genus: Pellaea; specificEpithet: dura; taxonRank: species; scientificNameAuthorship: (Willd.) Hook.; **Location:** continent: Africa; country: Togo; stateProvince: Plateaux; decimalLatitude: 7.51536397; decimalLongitude: 0.61998118; geodeticDatum: WGS 1984; **Identification:** identifiedBy: K. E. Abotsi; dateIdentified: /05/2013; **Event:** eventDate: 04-04-13; **Record Level:** institutionID: Herbarium togoense; collectionID: TOGO
- **Type status:** Other material. **Occurrence:** catalogNumber: 30066; recordNumber: 165; recordedBy: ABOTSI, SODJINOU & MINGOU; **Taxon:** nameAccordingToID: The Plant List; scientificName: *Pellaea dura* (Willd.) Hook.; kingdom: Plantae; phylum: Monilophyta; class: Polypodiidae; order: Polypodiales; family: Pteridaceae; genus: Pellaea; specificEpithet: dura; taxonRank: species; scientificNameAuthorship: (Willd.) Hook.; **Location:** continent: Africa; country: Togo; stateProvince: Plateaux; decimalLatitude: 8.01451189; decimalLongitude: 0.63594744; geodeticDatum: WGS 1984; **Identification:** identifiedBy: K. E. Abotsi; dateIdentified: /05/2013; **Event:** eventDate: 05-11-13; **Record Level:** institutionID: Herbarium togoense; collectionID: TOGO
- **Type status:** Other material. **Occurrence:** catalogNumber: 30071; recordNumber: 178; recordedBy: ABOTSI, SODJINOU & MINGOU; **Taxon:** nameAccordingToID: The Plant List; scientificName: *Pellaea dura* (Willd.) Hook.; kingdom: Plantae; phylum: Monilophyta; class: Polypodiidae; order: Polypodiales; family: Pteridaceae; genus: Pellaea; specificEpithet: dura; taxonRank: species; scientificNameAuthorship: (Willd.) Hook.; **Location:** continent: Africa; country: Togo; stateProvince: Plateaux; decimalLatitude: 8.01589182; decimalLongitude: 0.6322944; geodeticDatum: WGS 1984; **Identification:** identifiedBy: K. E. Abotsi; dateIdentified: /05/2013; **Event:** eventDate: 05-11-13; **Record Level:** institutionID: Herbarium togoense; collectionID: TOGO
- **Type status:** Other material. **Occurrence:** catalogNumber: 30079; recordNumber: 270; recordedBy: ABOTSI, SODJINOU & MINGOU; **Taxon:** nameAccordingToID: The Plant List; scientificName: *Pellaea dura* (Willd.) Hook.; kingdom: Plantae; phylum: Monilophyta; class: Polypodiidae; order: Polypodiales; family: Pteridaceae; genus: Pellaea; specificEpithet: dura; taxonRank: species; scientificNameAuthorship: (Willd.) Hook.; **Location:** continent: Africa; country: Togo; stateProvince: Plateaux; decimalLatitude: 7.11281536; decimalLongitude: 0.61053174; geodeticDatum: WGS 1984; **Identification:** identifiedBy: K. E. Abotsi; dateIdentified: /05/2013; **Event:** eventDate: 04-11-13; **Record Level:** institutionID: Herbarium togoense; collectionID: TOGO
- **Type status:** Other material. **Occurrence:** catalogNumber: 30093; recordNumber: 298; recordedBy: ABOTSI, SODJINOU & MINGOU; **Taxon:** nameAccordingToID: The Plant List; scientificName: *Pellaea dura* (Willd.) Hook.; kingdom: Plantae; phylum: Monilophyta; class: Polypodiidae; order: Polypodiales; family: Pteridaceae; genus: Pellaea; specificEpithet: dura; taxonRank: species; scientificNameAuthorship: (Willd.) Hook.; **Location:** continent: Africa; country: Togo; stateProvince: Plateaux; decimalLatitude: 6.94781003; decimalLongitude: 0.57974477; geodeticDatum: WGS 1984; **Identification:** identifiedBy: K. E. Abotsi; dateIdentified: /05/2013; **Event:** eventDate: 04-15-13; **Record Level:** institutionID: Herbarium togoense; collectionID: TOGO

##### Ecological interactions

###### Native status

Native

##### Distribution

Togo (Ecological Zones 3 and 4), Burundi, Ghana, Malawi, Tanzania, Uganda, Zambia, Kenya, Mozambique, Seychelles, Principe Isl., Sao Tomé, Chad, Senegal, Guinea, Sierra Leone, Liberia, Ivory Coast, Benin, Bioko Isl., Nigeria, Cameroon, Central African Republic, Annobon Isl., Congo, D.R.Congo, Angola, Mali, Sudan

##### Notes

*Pellaea dura* is a fern with erect rhizome, with fronds in clumps, from 19 to 60 (-100) cm long, with narrow scales, pale and colorless to light (Fig. 12a, bFig. 12a). The stipe is black, brown or purple, 5-39cm long, covered at the base with linear scales. The lamina is oblong to oblong-lanceolate, 15-58 cm long and 8-18 cm wide, pinnate. There are 6 to 21 pairs of lateral pinnae, oblong-lanceolate, oblong or oblong-oval, 6-10 cm long, 1-1.5 cm wide, stalked, base rounded or slightly cordate (Fig. 12c). The leaf blade is smooth and has a leathery texture. Midrib is almost black in its lower part. The veins are usually invisible. The indusium is usually hidden by the sori (Fig. 12d). The plant usually grows on the banks of rivers, roadsides, cliffs and rocks in the forest.

#### Pityrogramma calomelanosvar.calomelanos

(L.) Link.

Acrostichum calomelanos . L., *A. ebeneum* L, *Gymnogramma calomelanos* (Link) Kaulf., *G. distans* Link., *Ceropteris calomelanos* (L) Underw., *Pityrogramma chamaesorus* Domin., P. insularis Domin.

##### Materials

- **Type status:** Other material. **Occurrence:** catalogNumber: 12039; recordNumber: 4053; recordedBy: Roussel; lifeStage: Adult; reproductiveCondition: Fertile; **Taxon:** nameAccordingToID: The Plant List; scientificName: Pityrogramma calomelanos (L.) Link var. calomelanos; kingdom: Plantae; phylum: Monilophyta; class: Polypodiidae; order: Polypodiales; family: Pteridaceae; genus: Pityrogramma; specificEpithet: calomelanos; infraspecificEpithet: calomelanos; taxonRank: variety; scientificNameAuthorship: (L.) Link.; **Location:** continent: Africa; country: Togo; stateProvince: Savanes; decimalLatitude: 9.25; decimalLongitude: 0.7833333; geodeticDatum: WGS 1984; **Identification:** identifiedBy: Roussel; dateIdentified: 7/9/1988; **Event:** eventDate: 7/9/1988; **Record Level:** institutionID: Herbarium togoense; collectionID: TOGO
- **Type status:** Other material. **Occurrence:** catalogNumber: 12040; recordNumber: 130; recordedBy: K. Akpagana; lifeStage: Adult; reproductiveCondition: Fertile; **Taxon:** nameAccordingToID: The Plant List; scientificName: Pityrogramma calomelanos (L.) Link var. calomelanos; kingdom: Plantae; phylum: Monilophyta; class: Polypodiidae; order: Polypodiales; family: Pteridaceae; genus: Pityrogramma; specificEpithet: calomelanos; infraspecificEpithet: calomelanos; taxonRank: variety; scientificNameAuthorship: (L.) Link.; **Location:** continent: Africa; country: Togo; stateProvince: Plateaux; decimalLatitude: 7.0166667; decimalLongitude: 0.65; geodeticDatum: WGS 1984; **Identification:** identifiedBy: K. Akpagana; dateIdentified: /3/1984; **Event:** eventDate: /3/1984; **Record Level:** institutionID: Herbarium togoense; collectionID: TOGO
- **Type status:** Other material. **Occurrence:** catalogNumber: 12041; recordNumber: 4876; recordedBy: J.-F. Brunel; lifeStage: Adult; reproductiveCondition: Fertile; **Taxon:** nameAccordingToID: The Plant List; scientificName: Pityrogramma calomelanos (L.) Link var. calomelanos; kingdom: Plantae; phylum: Monilophyta; class: Polypodiidae; order: Polypodiales; family: Pteridaceae; genus: Pityrogramma; specificEpithet: calomelanos; infraspecificEpithet: calomelanos; taxonRank: variety; scientificNameAuthorship: (L.) Link.; **Location:** continent: Africa; country: Togo; stateProvince: Plateaux; decimalLatitude: 7; decimalLongitude: 0.75; geodeticDatum: WGS 1984; **Identification:** identifiedBy: J.-F. Brunel; **Record Level:** institutionID: Herbarium togoense; collectionID: TOGO
- **Type status:** Other material. **Occurrence:** catalogNumber: 12042; recordNumber: 514; recordedBy: K. Akpagana; lifeStage: Adult; reproductiveCondition: Fertile; **Taxon:** nameAccordingToID: The Plant List; scientificName: Pityrogramma calomelanos (L.) Link var. calomelanos; kingdom: Plantae; phylum: Monilophyta; class: Polypodiidae; order: Polypodiales; family: Pteridaceae; genus: Pityrogramma; specificEpithet: calomelanos; infraspecificEpithet: calomelanos; taxonRank: variety; scientificNameAuthorship: (L.) Link.; **Location:** continent: Africa; country: Togo; stateProvince: Plateaux; decimalLatitude: 7.5833333; decimalLongitude: 0.6; geodeticDatum: WGS 1984; **Identification:** identifiedBy: K. Akpagana; dateIdentified: /7/1986; **Event:** eventDate: /7/1986; **Record Level:** institutionID: Herbarium togoense; collectionID: TOGO
- **Type status:** Other material. **Occurrence:** catalogNumber: 12044; recordNumber: 4375; recordedBy: J.-F. Brunel; lifeStage: Adult; reproductiveCondition: Fertile; **Taxon:** nameAccordingToID: The Plant List; scientificName: Pityrogramma calomelanos (L.) Link var. calomelanos; kingdom: Plantae; phylum: Monilophyta; class: Polypodiidae; order: Polypodiales; family: Pteridaceae; genus: Pityrogramma; specificEpithet: calomelanos; infraspecificEpithet: calomelanos; taxonRank: variety; scientificNameAuthorship: (L.) Link.; **Location:** continent: Africa; country: Togo; stateProvince: Centrale; decimalLatitude: 9.25; decimalLongitude: 1.2; geodeticDatum: WGS 1984; **Identification:** identifiedBy: J.-F. Brunel; dateIdentified: /4/1977; **Event:** eventDate: /4/1977; **Record Level:** institutionID: Herbarium togoense; collectionID: TOGO
- **Type status:** Other material. **Occurrence:** catalogNumber: 12045; recordNumber: 7302; recordedBy: J.-F. Brunel; lifeStage: Adult; reproductiveCondition: Fertile; **Taxon:** nameAccordingToID: The Plant List; scientificName: Pityrogramma calomelanos (L.) Link var. calomelanos; kingdom: Plantae; phylum: Monilophyta; class: Polypodiidae; order: Polypodiales; family: Pteridaceae; genus: Pityrogramma; specificEpithet: calomelanos; infraspecificEpithet: calomelanos; taxonRank: variety; scientificNameAuthorship: (L.) Link.; **Location:** continent: Africa; country: Togo; stateProvince: Plateaux; decimalLatitude: 7.5833333; decimalLongitude: 0.6; geodeticDatum: WGS 1984; **Identification:** identifiedBy: J.-F. Brunel; dateIdentified: /12/1980; **Event:** eventDate: /12/1980; **Record Level:** institutionID: Herbarium togoense; collectionID: TOGO
- **Type status:** Other material. **Occurrence:** catalogNumber: 12046; recordNumber: 27; recordedBy: A.K. Guelly; lifeStage: Adult; reproductiveCondition: Fertile; **Taxon:** nameAccordingToID: The Plant List; scientificName: Pityrogramma calomelanos (L.) Link var. calomelanos; kingdom: Plantae; phylum: Monilophyta; class: Polypodiidae; order: Polypodiales; family: Pteridaceae; genus: Pityrogramma; specificEpithet: calomelanos; infraspecificEpithet: calomelanos; taxonRank: variety; scientificNameAuthorship: (L.) Link.; **Location:** continent: Africa; country: Togo; stateProvince: Plateaux; decimalLatitude: 7.0166667; decimalLongitude: 0.65; geodeticDatum: WGS 1984; **Identification:** identifiedBy: A.K. Guelly; dateIdentified: 24/3/1984; **Event:** eventDate: 24/3/1984; **Record Level:** institutionID: Herbarium togoense; collectionID: TOGO
- **Type status:** Other material. **Occurrence:** catalogNumber: 12047; recordNumber: 7854; recordedBy: J.-F. Brunel; lifeStage: Adult; reproductiveCondition: Fertile; **Taxon:** nameAccordingToID: The Plant List; scientificName: Pityrogramma calomelanos (L.) Link var. calomelanos; kingdom: Plantae; phylum: Monilophyta; class: Polypodiidae; order: Polypodiales; family: Pteridaceae; genus: Pityrogramma; specificEpithet: calomelanos; infraspecificEpithet: calomelanos; taxonRank: variety; scientificNameAuthorship: (L.) Link.; **Location:** continent: Africa; country: Togo; stateProvince: Kara; decimalLatitude: 9.95; decimalLongitude: 1.05; geodeticDatum: WGS 1984; **Identification:** identifiedBy: J.-F. Brunel; dateIdentified: /1/1983; **Event:** eventDate: /1/1983; **Record Level:** institutionID: Herbarium togoense; collectionID: TOGO
- **Type status:** Other material. **Occurrence:** catalogNumber: 12048; recordNumber: 10796; recordedBy: J.-F. Brunel; lifeStage: Adult; reproductiveCondition: Fertile; **Taxon:** nameAccordingToID: The Plant List; scientificName: Pityrogramma calomelanos (L.) Link var. calomelanos; kingdom: Plantae; phylum: Monilophyta; class: Polypodiidae; order: Polypodiales; family: Pteridaceae; genus: Pityrogramma; specificEpithet: calomelanos; infraspecificEpithet: calomelanos; taxonRank: variety; scientificNameAuthorship: (L.) Link.; **Location:** continent: Africa; country: Togo; stateProvince: Kara; decimalLatitude: 9.3833333; decimalLongitude: 0.85; geodeticDatum: WGS 1984; **Identification:** identifiedBy: J.-F. Brunel; dateIdentified: /3/1987; **Event:** eventDate: /3/1987; **Record Level:** institutionID: Herbarium togoense; collectionID: TOGO
- **Type status:** Other material. **Occurrence:** catalogNumber: 12049; recordNumber: 7703; recordedBy: P.A. Schäfer; lifeStage: Adult; reproductiveCondition: Fertile; **Taxon:** nameAccordingToID: The Plant List; scientificName: Pityrogramma calomelanos (L.) Link var. calomelanos; kingdom: Plantae; phylum: Monilophyta; class: Polypodiidae; order: Polypodiales; family: Pteridaceae; genus: Pityrogramma; specificEpithet: calomelanos; infraspecificEpithet: calomelanos; taxonRank: variety; scientificNameAuthorship: (L.) Link.; **Location:** continent: Africa; country: Togo; stateProvince: Kara; decimalLatitude: 9.1666667; decimalLongitude: 0.95; geodeticDatum: WGS 1984; **Identification:** identifiedBy: P.A. Schäfer; dateIdentified: 20/12/1982; **Event:** eventDate: 20/12/1982; **Record Level:** institutionID: Herbarium togoense; collectionID: TOGO
- **Type status:** Other material. **Occurrence:** catalogNumber: 12051; recordNumber: s.n.; recordedBy: Ayéna; lifeStage: Adult; reproductiveCondition: Fertile; **Taxon:** nameAccordingToID: The Plant List; scientificName: Pityrogramma calomelanos (L.) Link var. calomelanos; kingdom: Plantae; phylum: Monilophyta; class: Polypodiidae; order: Polypodiales; family: Pteridaceae; genus: Pityrogramma; specificEpithet: calomelanos; infraspecificEpithet: calomelanos; taxonRank: variety; scientificNameAuthorship: (L.) Link.; **Location:** continent: Africa; country: Togo; stateProvince: Plateaux; decimalLatitude: 7.0166667; decimalLongitude: 0.65; geodeticDatum: WGS 1984; **Identification:** identifiedBy: J.-F. Brunel; **Record Level:** institutionID: Herbarium togoense; collectionID: TOGO
- **Type status:** Other material. **Occurrence:** catalogNumber: 12052; recordNumber: 112; recordedBy: K. Akpagana; lifeStage: Adult; reproductiveCondition: Fertile; **Taxon:** nameAccordingToID: The Plant List; scientificName: Pityrogramma calomelanos (L.) Link var. calomelanos; kingdom: Plantae; phylum: Monilophyta; class: Polypodiidae; order: Polypodiales; family: Pteridaceae; genus: Pityrogramma; specificEpithet: calomelanos; infraspecificEpithet: calomelanos; taxonRank: variety; scientificNameAuthorship: (L.) Link.; **Location:** continent: Africa; country: Togo; stateProvince: Plateaux; decimalLatitude: 6.5833333; decimalLongitude: 0.75; geodeticDatum: WGS 1984; **Identification:** identifiedBy: K. Akpagana; dateIdentified: 1983; **Event:** eventDate: 1983; **Record Level:** institutionID: Herbarium togoense; collectionID: TOGO
- **Type status:** Other material. **Occurrence:** catalogNumber: 12053; recordNumber: 9817; recordedBy: J.-F. Brunel; lifeStage: Adult; reproductiveCondition: Fertile; **Taxon:** nameAccordingToID: The Plant List; scientificName: Pityrogramma calomelanos (L.) Link var. calomelanos; kingdom: Plantae; phylum: Monilophyta; class: Polypodiidae; order: Polypodiales; family: Pteridaceae; genus: Pityrogramma; specificEpithet: calomelanos; infraspecificEpithet: calomelanos; taxonRank: variety; scientificNameAuthorship: (L.) Link.; **Location:** continent: Africa; country: Togo; stateProvince: Maritime; decimalLatitude: 7.4666667; decimalLongitude: 0.9; geodeticDatum: WGS 1984; **Identification:** identifiedBy: J.-F. Brunel; dateIdentified: /1/1987; **Event:** eventDate: /1/1987; **Record Level:** institutionID: Herbarium togoense; collectionID: TOGO
- **Type status:** Other material. **Occurrence:** catalogNumber: 12054; recordNumber: 24; recordedBy: Djinadja; lifeStage: Adult; reproductiveCondition: Fertile; **Taxon:** nameAccordingToID: The Plant List; scientificName: Pityrogramma calomelanos (L.) Link var. calomelanos; kingdom: Plantae; phylum: Monilophyta; class: Polypodiidae; order: Polypodiales; family: Pteridaceae; genus: Pityrogramma; specificEpithet: calomelanos; infraspecificEpithet: calomelanos; taxonRank: variety; scientificNameAuthorship: (L.) Link.; **Location:** continent: Africa; country: Togo; stateProvince: Plateaux; decimalLatitude: 7; decimalLongitude: 0.75; geodeticDatum: WGS 1984; **Identification:** identifiedBy: Djinadja; dateIdentified: 23/3/1984; **Event:** eventDate: 23/3/1984; **Record Level:** institutionID: Herbarium togoense; collectionID: TOGO
- **Type status:** Other material. **Occurrence:** catalogNumber: 12055; recordNumber: 1236; recordedBy: J.-F. Brunel; lifeStage: Adult; reproductiveCondition: Fertile; **Taxon:** nameAccordingToID: The Plant List; scientificName: Pityrogramma calomelanos (L.) Link var. calomelanos; kingdom: Plantae; phylum: Monilophyta; class: Polypodiidae; order: Polypodiales; family: Pteridaceae; genus: Pityrogramma; specificEpithet: calomelanos; infraspecificEpithet: calomelanos; taxonRank: variety; scientificNameAuthorship: (L.) Link.; **Location:** continent: Africa; country: Togo; stateProvince: Plateaux; decimalLatitude: 7; decimalLongitude: 0.75; geodeticDatum: WGS 1984; **Identification:** identifiedBy: J.-F. Brunel; dateIdentified: /12/1974; **Event:** eventDate: /12/1974; **Record Level:** institutionID: Herbarium togoense; collectionID: TOGO
- **Type status:** Other material. **Occurrence:** catalogNumber: 12056; recordNumber: 162bis; recordedBy: K. Akpagana; lifeStage: Adult; reproductiveCondition: Fertile; **Taxon:** nameAccordingToID: The Plant List; scientificName: Pityrogramma calomelanos (L.) Link var. calomelanos; kingdom: Plantae; phylum: Monilophyta; class: Polypodiidae; order: Polypodiales; family: Pteridaceae; genus: Pityrogramma; specificEpithet: calomelanos; infraspecificEpithet: calomelanos; taxonRank: variety; scientificNameAuthorship: (L.) Link.; **Location:** continent: Africa; country: Togo; stateProvince: Centrale; decimalLatitude: 8.05; decimalLongitude: 0.7833333; geodeticDatum: WGS 1984; **Identification:** identifiedBy: K. Akpagana; dateIdentified: /6/1984; **Event:** eventDate: /6/1984; **Record Level:** institutionID: Herbarium togoense; collectionID: TOGO
- **Type status:** Other material. **Occurrence:** catalogNumber: 30033; recordNumber: 18; recordedBy: ABOTSI, SODJINOU & MINGOU; **Taxon:** nameAccordingToID: The Plant List; scientificName: Pityrogramma calomelanos (L.) Link var. calomelanos; kingdom: Plantae; phylum: Monilophyta; class: Polypodiidae; order: Polypodiales; family: Pteridaceae; genus: Pityrogramma; specificEpithet: calomelanos; infraspecificEpithet: calomelanos; taxonRank: variety; scientificNameAuthorship: (L.) Link.; **Location:** continent: Africa; country: Togo; stateProvince: Plateaux; decimalLatitude: 7.51402986; decimalLongitude: 0.59411437; geodeticDatum: WGS 1984; **Identification:** identifiedBy: K. E. Abotsi; dateIdentified: /05/2013; **Event:** eventDate: 04-03-13; **Record Level:** institutionID: Herbarium togoense; collectionID: TOGO
- **Type status:** Other material. **Occurrence:** catalogNumber: 30036; recordNumber: 49; recordedBy: ABOTSI, SODJINOU & MINGOU; **Taxon:** nameAccordingToID: The Plant List; scientificName: Pityrogramma calomelanos (L.) Link var. calomelanos; kingdom: Plantae; phylum: Monilophyta; class: Polypodiidae; order: Polypodiales; family: Pteridaceae; genus: Pityrogramma; specificEpithet: calomelanos; infraspecificEpithet: calomelanos; taxonRank: variety; scientificNameAuthorship: (L.) Link.; **Location:** continent: Africa; country: Togo; stateProvince: Plateaux; decimalLatitude: 8.17442335; decimalLongitude: 0.65783707; geodeticDatum: WGS 1984; **Identification:** identifiedBy: K. E. Abotsi; dateIdentified: /05/2013; **Event:** eventDate: 05-08-13; **Record Level:** institutionID: Herbarium togoense; collectionID: TOGO
- **Type status:** Other material. **Occurrence:** catalogNumber: 30045; recordNumber: 90; recordedBy: ABOTSI, SODJINOU & MINGOU; **Taxon:** nameAccordingToID: The Plant List; scientificName: Pityrogramma calomelanos (L.) Link var. calomelanos; kingdom: Plantae; phylum: Monilophyta; class: Polypodiidae; order: Polypodiales; family: Pteridaceae; genus: Pityrogramma; specificEpithet: calomelanos; infraspecificEpithet: calomelanos; taxonRank: variety; scientificNameAuthorship: (L.) Link.; **Location:** continent: Africa; country: Togo; stateProvince: Plateaux; decimalLatitude: 7.51381757; decimalLongitude: 0.61482743; geodeticDatum: WGS 1984; **Identification:** identifiedBy: K. E. Abotsi; dateIdentified: /05/2013; **Event:** eventDate: 04-04-13; **Record Level:** institutionID: Herbarium togoense; collectionID: TOGO
- **Type status:** Other material. **Occurrence:** catalogNumber: 30048; recordNumber: 93; recordedBy: ABOTSI, SODJINOU & MINGOU; **Taxon:** nameAccordingToID: The Plant List; scientificName: Pityrogramma calomelanos (L.) Link var. calomelanos; kingdom: Plantae; phylum: Monilophyta; class: Polypodiidae; order: Polypodiales; family: Pteridaceae; genus: Pityrogramma; specificEpithet: calomelanos; infraspecificEpithet: calomelanos; taxonRank: variety; scientificNameAuthorship: (L.) Link.; **Location:** continent: Africa; country: Togo; stateProvince: Plateaux; decimalLatitude: 7.51527922; decimalLongitude: 0.61602396; geodeticDatum: WGS 1984; **Identification:** identifiedBy: K. E. Abotsi; dateIdentified: /05/2013; **Event:** eventDate: 04-04-13; **Record Level:** institutionID: Herbarium togoense; collectionID: TOGO
- **Type status:** Other material. **Occurrence:** catalogNumber: 30054; recordNumber: 115; recordedBy: ABOTSI, SODJINOU & MINGOU; **Taxon:** nameAccordingToID: The Plant List; scientificName: Pityrogramma calomelanos (L.) Link var. calomelanos; kingdom: Plantae; phylum: Monilophyta; class: Polypodiidae; order: Polypodiales; family: Pteridaceae; genus: Pityrogramma; specificEpithet: calomelanos; infraspecificEpithet: calomelanos; taxonRank: variety; scientificNameAuthorship: (L.) Link.; **Location:** continent: Africa; country: Togo; stateProvince: Plateaux; decimalLatitude: 7.58213103; decimalLongitude: 0.62037669; geodeticDatum: WGS 1984; **Identification:** identifiedBy: K. E. Abotsi; dateIdentified: /05/2013; **Event:** eventDate: 04-05-13; **Record Level:** institutionID: Herbarium togoense; collectionID: TOGO
- **Type status:** Other material. **Occurrence:** catalogNumber: 30063; recordNumber: 159; recordedBy: ABOTSI, SODJINOU & MINGOU; **Taxon:** nameAccordingToID: The Plant List; scientificName: Pityrogramma calomelanos (L.) Link var. calomelanos; kingdom: Plantae; phylum: Monilophyta; class: Polypodiidae; order: Polypodiales; family: Pteridaceae; genus: Pityrogramma; specificEpithet: calomelanos; infraspecificEpithet: calomelanos; taxonRank: variety; scientificNameAuthorship: (L.) Link.; **Location:** continent: Africa; country: Togo; stateProvince: Plateaux; decimalLatitude: 8.19611892; decimalLongitude: 0.61687076; geodeticDatum: WGS 1984; **Identification:** identifiedBy: K. E. Abotsi; dateIdentified: /05/2013; **Event:** eventDate: 05-09-13; **Record Level:** institutionID: Herbarium togoense; collectionID: TOGO
- **Type status:** Other material. **Occurrence:** catalogNumber: 30077; recordNumber: 246; recordedBy: ABOTSI, SODJINOU & MINGOU; **Taxon:** nameAccordingToID: The Plant List; scientificName: Pityrogramma calomelanos (L.) Link var. calomelanos; kingdom: Plantae; phylum: Monilophyta; class: Polypodiidae; order: Polypodiales; family: Pteridaceae; genus: Pityrogramma; specificEpithet: calomelanos; infraspecificEpithet: calomelanos; taxonRank: variety; scientificNameAuthorship: (L.) Link.; **Location:** continent: Africa; country: Togo; stateProvince: Plateaux; decimalLatitude: 7.12286141; decimalLongitude: 0.65377744; geodeticDatum: WGS 1984; **Identification:** identifiedBy: K. E. Abotsi; dateIdentified: /05/2013; **Event:** eventDate: 04-09-13; **Record Level:** institutionID: Herbarium togoense; collectionID: TOGO
- **Type status:** Other material. **Occurrence:** catalogNumber: 30084; recordNumber: 281; recordedBy: ABOTSI, SODJINOU & MINGOU; **Taxon:** nameAccordingToID: The Plant List; scientificName: Pityrogramma calomelanos (L.) Link var. calomelanos; kingdom: Plantae; phylum: Monilophyta; class: Polypodiidae; order: Polypodiales; family: Pteridaceae; genus: Pityrogramma; specificEpithet: calomelanos; infraspecificEpithet: calomelanos; taxonRank: variety; scientificNameAuthorship: (L.) Link.; **Location:** continent: Africa; country: Togo; stateProvince: Plateaux; decimalLatitude: 7.1053695; decimalLongitude: 0.6073491; geodeticDatum: WGS 1984; **Identification:** identifiedBy: K. E. Abotsi; dateIdentified: /05/2013; **Event:** eventDate: 04-11-13; **Record Level:** institutionID: Herbarium togoense; collectionID: TOGO
- **Type status:** Other material. **Occurrence:** catalogNumber: 30087; recordNumber: 285; recordedBy: ABOTSI, SODJINOU & MINGOU; **Taxon:** nameAccordingToID: The Plant List; scientificName: Pityrogramma calomelanos (L.) Link var. calomelanos; kingdom: Plantae; phylum: Monilophyta; class: Polypodiidae; order: Polypodiales; family: Pteridaceae; genus: Pityrogramma; specificEpithet: calomelanos; infraspecificEpithet: calomelanos; taxonRank: variety; scientificNameAuthorship: (L.) Link.; **Location:** continent: Africa; country: Togo; stateProvince: Plateaux; decimalLatitude: 6.95506273; decimalLongitude: 0.58409449; geodeticDatum: WGS 1984; **Identification:** identifiedBy: K. E. Abotsi; dateIdentified: /05/2013; **Event:** eventDate: 04-15-13; **Record Level:** institutionID: Herbarium togoense; collectionID: TOGO
- **Type status:** Other material. **Occurrence:** catalogNumber: 30089; recordNumber: 290; recordedBy: ABOTSI, SODJINOU & MINGOU; **Taxon:** nameAccordingToID: The Plant List; scientificName: Pityrogramma calomelanos (L.) Link var. calomelanos; kingdom: Plantae; phylum: Monilophyta; class: Polypodiidae; order: Polypodiales; family: Pteridaceae; genus: Pityrogramma; specificEpithet: calomelanos; infraspecificEpithet: calomelanos; taxonRank: variety; scientificNameAuthorship: (L.) Link.; **Location:** continent: Africa; country: Togo; stateProvince: Plateaux; decimalLatitude: 6.95458625; decimalLongitude: 0.58282158; geodeticDatum: WGS 1984; **Identification:** identifiedBy: K. E. Abotsi; dateIdentified: /05/2013; **Event:** eventDate: 04-15-13; **Record Level:** institutionID: Herbarium togoense; collectionID: TOGO
- **Type status:** Other material. **Occurrence:** catalogNumber: 30100; recordNumber: 312; recordedBy: ABOTSI, SODJINOU & MINGOU; **Taxon:** nameAccordingToID: The Plant List; scientificName: Pityrogramma calomelanos (L.) Link var. calomelanos; kingdom: Plantae; phylum: Monilophyta; class: Polypodiidae; order: Polypodiales; family: Pteridaceae; genus: Pityrogramma; specificEpithet: calomelanos; infraspecificEpithet: calomelanos; taxonRank: variety; scientificNameAuthorship: (L.) Link.; **Location:** continent: Africa; country: Togo; stateProvince: Plateaux; decimalLatitude: 6.85892144; decimalLongitude: 0.75087921; geodeticDatum: WGS 1984; **Identification:** identifiedBy: K. E. Abotsi; dateIdentified: /05/2013; **Event:** eventDate: 04-16-13; **Record Level:** institutionID: Herbarium togoense; collectionID: TOGO
- **Type status:** Other material. **Occurrence:** catalogNumber: 30101; recordNumber: 317; recordedBy: ABOTSI, SODJINOU & MINGOU; **Taxon:** nameAccordingToID: The Plant List; scientificName: Pityrogramma calomelanos (L.) Link var. calomelanos; kingdom: Plantae; phylum: Monilophyta; class: Polypodiidae; order: Polypodiales; family: Pteridaceae; genus: Pityrogramma; specificEpithet: calomelanos; infraspecificEpithet: calomelanos; taxonRank: variety; scientificNameAuthorship: (L.) Link.; **Location:** continent: Africa; country: Togo; stateProvince: Plateaux; decimalLatitude: 6.86940731; decimalLongitude: 0.74903916; geodeticDatum: WGS 1984; **Identification:** identifiedBy: K. E. Abotsi; dateIdentified: /05/2013; **Event:** eventDate: 04-16-13; **Record Level:** institutionID: Herbarium togoense; collectionID: TOGO

##### Ecological interactions

###### Native status

Native

##### Distribution

Togo (Ecological Zones 2 and 4), Bahamas, Mexico, Belize, Guatemala, Honduras, El Salvador, Nicaragua, Costa Rica, Panama, Colombia, Venezuela, Ecuador, Peru, Bolivia, Brazil, Paraguay, Uruguay, St. Martin, St. Barthelemy, Antigua, Saba, St. Eustatius, St. Kitts, Montserrat, Guadeloupe, Marie Galante, Dominica, Martinique, St. Lucia, St. Vincent, Grenada, Barbados, Guyana, Surinam, French Guiana, Puerto Rico, Cuba, Jamaica, Hispaniola, Vieques Isl., Virgin Isl., Curacao, Isla del Coco, Australia (I), Taiwan (I), Ryukyu Isl. (I), China (I), Mozambique (I), Gabon (I), Uganda (I), Tanzania (I), Bioko Isl. (I), Sao Tomé and Principe (I), Senegal (I), Guinea (I), Sierra Leone (I), Liberia (I), Ivory Coast (I), Ghana (I), Nigeria (I), Cameroon (I), Gabon (I), Congo (I), Angola (I), D.R.Congo, Zambia (I), Madagascar (I), Mauritius (I), La Réunion (I), Comores (I), Seychelles (I), Burkina Faso (I), Marquesas Isl. (I), Society Isl. (I), Southern Marianas (I), Fiji (I), Palau Isl. (I), Micronesia (I), Hawaii (I), Malaysia (I), Borneo (I), Thailand (I), India (I), Sri Lanka (I), Andaman Isl. (I), Nicobar Isl. (I), Cambodia (I), Vietnam (I), Laos (I), Philippines (I), Sulawesi (I), Moluccas (I), USA (I)

##### Notes

Pityrogramma calomelanos var. calomelanos has a short rhizome, of about 8 mm diameter, wearing linear scales of more than 4 mm long, entire, brown clear. The fronds are arranged in tufts, erect to arching (Fig. 13a). The leaf blade has a herbaceous to slightly coriaceous texture. The petiole is 15 to 20 cm (sometimes more than 33 cm) long. It is black, shiny at ripe, channeled, covered with a few scales at the base. The leaf blade is oblong-lanceolate, 25-37 cm long and 10-14 cm wide, bipinnate to 3-pinnatifid. It consists of about 15 pairs of alternate lateral pinnae, making a 45° angle with the rachis, stalked. The lower pinnae are slightly reduced, middle ones reaching 10 to 12 cm long and 2.2 cm wide, with long tapered apex, pinnate at base, profoundly pinnatipartite up to the rachis. The pinnae are sessile, subopposite, about 0.5 cm long, lanceolate, upper base auriculate, entire, or lobed, acute at the apex. They wear a powdery white coating at the lower surface of the lamina (Fig. 13b). *Pityrogramma calomelanos* usually grows on rocks and roadsides, both in forests and more open environments.

#### Pteris atrovirens

Willd.

Pteris spinulifera Schum.

##### Materials

- **Type status:** Other material. **Occurrence:** catalogNumber: 12057; recordNumber: 8177; recordedBy: J.-F. Brunel; lifeStage: Adult; reproductiveCondition: Fertile; **Taxon:** nameAccordingToID: The Plant List; scientificName: *Pteris atrovirens* Willd.; kingdom: Plantae; phylum: Monilophyta; class: Polypodiidae; order: Polypodiales; family: Pteridaceae; genus: Pteris; specificEpithet: atrovirens; taxonRank: species; scientificNameAuthorship: Willd.; **Location:** continent: Africa; country: Togo; stateProvince: Plateaux; decimalLatitude: 7.5833333; decimalLongitude: 0.6; geodeticDatum: WGS 1984; **Identification:** identifiedBy: J.-F. Brunel; dateIdentified: /1/1984; **Event:** eventDate: /1/1984; **Record Level:** institutionID: Herbarium togoense; collectionID: TOGO
- **Type status:** Other material. **Occurrence:** catalogNumber: 12059; recordNumber: 7742; recordedBy: J.-F. Brunel; lifeStage: Adult; reproductiveCondition: Fertile; **Taxon:** nameAccordingToID: The Plant List; scientificName: *Pteris atrovirens* Willd.; kingdom: Plantae; phylum: Monilophyta; class: Polypodiidae; order: Polypodiales; family: Pteridaceae; genus: Pteris; specificEpithet: atrovirens; taxonRank: species; scientificNameAuthorship: Willd.; **Location:** continent: Africa; country: Togo; stateProvince: Centrale; decimalLatitude: 8.05; decimalLongitude: 0.7833333; geodeticDatum: WGS 1984; **Identification:** identifiedBy: J.-F. Brunel; dateIdentified: /6/1982; **Event:** eventDate: /6/1982; **Record Level:** institutionID: Herbarium togoense; collectionID: TOGO
- **Type status:** Other material. **Occurrence:** catalogNumber: 12062; recordNumber: 1142; recordedBy: K. Akpagana; lifeStage: Adult; reproductiveCondition: Fertile; **Taxon:** nameAccordingToID: The Plant List; scientificName: *Pteris atrovirens* Willd.; kingdom: Plantae; phylum: Monilophyta; class: Polypodiidae; order: Polypodiales; family: Pteridaceae; genus: Pteris; specificEpithet: atrovirens; taxonRank: species; scientificNameAuthorship: Willd.; **Location:** continent: Africa; country: Togo; stateProvince: Plateaux; decimalLatitude: 6.6666667; decimalLongitude: 1.1333333; geodeticDatum: WGS 1984; **Identification:** identifiedBy: K. Akpagana; dateIdentified: /11/1986; **Event:** eventDate: /11/1986; **Record Level:** institutionID: Herbarium togoense; collectionID: TOGO
- **Type status:** Other material. **Occurrence:** catalogNumber: 12065; recordNumber: 2169; recordedBy: J.-F. Brunel; lifeStage: Adult; reproductiveCondition: Fertile; **Taxon:** nameAccordingToID: The Plant List; scientificName: *Pteris atrovirens* Willd.; kingdom: Plantae; phylum: Monilophyta; class: Polypodiidae; order: Polypodiales; family: Pteridaceae; genus: Pteris; specificEpithet: atrovirens; taxonRank: species; scientificNameAuthorship: Willd.; **Location:** continent: Africa; country: Togo; stateProvince: Plateaux; decimalLatitude: 7; decimalLongitude: 0.75; geodeticDatum: WGS 1984; **Identification:** identifiedBy: J.-F. Brunel; dateIdentified: /5/1981; **Event:** eventDate: /5/1981; **Record Level:** institutionID: Herbarium togoense; collectionID: TOGO
- **Type status:** Other material. **Occurrence:** catalogNumber: 12068; recordNumber: 24; recordedBy: K. Akpagana; lifeStage: Adult; reproductiveCondition: Fertile; **Taxon:** nameAccordingToID: The Plant List; scientificName: *Pteris atrovirens* Willd.; kingdom: Plantae; phylum: Monilophyta; class: Polypodiidae; order: Polypodiales; family: Pteridaceae; genus: Pteris; specificEpithet: atrovirens; taxonRank: species; scientificNameAuthorship: Willd.; **Location:** continent: Africa; country: Togo; stateProvince: Centrale; decimalLatitude: 8.05; decimalLongitude: 0.7833333; geodeticDatum: WGS 1984; **Identification:** identifiedBy: K. Akpagana; dateIdentified: /6/1982; **Event:** eventDate: /6/1982; **Record Level:** institutionID: Herbarium togoense; collectionID: TOGO
- **Type status:** Other material. **Occurrence:** catalogNumber: 30035; recordNumber: 37; recordedBy: ABOTSI, SODJINOU & MINGOU; **Taxon:** nameAccordingToID: The Plant List; scientificName: *Pteris atrovirens* Willd.; kingdom: Plantae; phylum: Monilophyta; class: Polypodiidae; order: Polypodiales; family: Pteridaceae; genus: Pteris; specificEpithet: atrovirens; taxonRank: species; scientificNameAuthorship: Willd.; **Location:** continent: Africa; country: Togo; stateProvince: Plateaux; decimalLatitude: 7.51687216; decimalLongitude: 0.59161727; geodeticDatum: WGS 1984; **Identification:** identifiedBy: K. E. Abotsi; dateIdentified: /05/2013; **Event:** eventDate: 04-03-13; **Record Level:** institutionID: Herbarium togoense; collectionID: TOGO
- **Type status:** Other material. **Occurrence:** catalogNumber: 30038; recordNumber: 54; recordedBy: ABOTSI, SODJINOU & MINGOU; **Taxon:** nameAccordingToID: The Plant List; scientificName: *Pteris atrovirens* Willd.; kingdom: Plantae; phylum: Monilophyta; class: Polypodiidae; order: Polypodiales; family: Pteridaceae; genus: Pteris; specificEpithet: atrovirens; taxonRank: species; scientificNameAuthorship: Willd.; **Location:** continent: Africa; country: Togo; stateProvince: Plateaux; decimalLatitude: 8.17450538; decimalLongitude: 0.65795451; geodeticDatum: WGS 1984; **Identification:** identifiedBy: K. E. Abotsi; dateIdentified: /05/2013; **Event:** eventDate: 05-08-13; **Record Level:** institutionID: Herbarium togoense; collectionID: TOGO
- **Type status:** Other material. **Occurrence:** catalogNumber: 30039; recordNumber: 55; recordedBy: ABOTSI, SODJINOU & MINGOU; **Taxon:** nameAccordingToID: The Plant List; scientificName: *Pteris atrovirens* Willd.; kingdom: Plantae; phylum: Monilophyta; class: Polypodiidae; order: Polypodiales; family: Pteridaceae; genus: Pteris; specificEpithet: atrovirens; taxonRank: species; scientificNameAuthorship: Willd.; **Location:** continent: Africa; country: Togo; stateProvince: Plateaux; decimalLatitude: 8.17450538; decimalLongitude: 0.65795451; geodeticDatum: WGS 1984; **Identification:** identifiedBy: K. E. Abotsi; dateIdentified: /05/2013; **Event:** eventDate: 05-08-13; **Record Level:** institutionID: Herbarium togoense; collectionID: TOGO
- **Type status:** Other material. **Occurrence:** catalogNumber: 30040; recordNumber: 56; recordedBy: ABOTSI, SODJINOU & MINGOU; **Taxon:** nameAccordingToID: The Plant List; scientificName: *Pteris atrovirens* Willd.; kingdom: Plantae; phylum: Monilophyta; class: Polypodiidae; order: Polypodiales; family: Pteridaceae; genus: Pteris; specificEpithet: atrovirens; taxonRank: species; scientificNameAuthorship: Willd.; **Location:** continent: Africa; country: Togo; stateProvince: Plateaux; decimalLatitude: 8.17450538; decimalLongitude: 0.65795451; geodeticDatum: WGS 1984; **Identification:** identifiedBy: K. E. Abotsi; dateIdentified: /05/2013; **Event:** eventDate: 05-08-13; **Record Level:** institutionID: Herbarium togoense; collectionID: TOGO
- **Type status:** Other material. **Occurrence:** catalogNumber: 30042; recordNumber: 75; recordedBy: ABOTSI, SODJINOU & MINGOU; **Taxon:** nameAccordingToID: The Plant List; scientificName: *Pteris atrovirens* Willd.; kingdom: Plantae; phylum: Monilophyta; class: Polypodiidae; order: Polypodiales; family: Pteridaceae; genus: Pteris; specificEpithet: atrovirens; taxonRank: species; scientificNameAuthorship: Willd.; **Location:** continent: Africa; country: Togo; stateProvince: Plateaux; decimalLatitude: 7.51358779; decimalLongitude: 0.61412229; geodeticDatum: WGS 1984; **Identification:** identifiedBy: K. E. Abotsi; dateIdentified: /05/2013; **Event:** eventDate: 04-04-13; **Record Level:** institutionID: Herbarium togoense; collectionID: TOGO
- **Type status:** Other material. **Occurrence:** catalogNumber: 30043; recordNumber: 76; recordedBy: ABOTSI, SODJINOU & MINGOU; **Taxon:** nameAccordingToID: The Plant List; scientificName: *Pteris atrovirens* Willd.; kingdom: Plantae; phylum: Monilophyta; class: Polypodiidae; order: Polypodiales; family: Pteridaceae; genus: Pteris; specificEpithet: atrovirens; taxonRank: species; scientificNameAuthorship: Willd.; **Location:** continent: Africa; country: Togo; stateProvince: Plateaux; decimalLatitude: 7.51358779; decimalLongitude: 0.61412229; geodeticDatum: WGS 1984; **Identification:** identifiedBy: K. E. Abotsi; dateIdentified: /05/2013; **Event:** eventDate: 04-04-13; **Record Level:** institutionID: Herbarium togoense; collectionID: TOGO
- **Type status:** Other material. **Occurrence:** catalogNumber: 30060; recordNumber: 153; recordedBy: ABOTSI, SODJINOU & MINGOU; **Taxon:** nameAccordingToID: The Plant List; scientificName: *Pteris atrovirens* Willd.; kingdom: Plantae; phylum: Monilophyta; class: Polypodiidae; order: Polypodiales; family: Pteridaceae; genus: Pteris; specificEpithet: atrovirens; taxonRank: species; scientificNameAuthorship: Willd.; **Location:** continent: Africa; country: Togo; stateProvince: Plateaux; decimalLatitude: 8.1959055; decimalLongitude: 0.61440464; geodeticDatum: WGS 1984; **Identification:** identifiedBy: K. E. Abotsi; dateIdentified: /05/2013; **Event:** eventDate: 05-09-13; **Record Level:** institutionID: Herbarium togoense; collectionID: TOGO

##### Ecological interactions

###### Native status

Native

##### Distribution

Togo (Ecological Zone 4), Gabon, Comores, Uganda, Kenya, Tanzania, Pemba Isl., Principe Isl., Sao Tomé, Bioko Isl., Guinea, Sierra Leone, Liberia, Ivory Coast, Ghana, Benin, Nigeria, Cameroon, Central African Republic, Equatorial Guinea, Congo, D.R.Congo, Angola, Burundi, Sudan, Seychelles

##### Notes

The rhizome of *Pteris atrovirens* is short and covered by dark lanceolate scales of about 4 mm long, with pale margins. The fronds are organized in tufts, from 0.25 to 1 m long. The petiole is 20 to 50 cm long, straw-colored, reddish or purple at the base. The leaf blade is elliptic-lanceolate, 20-50 cm long and 15-60 cm wide, bipinnatifid, composed of 5 to 7 pairs of lateral pinnae. Upper pinnae are opposed. The middle ones are alternate, short-stalked or subsessile, cuneate at the base, divided into about 15 rounded segments, oblong, toothed at the top, separated by narrow sinus (Fig. 14a). The pinnae apices are entire and shortly tapered on 1.5 to 2 cm. The lower pinnae are stalked, with pinnatifid auricule. The terminal pinnae are shortly decurrent. The rachis is smooth, purplish-brown. There are some soft protuberances at the upper base of pinnae (Fig. 14b). The ribs form a costal lowered areola and 1-2 series of areolas between the costa and the margin. The sori are linear. They are almost all around the lobes, up to the apex serrations. The plant prefers the banks of rivers and moist soils of forest galleries.

#### Pteris burtonii

Bak.

Pteris johnstonii Bak, *P. aethiopica* Christ, P. atrovirens var. cervonii Bonap, P. burtonii var. aethiopica (Christ) Tardieu.

##### Materials

- **Type status:** Other material. **Occurrence:** catalogNumber: 12069; recordNumber: 1371bis; recordedBy: K. Akpagana; lifeStage: Adult; reproductiveCondition: Fertile; **Taxon:** nameAccordingToID: The Plant List; scientificName: *Pteris burtonii* Baker; kingdom: Plantae; phylum: Monilophyta; class: Polypodiidae; order: Polypodiales; family: Pteridaceae; genus: Pteris; specificEpithet: burtoni; taxonRank: species; scientificNameAuthorship: Baker.; **Location:** continent: Africa; country: Togo; stateProvince: Plateaux; decimalLatitude: 7; decimalLongitude: 0.75; geodeticDatum: WGS 1984; **Identification:** identifiedBy: K. Akpagana; dateIdentified: /2/1987; **Event:** eventDate: /2/1987; **Record Level:** institutionID: Herbarium togoense; collectionID: TOGO
- **Type status:** Other material. **Occurrence:** catalogNumber: 12070; recordNumber: 2427; recordedBy: K. Akpagana; lifeStage: Adult; reproductiveCondition: Fertile; **Taxon:** nameAccordingToID: The Plant List; scientificName: *Pteris burtonii* Baker; kingdom: Plantae; phylum: Monilophyta; class: Polypodiidae; order: Polypodiales; family: Pteridaceae; genus: Pteris; specificEpithet: burtoni; taxonRank: species; scientificNameAuthorship: Baker.; **Location:** continent: Africa; country: Togo; stateProvince: Centrale; decimalLatitude: 9.25; decimalLongitude: 1.2; geodeticDatum: WGS 1984; **Identification:** identifiedBy: K. Akpagana; dateIdentified: 1993; **Event:** eventDate: 1993; **Record Level:** institutionID: Herbarium togoense; collectionID: TOGO
- **Type status:** Other material. **Occurrence:** catalogNumber: 12072; recordNumber: 5591; recordedBy: J.-F. Brunel; lifeStage: Adult; reproductiveCondition: Fertile; **Taxon:** nameAccordingToID: The Plant List; scientificName: *Pteris burtonii* Baker; kingdom: Plantae; phylum: Monilophyta; class: Polypodiidae; order: Polypodiales; family: Pteridaceae; genus: Pteris; specificEpithet: burtoni; taxonRank: species; scientificNameAuthorship: Baker.; **Location:** continent: Africa; country: Togo; stateProvince: Plateaux; decimalLatitude: 7; decimalLongitude: 0.75; geodeticDatum: WGS 1984; **Identification:** identifiedBy: J.-F. Brunel; **Record Level:** institutionID: Herbarium togoense; collectionID: TOGO
- **Type status:** Other material. **Occurrence:** catalogNumber: 30031; recordNumber: 16; recordedBy: ABOTSI, SODJINOU & MINGOU; **Taxon:** nameAccordingToID: The Plant List; scientificName: *Pteris burtonii* Baker; kingdom: Plantae; phylum: Monilophyta; class: Polypodiidae; order: Polypodiales; family: Pteridaceae; genus: Pteris; specificEpithet: burtonii; taxonRank: species; scientificNameAuthorship: Baker.; **Location:** continent: Africa; country: Togo; stateProvince: Plateaux; decimalLatitude: 7.51380764; decimalLongitude: 0.59479481; geodeticDatum: WGS 1984; **Identification:** identifiedBy: K. E. Abotsi; dateIdentified: /05/2013; **Event:** eventDate: 04-03-13; **Record Level:** institutionID: Herbarium togoense; collectionID: TOGO
- **Type status:** Other material. **Occurrence:** catalogNumber: 30032; recordNumber: 17; recordedBy: ABOTSI, SODJINOU & MINGOU; **Taxon:** nameAccordingToID: The Plant List; scientificName: *Pteris burtonii* Baker; kingdom: Plantae; phylum: Monilophyta; class: Polypodiidae; order: Polypodiales; family: Pteridaceae; genus: Pteris; specificEpithet: burtonii; taxonRank: species; scientificNameAuthorship: Baker.; **Location:** continent: Africa; country: Togo; stateProvince: Plateaux; decimalLatitude: 7.51380764; decimalLongitude: 0.59479481; geodeticDatum: WGS 1984; **Identification:** identifiedBy: K. E. Abotsi; dateIdentified: /05/2013; **Event:** eventDate: 04-03-13; **Record Level:** institutionID: Herbarium togoense; collectionID: TOGO
- **Type status:** Other material. **Occurrence:** catalogNumber: 30059; recordNumber: 152; recordedBy: ABOTSI, SODJINOU & MINGOU; **Taxon:** nameAccordingToID: The Plant List; scientificName: *Pteris burtonii* Baker; kingdom: Plantae; phylum: Monilophyta; class: Polypodiidae; order: Polypodiales; family: Pteridaceae; genus: Pteris; specificEpithet: burtonii; taxonRank: species; scientificNameAuthorship: Baker.; **Location:** continent: Africa; country: Togo; stateProvince: Plateaux; decimalLatitude: 8.1959055; decimalLongitude: 0.61440464; geodeticDatum: WGS 1984; **Identification:** identifiedBy: K. E. Abotsi; dateIdentified: /05/2013; **Event:** eventDate: 05-09-13; **Record Level:** institutionID: Herbarium togoense; collectionID: TOGO
- **Type status:** Other material. **Occurrence:** catalogNumber: 30069; recordNumber: 174; recordedBy: ABOTSI, SODJINOU & MINGOU; **Taxon:** nameAccordingToID: The Plant List; scientificName: *Pteris burtonii* Baker; kingdom: Plantae; phylum: Monilophyta; class: Polypodiidae; order: Polypodiales; family: Pteridaceae; genus: Pteris; specificEpithet: burtonii; taxonRank: species; scientificNameAuthorship: Baker.; **Location:** continent: Africa; country: Togo; stateProvince: Plateaux; decimalLatitude: 8.01520112; decimalLongitude: 0.63320513; geodeticDatum: WGS 1984; **Identification:** identifiedBy: K. E. Abotsi; dateIdentified: /05/2013; **Event:** eventDate: 05-11-13; **Record Level:** institutionID: Herbarium togoense; collectionID: TOGO
- **Type status:** Other material. **Occurrence:** catalogNumber: 30109; recordNumber: 353; recordedBy: ABOTSI, SODJINOU & MINGOU; **Taxon:** nameAccordingToID: The Plant List; scientificName: *Pteris burtonii* Baker; kingdom: Plantae; phylum: Monilophyta; class: Polypodiidae; order: Polypodiales; family: Pteridaceae; genus: Pteris; specificEpithet: burtonii; taxonRank: species; scientificNameAuthorship: Baker.; **Location:** continent: Africa; country: Togo; stateProvince: Plateaux; decimalLatitude: 6.94840325; decimalLongitude: 0.57909955; geodeticDatum: WGS 1984; **Identification:** identifiedBy: K. E. Abotsi; dateIdentified: /05/2013; **Event:** eventDate: 04-15-13; **Record Level:** institutionID: Herbarium togoense; collectionID: TOGO

##### Ecological interactions

###### Native status

Native

##### Distribution

Togo (Ecological Zones 2, 4 and 5), Guinea, Sierra Leone, Tanzania, Principe Isl., Bioko Isl., Ivory Coast, Liberia, Ghana, Nigeria, Cameroon, Central African Republic, Gabon, Congo, D.R.Congo, Angola, Burundi

##### Notes

The rhizome of *Pteris burtonii* is short, erect, covered with black scales, with pale margins, lanceolate to linear-lanceolate (3 to 4 mm long). The fronds are tufted, 0.3 to 1m long (Fig. 15a). The stem measures 10 to 38 cm long and is covered with scales at the base. The leaf blade is deltoid, lanceolate, 20-40 cm long and 15-40 cm wide, with a bud near the base of the terminal pinnae. Each frond has 1-5 pairs of simple pinnae, oblong-lanceolate, entire or slightly lobed, of 6 to 19 cm long and 1.4 to 4 cm wide. The two basal pinnae has a basiscopic spur. The pinnae are often pinnatifid, sometimes simple. Both types of fronds can coexist on the same plant. The terminal segment is deltoid-lanceolate, 3 to 6 (-9) cm long and about 1 cm wide, toothed at the apex. The ultimate pinnae segments are organized into 10-15 pairs, oblong. The basal basiscopic segment of lowest pinnae is longer. The stipe is slightly winged by decurrent leaf base. The ribs are anastomosed at the segments level. The sori are present on all the margin of the pinnae (simple as pinnatifid ones) without interruption in the sinus, but the apex of the ultimate segments is generally sterile and toothed, giving a U-shaped sori (Fig. 15b). *Pteris burtonii* usually grows on the banks of rivers, waterfalls, cliffs and wet roadsides in forest areas.

#### Pteris similis

Kuhn.

Pteris congoensis Christ., *P. spinulifera* sensu Tardieu.

##### Materials

- **Type status:** Other material. **Occurrence:** catalogNumber: 30065; recordNumber: 162; recordedBy: ABOTSI, SODJINOU & MINGOU; **Taxon:** nameAccordingToID: The Plant List; scientificName: *Pteris similis* Kuhn.; kingdom: Plantae; phylum: Monilophyta; class: Polypodiidae; order: Polypodiales; family: Pteridaceae; genus: Pteris; specificEpithet: similis; taxonRank: species; scientificNameAuthorship: Kuhn.; **Location:** continent: Africa; country: Togo; stateProvince: Plateaux; decimalLatitude: 8.19669463; decimalLongitude: 0.61947079; geodeticDatum: WGS 1984; **Identification:** identifiedBy: K. E. Abotsi; dateIdentified: /05/2013; **Event:** eventDate: 05-09-13; **Record Level:** institutionID: Herbarium togoense; collectionID: TOGO

##### Ecological interactions

###### Native status

Native

##### Distribution

Togo (Ecological Zone 4), Uganda, Tanzania, Principe Isl., Sao Tomé, Bioko Isl., Guinea, Mali, Sierra Leone, Liberia, Ivory Coast, Ghana, Benin, Nigeria, Cameroon, Central African Republic, Gabon, Congo, D.R.Congo, Angola, Sudan

##### Notes

*Pteris similis* is a terrestrial fern, with an erect rhizome (up to 15 cm), with fronds in clumps, coverd by black scales, linear or linear-lanceolate. The fronds are solitary or tufted, reaching 1.8 to 4 (-6) m tall, often rooting at the apex (Fig. 16a). The petiole is straw colored, purplish or brownish at the base. It measures 30 to 50 cm long. It is channeled at the top, naked and muriculate. The lamina is pinnate, lanceolate. It measures 0.5 to 3.4 (-5.5) m long, sometimes more than 60 cm wide. Pinnae (10-30 pairs), pinnatifid, are oblong-lanceolate and measure 8-35 cm over 3.2-12.5 cm. The ultimate segments (12-28 pairs) are oblong-lanceolate, 1-8.5 cm long and 3-10 mm large. The terminal segment is 1.5 to 4cm long. Some terminals segments are pinnate, making some pinnae partially bipinnatifid. The lower pinnae are auriculate (Fig. 16b). The rachis is stramineous, muriculate or spiny. The costae and the costulae bear large spines on the underside. There is no buds at the base of the terminal pinnae. The lamina texture is membranous. The ribs form a lowered areola along the costa of pinnae, and 1 or 2 series of areolas along the midrib of the lobes. The sori occupy 3/4 to 7/8 of the lobes, down to the base of sinuses (Fig. 16c, d). The apex is sterile and toothed. *Pteris similis* grows generally on the banks of rivers, in rainforest areas.

#### Pteris togoensis

Hieron.

Pteris kamerunensis Hieron., *P. quadriaurita* sensu Sim., *P. biaurita* sensu Sim.

##### Materials

- **Type status:** Other material. **Occurrence:** catalogNumber: 11779; recordNumber: 23; recordedBy: K. Akpagana; lifeStage: Adult; reproductiveCondition: Fertile; **Taxon:** nameAccordingToID: The Plant List; scientificName: *Pteris togoensis* Hieron.; kingdom: Plantae; phylum: Monilophyta; class: Polypodiidae; order: Polypodiales; family: Pteridaceae; genus: Pteris; specificEpithet: togoensis; taxonRank: species; scientificNameAuthorship: Hieron.; **Location:** continent: Africa; country: Togo; stateProvince: Centrale; decimalLatitude: 8.05; decimalLongitude: 0.7833333; geodeticDatum: WGS 1984; **Identification:** identifiedBy: K. Akpagana; dateIdentified: /6/1982; **Event:** eventDate: /6/1982; **Record Level:** institutionID: Herbarium togoense; collectionID: TOGO
- **Type status:** Other material. **Occurrence:** catalogNumber: 12079; recordNumber: 9421; recordedBy: J.-F. Brunel; lifeStage: Adult; reproductiveCondition: Fertile; **Taxon:** nameAccordingToID: The Plant List; scientificName: *Pteris togoensis* Hieron.; kingdom: Plantae; phylum: Monilophyta; class: Polypodiidae; order: Polypodiales; family: Pteridaceae; genus: Pteris; specificEpithet: togoensis; taxonRank: species; scientificNameAuthorship: Hieron.; **Location:** continent: Africa; country: Togo; stateProvince: Plateaux; decimalLatitude: 7.6666667; decimalLongitude: 0.8; geodeticDatum: WGS 1984; **Identification:** identifiedBy: J.-F. Brunel; dateIdentified: /2/1986; **Event:** eventDate: /2/1986; **Record Level:** institutionID: Herbarium togoense; collectionID: TOGO
- **Type status:** Other material. **Occurrence:** catalogNumber: 12081; recordNumber: 67; recordedBy: J.N. Terrible; lifeStage: Adult; reproductiveCondition: Fertile; **Taxon:** nameAccordingToID: The Plant List; scientificName: *Pteris togoensis* Hieron.; kingdom: Plantae; phylum: Monilophyta; class: Polypodiidae; order: Polypodiales; family: Pteridaceae; genus: Pteris; specificEpithet: togoensis; taxonRank: species; scientificNameAuthorship: Hieron.; **Location:** continent: Africa; country: Togo; stateProvince: Centrale; decimalLatitude: 8.7; decimalLongitude: 0.7666667; geodeticDatum: WGS 1984; **Identification:** identifiedBy: J.-F. Brunel; dateIdentified: 1973; **Event:** eventDate: 1973; **Record Level:** institutionID: Herbarium togoense; collectionID: TOGO
- **Type status:** Other material. **Occurrence:** catalogNumber: 12082; recordNumber: 67bis; recordedBy: J.N. Terrible; lifeStage: Adult; reproductiveCondition: Fertile; **Taxon:** nameAccordingToID: The Plant List; scientificName: *Pteris togoensis* Hieron.; kingdom: Plantae; phylum: Monilophyta; class: Polypodiidae; order: Polypodiales; family: Pteridaceae; genus: Pteris; specificEpithet: togoensis; taxonRank: species; scientificNameAuthorship: Hieron.; **Location:** continent: Africa; country: Togo; stateProvince: Centrale; decimalLatitude: 8.7; decimalLongitude: 0.7666667; geodeticDatum: WGS 1984; **Identification:** identifiedBy: C.A. Meyer; dateIdentified: 1980; **Event:** eventDate: 1973; **Record Level:** institutionID: Herbarium togoense; collectionID: TOGO
- **Type status:** Other material. **Occurrence:** catalogNumber: 12083; recordNumber: 9830; recordedBy: J.-F. Brunel; lifeStage: Adult; reproductiveCondition: Fertile; **Taxon:** nameAccordingToID: The Plant List; scientificName: *Pteris togoensis* Hieron.; kingdom: Plantae; phylum: Monilophyta; class: Polypodiidae; order: Polypodiales; family: Pteridaceae; genus: Pteris; specificEpithet: togoensis; taxonRank: species; scientificNameAuthorship: Hieron.; **Location:** continent: Africa; country: Togo; stateProvince: Maritime; decimalLatitude: 7.4666667; decimalLongitude: 0.9; geodeticDatum: WGS 1984; **Identification:** identifiedBy: J.-F. Brunel; dateIdentified: /1/1987; **Event:** eventDate: /1/1987; **Record Level:** institutionID: Herbarium togoense; collectionID: TOGO
- **Type status:** Other material. **Occurrence:** catalogNumber: 12086; recordNumber: 3747; recordedBy: J.-F. Brunel; lifeStage: Adult; reproductiveCondition: Fertile; **Taxon:** nameAccordingToID: The Plant List; scientificName: *Pteris togoensis* Hieron.; kingdom: Plantae; phylum: Monilophyta; class: Polypodiidae; order: Polypodiales; family: Pteridaceae; genus: Pteris; specificEpithet: togoensis; taxonRank: species; scientificNameAuthorship: Hieron.; **Location:** continent: Africa; country: Togo; stateProvince: Plateaux; decimalLatitude: 7; decimalLongitude: 0.75; geodeticDatum: WGS 1984; **Identification:** identifiedBy: C.A. Meyer; dateIdentified: 1980; **Event:** eventDate: 1975; **Record Level:** institutionID: Herbarium togoense; collectionID: TOGO
- **Type status:** Other material. **Occurrence:** catalogNumber: 12087; recordNumber: 8192; recordedBy: J.-F. Brunel; lifeStage: Adult; reproductiveCondition: Fertile; **Taxon:** nameAccordingToID: The Plant List; scientificName: *Pteris togoensis* Hieron.; kingdom: Plantae; phylum: Monilophyta; class: Polypodiidae; order: Polypodiales; family: Pteridaceae; genus: Pteris; specificEpithet: togoensis; taxonRank: species; scientificNameAuthorship: Hieron.; **Location:** continent: Africa; country: Togo; stateProvince: Plateaux; decimalLatitude: 6.6666667; decimalLongitude: 1.1333333; geodeticDatum: WGS 1984; **Identification:** identifiedBy: J.-F. Brunel; dateIdentified: /1/1984; **Event:** eventDate: /1/1984; **Record Level:** institutionID: Herbarium togoense; collectionID: TOGO
- **Type status:** Other material. **Occurrence:** catalogNumber: 12090; recordNumber: 60; recordedBy: K. Akpagana; lifeStage: Adult; reproductiveCondition: Fertile; **Taxon:** nameAccordingToID: The Plant List; scientificName: *Pteris togoensis* Hieron.; kingdom: Plantae; phylum: Monilophyta; class: Polypodiidae; order: Polypodiales; family: Pteridaceae; genus: Pteris; specificEpithet: togoensis; taxonRank: species; scientificNameAuthorship: Hieron.; **Location:** continent: Africa; country: Togo; stateProvince: Centrale; decimalLatitude: 8.1833333; decimalLongitude: 0.65; geodeticDatum: WGS 1984; **Identification:** identifiedBy: K. Akpagana; dateIdentified: /10/1982; **Event:** eventDate: /10/1982; **Record Level:** institutionID: Herbarium togoense; collectionID: TOGO
- **Type status:** Other material. **Occurrence:** catalogNumber: 30027; recordNumber: 8; recordedBy: ABOTSI, SODJINOU & MINGOU; **Taxon:** nameAccordingToID: The Plant List; scientificName: *Pteris togoensis* Hieron.; kingdom: Plantae; phylum: Monilophyta; class: Polypodiidae; order: Polypodiales; family: Pteridaceae; genus: Pteris; specificEpithet: togoensis; taxonRank: species; scientificNameAuthorship: Hieron.; **Location:** continent: Africa; country: Togo; stateProvince: Plateaux; decimalLatitude: 7.51362435; decimalLongitude: 0.5959822; geodeticDatum: WGS 1984; **Identification:** identifiedBy: K. E. Abotsi; dateIdentified: /05/2013; **Event:** eventDate: 04-03-13; **Record Level:** institutionID: Herbarium togoense; collectionID: TOGO
- **Type status:** Other material. **Occurrence:** catalogNumber: 30037; recordNumber: 53; recordedBy: ABOTSI, SODJINOU & MINGOU; **Taxon:** nameAccordingToID: The Plant List; scientificName: *Pteris togoensis* Hieron.; kingdom: Plantae; phylum: Monilophyta; class: Polypodiidae; order: Polypodiales; family: Pteridaceae; genus: Pteris; specificEpithet: togoensis; taxonRank: species; scientificNameAuthorship: Hieron.; **Location:** continent: Africa; country: Togo; stateProvince: Plateaux; decimalLatitude: 8.17442335; decimalLongitude: 0.65783707; geodeticDatum: WGS 1984; **Identification:** identifiedBy: K. E. Abotsi; dateIdentified: /05/2013; **Event:** eventDate: 05-08-13; **Record Level:** institutionID: Herbarium togoense; collectionID: TOGO
- **Type status:** Other material. **Occurrence:** catalogNumber: 30051; recordNumber: 108; recordedBy: ABOTSI, SODJINOU & MINGOU; **Taxon:** nameAccordingToID: The Plant List; scientificName: *Pteris togoensis* Hieron.; kingdom: Plantae; phylum: Monilophyta; class: Polypodiidae; order: Polypodiales; family: Pteridaceae; genus: Pteris; specificEpithet: togoensis; taxonRank: species; scientificNameAuthorship: Hieron.; **Location:** continent: Africa; country: Togo; stateProvince: Plateaux; decimalLatitude: 7.58277662; decimalLongitude: 0.61448552; geodeticDatum: WGS 1984; **Identification:** identifiedBy: K. E. Abotsi; dateIdentified: /05/2013; **Event:** eventDate: 04-05-13; **Record Level:** institutionID: Herbarium togoense; collectionID: TOGO
- **Type status:** Other material. **Occurrence:** catalogNumber: 30052; recordNumber: 109; recordedBy: ABOTSI, SODJINOU & MINGOU; **Taxon:** nameAccordingToID: The Plant List; scientificName: *Pteris togoensis* Hieron.; kingdom: Plantae; phylum: Monilophyta; class: Polypodiidae; order: Polypodiales; family: Pteridaceae; genus: Pteris; specificEpithet: togoensis; taxonRank: species; scientificNameAuthorship: Hieron.; **Location:** continent: Africa; country: Togo; stateProvince: Plateaux; decimalLatitude: 7.58306294; decimalLongitude: 0.61890419; geodeticDatum: WGS 1984; **Identification:** identifiedBy: K. E. Abotsi; dateIdentified: /05/2013; **Event:** eventDate: 04-05-13; **Record Level:** institutionID: Herbarium togoense; collectionID: TOGO
- **Type status:** Other material. **Occurrence:** catalogNumber: 30053; recordNumber: 113; recordedBy: ABOTSI, SODJINOU & MINGOU; **Taxon:** nameAccordingToID: The Plant List; scientificName: *Pteris togoensis* Hieron.; kingdom: Plantae; phylum: Monilophyta; class: Polypodiidae; order: Polypodiales; family: Pteridaceae; genus: Pteris; specificEpithet: togoensis; taxonRank: species; scientificNameAuthorship: Hieron.; **Location:** continent: Africa; country: Togo; stateProvince: Plateaux; decimalLatitude: 7.58247905; decimalLongitude: 0.61955957; geodeticDatum: WGS 1984; **Identification:** identifiedBy: K. E. Abotsi; dateIdentified: /05/2013; **Event:** eventDate: 04-05-13; **Record Level:** institutionID: Herbarium togoense; collectionID: TOGO
- **Type status:** Other material. **Occurrence:** catalogNumber: 30062; recordNumber: 156; recordedBy: ABOTSI, SODJINOU & MINGOU; **Taxon:** nameAccordingToID: The Plant List; scientificName: *Pteris togoensis* Hieron.; kingdom: Plantae; phylum: Monilophyta; class: Polypodiidae; order: Polypodiales; family: Pteridaceae; genus: Pteris; specificEpithet: togoensis; taxonRank: species; scientificNameAuthorship: Hieron.; **Location:** continent: Africa; country: Togo; stateProvince: Plateaux; decimalLatitude: 8.19615939; decimalLongitude: 0.61607224; geodeticDatum: WGS 1984; **Identification:** identifiedBy: K. E. Abotsi; dateIdentified: /05/2013; **Event:** eventDate: 05-09-13; **Record Level:** institutionID: Herbarium togoense; collectionID: TOGO
- **Type status:** Other material. **Occurrence:** catalogNumber: 30070; recordNumber: 175; recordedBy: ABOTSI, SODJINOU & MINGOU; **Taxon:** nameAccordingToID: The Plant List; scientificName: *Pteris togoensis* Hieron.; kingdom: Plantae; phylum: Monilophyta; class: Polypodiidae; order: Polypodiales; family: Pteridaceae; genus: Pteris; specificEpithet: togoensis; taxonRank: species; scientificNameAuthorship: Hieron.; **Location:** continent: Africa; country: Togo; stateProvince: Plateaux; decimalLatitude: 8.01520112; decimalLongitude: 0.63320513; geodeticDatum: WGS 1984; **Identification:** identifiedBy: K. E. Abotsi; dateIdentified: /05/2013; **Event:** eventDate: 05-11-13; **Record Level:** institutionID: Herbarium togoense; collectionID: TOGO
- **Type status:** Other material. **Occurrence:** catalogNumber: 30076; recordNumber: 232; recordedBy: ABOTSI, SODJINOU & MINGOU; **Taxon:** nameAccordingToID: The Plant List; scientificName: *Pteris togoensis* Hieron.; kingdom: Plantae; phylum: Monilophyta; class: Polypodiidae; order: Polypodiales; family: Pteridaceae; genus: Pteris; specificEpithet: togoensis; taxonRank: species; scientificNameAuthorship: Hieron.; **Location:** continent: Africa; country: Togo; stateProvince: Plateaux; decimalLatitude: 7.12241675; decimalLongitude: 0.6552094; geodeticDatum: WGS 1984; **Identification:** identifiedBy: K. E. Abotsi; dateIdentified: /05/2013; **Event:** eventDate: 04-09-13; **Record Level:** institutionID: Herbarium togoense; collectionID: TOGO
- **Type status:** Other material. **Occurrence:** catalogNumber: 30080; recordNumber: 271; recordedBy: ABOTSI, SODJINOU & MINGOU; **Taxon:** nameAccordingToID: The Plant List; scientificName: *Pteris togoensis* Hieron.; kingdom: Plantae; phylum: Monilophyta; class: Polypodiidae; order: Polypodiales; family: Pteridaceae; genus: Pteris; specificEpithet: togoensis; taxonRank: species; scientificNameAuthorship: Hieron.; **Location:** continent: Africa; country: Togo; stateProvince: Plateaux; decimalLatitude: 7.11301655; decimalLongitude: 0.6092368; geodeticDatum: WGS 1984; **Identification:** identifiedBy: K. E. Abotsi; dateIdentified: /05/2013; **Event:** eventDate: 04-11-13; **Record Level:** institutionID: Herbarium togoense; collectionID: TOGO
- **Type status:** Other material. **Occurrence:** catalogNumber: 30090; recordNumber: 291; recordedBy: ABOTSI, SODJINOU & MINGOU; **Taxon:** nameAccordingToID: The Plant List; scientificName: *Pteris togoensis* Hieron.; kingdom: Plantae; phylum: Monilophyta; class: Polypodiidae; order: Polypodiales; family: Pteridaceae; genus: Pteris; specificEpithet: togoensis; taxonRank: species; scientificNameAuthorship: Hieron.; **Location:** continent: Africa; country: Togo; stateProvince: Plateaux; decimalLatitude: 6.95445561; decimalLongitude: 0.58201724; geodeticDatum: WGS 1984; **Identification:** identifiedBy: K. E. Abotsi; dateIdentified: /05/2013; **Event:** eventDate: 04-15-13; **Record Level:** institutionID: Herbarium togoense; collectionID: TOGO
- **Type status:** Other material. **Occurrence:** catalogNumber: 30095; recordNumber: 303; recordedBy: ABOTSI, SODJINOU & MINGOU; **Taxon:** nameAccordingToID: The Plant List; scientificName: *Pteris togoensis* Hieron.; kingdom: Plantae; phylum: Monilophyta; class: Polypodiidae; order: Polypodiales; family: Pteridaceae; genus: Pteris; specificEpithet: togoensis; taxonRank: species; scientificNameAuthorship: Hieron.; **Location:** continent: Africa; country: Togo; stateProvince: Plateaux; decimalLatitude: 6.94569087; decimalLongitude: 0.57891444; geodeticDatum: WGS 1984; **Identification:** identifiedBy: K. E. Abotsi; dateIdentified: /05/2013; **Event:** eventDate: 04-15-13; **Record Level:** institutionID: Herbarium togoense; collectionID: TOGO
- **Type status:** Other material. **Occurrence:** catalogNumber: 30099; recordNumber: 309; recordedBy: ABOTSI, SODJINOU & MINGOU; **Taxon:** nameAccordingToID: The Plant List; scientificName: *Pteris togoensis* Hieron.; kingdom: Plantae; phylum: Monilophyta; class: Polypodiidae; order: Polypodiales; family: Pteridaceae; genus: Pteris; specificEpithet: togoensis; taxonRank: species; scientificNameAuthorship: Hieron.; **Location:** continent: Africa; country: Togo; stateProvince: Plateaux; decimalLatitude: 6.85207518; decimalLongitude: 0.7539773; geodeticDatum: WGS 1984; **Identification:** identifiedBy: K. E. Abotsi; dateIdentified: /05/2013; **Event:** eventDate: 04-16-13; **Record Level:** institutionID: Herbarium togoense; collectionID: TOGO

##### Ecological interactions

###### Native status

Native

##### Distribution

Togo (Ecological Zones 2, 3, 4 and 5), Principe Isl., Bioko Isl., Senegal, Guinea, Sierra Leone, Liberia, Ivory Coast, Ghana, Nigeria, Cameroon, Central African Republic, Gabon, Congo, Angola, Sudan, Madagascar, Mali

##### Notes

*Pteris togoensis* is a terrestrial fern, with erect or shortly creeping rhizome. Fronds are scaly, in clumps, often reaching over 1m long (Fig. 17a, b). The petiole, 20 to 45 cm long, is reddish at the extreme base, stramineous above, canaliculate and smooth. The lamiina is bipinnatifid, 20 to 60 cm long; composed by 7-14 pairs of lateral pinnae. The lower pinnae are opposite, 4-5cm spaced, stalked, falcate, forming an angle of 80° with the rachis. The pinnae have a linear-oblong outline, divided almost to the rachis in 20-25 contiguous segments, oblong-obtuse, entire. The tip of the pinnae is linear, entire, of about 2 cm. The first pair of pinnae has a lower pinnatifid auricule. The upper pinnae are subopposite or alternate, sessile. The rachis is stramineous and glabrous. The lower side of lamina carries some yellow glands. The costa is smooth, with a spine on the upper side, at the insertion of the midrib of the lobe. The leaf blade has a sub-coriaceous texture. The ribs are free, usually bifurcated towards the middle. The sori do not reach the top of the lobe, nor the base of the sinus (Fig. 17c, d). *Pteris togoensis* grows almost everywhere in the forests, agro-forests, fallow land, roadsides and river banks.

#### Pteris tripartita

Sw.

Pteris marginata Bory., *Litobrochia tripartita* (Sw.) Presl

##### Materials

- **Type status:** Other material. **Occurrence:** catalogNumber: 12073; recordNumber: 1096; recordedBy: K. Akpagana; lifeStage: Adult; reproductiveCondition: Fertile; **Taxon:** nameAccordingToID: The Plant List; scientificName: *Pteris tripartita* Sw.; kingdom: Plantae; phylum: Monilophyta; class: Polypodiidae; order: Polypodiales; family: Pteridaceae; genus: Pteris; specificEpithet: tripartita; taxonRank: species; scientificNameAuthorship: Sw.; **Location:** continent: Africa; country: Togo; stateProvince: Plateaux; decimalLatitude: 7.5833333; decimalLongitude: 0.6; geodeticDatum: WGS 1984; **Identification:** identifiedBy: K. Akpagana; dateIdentified: /11/1986; **Event:** eventDate: /11/1986; **Record Level:** institutionID: Herbarium togoense; collectionID: TOGO
- **Type status:** Other material. **Occurrence:** catalogNumber: 12074; recordNumber: 1096; recordedBy: K. Akpagana; lifeStage: Adult; reproductiveCondition: Fertile; **Taxon:** nameAccordingToID: The Plant List; scientificName: *Pteris tripartita* Sw.; kingdom: Plantae; phylum: Monilophyta; class: Polypodiidae; order: Polypodiales; family: Pteridaceae; genus: Pteris; specificEpithet: tripartita; taxonRank: species; scientificNameAuthorship: Sw.; **Location:** continent: Africa; country: Togo; stateProvince: Plateaux; decimalLatitude: 7.5833333; decimalLongitude: 0.6; geodeticDatum: WGS 1984; **Identification:** identifiedBy: K. Akpagana; dateIdentified: /11/1986; **Event:** eventDate: /11/1986; **Record Level:** institutionID: Herbarium togoense; collectionID: TOGO
- **Type status:** Other material. **Occurrence:** catalogNumber: 30055; recordNumber: 118; recordedBy: ABOTSI, SODJINOU & MINGOU; **Taxon:** nameAccordingToID: The Plant List; scientificName: *Pteris tripartita* Sw.; kingdom: Plantae; phylum: Monilophyta; class: Polypodiidae; order: Polypodiales; family: Pteridaceae; genus: Pteris; specificEpithet: tripartita; taxonRank: species; scientificNameAuthorship: Sw.; **Location:** continent: Africa; country: Togo; stateProvince: Plateaux; decimalLatitude: 7.58205455; decimalLongitude: 0.62126478; geodeticDatum: WGS 1984; **Identification:** identifiedBy: K. E. Abotsi; dateIdentified: /05/2013; **Event:** eventDate: 04-05-13; **Record Level:** institutionID: Herbarium togoense; collectionID: TOGO

##### Ecological interactions

###### Native status

Native

##### Distribution

Togo (Ecological Zone 4), Australia, Taiwan, China, Singapore, Borneo, S-India, Andaman Isl., Nicobar Isl., Sri Lanka, peninsular Malaysia, Philippines, Thailand, Sulawesi, Sumatra, Laos, Nepal, Vietnam, Java, Moluccas, Uganda, Kenya, Tanzania, Zanzibar, Principe Isl., Sao Tomé, Chagos Arch., Galega Isl., Bioko Isl., Guinea, Sierra Leone, Ivory Coast, Ghana, Benin, Liberia, Nigeria, Cameroon, Central African Republic, Equatorial Guinea, Gabon, Congo, Madagascar, La Réunion, Comores, Seychelles, Mauritius, Angola, Marianas, Fiji, Palau Isl., Micronesia, Marshall Isl., Nauru, Gilbert Isl., Tonga, New Caledonia, American Samoa, Western Samoa, Tuvalu, Society Isl., Austral Isl., Niue, Marquesas Isl., Costa Rica (I), Costa Rica (I), Panama (I), Colombia (I), Venezuela (I), Brazil (I), Ecuador (I), Peru (I), St. Lucia (I), Puerto Rico (I), Jamaica (I), Surinam (I), French Guiana (I), Mexico (I), USA (I)

##### Notes

*Pteris tripartita* is a big fern, terrestrial or epiphytic, with erect, short and thick rhizome, bearing large scales. The fronds are in clumps, 1.5 to 2.5m high (Fig. 18a). The stipe is long (0,3-1,5m), very fleshy and thick. The leaf blade is ovate-deltoid, divided into three equal branches of about 1m long and 25 cm wide. These lateral branches are also divided into similar branches, making the frond pedately shaped. Each branch is bipinnate, with more than 30 pairs of pinnae of (2) 5-25 cm long and 1.3-3 cm wide. The ultimate segments are more or less oblong, straight or falciform, 0.3 to 2 cm long by 3 to 7 mm wide. The ribs form narrow areolae along costae and costulae. They are anastomosed at the ultimate lobes. The leaf blade has a thin texture and a bright green color. Sori extend from sinus to the half of the segment, or almost to the apex (Fig. 18b). This fern usually grows on rich deep soils in tropical rainforests.

## Identification Keys

### Key to the Pteridaceae species of Togo

**Table:** 

1	Aquatic plant, partially or totally submerged	2
–	Terrestrial plant, epiphytic, or lithophyte	3
2	Mangrove plant, lamina coriaceous, oblong-lanceolate, pinnate, lower pinnae short-stalked and terminal pinnae sessile, acrostichoids sori (sporangia covering completely the lower surface of the lamina)	***Acrostichum aureum***
–	Fresh water plant, completely submerged, lamina membranous, lanceolate, fertile frond, 2-4 pinnate, sporangia protected by the reflected margin of lamina	***Ceratopteris thalictroides***
3	Frond entire or flabellate	4
–	Frond pinnate, pinnatifid, multi-pinnate or multi-pinnatifid	5
4	Plant epilithic, lamina fan-shaped, flabellate, coriaceous, stipe sparsely scaled	***Actiniopteris radiata***
–	Epiphytic plant, lamina linear-lanceolate, stipe blackish at the base, marginal sori immersed	**Haplopteris guineensis var. guineensis**
5	Frond pinnate	6
–	Frond multi-pinnate or multi-pinnatifid	9
6	Pinnae long-stalked	***Adiantum lunulatum***
–	Pinnae sessile or sub-sessile	7
7	Pinnae deeply or slightly incised, generally hairy, becoming smaller at the top of the leaf, blade oblong to deltoid, rachis and stipe dark brown, densely hairy	***Adiantum incisum***
–	Pinnae entire or lobed, oblong, oval or lanceolate, glabrous, stipe and rachis black and shiny	8
8	Sori linear, protected by the reflected margin of lamina, terminal pinnae similar to lateral ones, lamina dark green, with coriaceous texture	***Pellaea dura***
–	Sori oblong-kidney shaped, pinnae lobed and serrated feathers on the upper margin, light green leaf blade, papery textured lamina	***Adiantum schweinfurthii***
9	Sori moon crescent shaped, rhomboid-dimidiate pinnae, lamina upper margin incised	***Adiantum vogelii***
–	Sori linear, pinnule rhomboidal, non-dimidiate	10
10	Stipe black	11
–	Stipe not black	13
11	Limb oblong-lanceolate, not winged, covered by a white powdery layer on the underside	**Pityrogramma calomelanos var. calomelanos**
–	Limb deltoid, broadly winged, 3-branched at the base, sori marginal	12
12	Sori linear, interrupted	***Doryopteris kirkii***
–	Sori linear, continuous over the entire margin of the lamina, except at the base of sinus	**Doryopteris concolor var. nicklesii**
13	Limb oval-deltoid light green, 3-pinnate, equal pinnae, lamina herbaceous, stipe tri-branched at the base of the limb	***Pteris tripartita***
–	Limb lanceolate to deltoid, oblong, elliptic or oval, basal pinnae developed basiscopically, stipe not branched	14
14	Sori linear or not reaching the bottom of the base or the apex of the pinnae, stipe reddish at the base	***Pteris togoensis***
–	Sori U-shaped, interrupted at the top of pinnae	15
15	Frond deltoid, more or less lanceolate, brown stipe, simple or pinnatifid pinnae, both types can coexist on the same frond, oblong-lanceolate	***Pteris burtonii***
–	Frond not deltoid, stipe straw colored, pinnatifid pinnae only	16
16	Stipe channeled, muricate, purplish or brownish at the base, pinnae and pinnule oblong-lanceolate, lamina proliferous	***Pteris similis***
–	Stipe reddish at the base, pinnae oblong, pinnule oblong-falcate, limb non-proliferous	***Pteris atrovirens***

## Analysis

**Figure 2.**
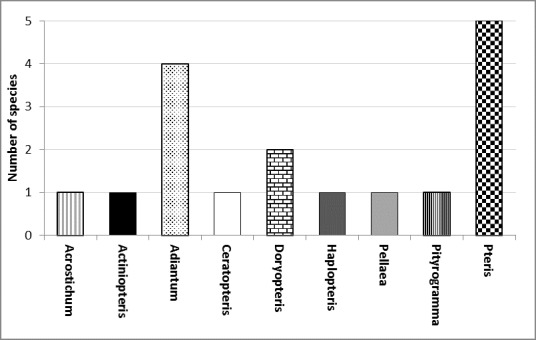
Species diversity by gender within Pteridaceae of Togo (Suppl. material 1).

From all the studied specimens, 17 species have been identified, belonging to the Pteridaceae family. They are distributed into 9 genera which are themselves distributed into 4 subfamilies (Fig. 2). The Cryptogrammoideae subfamily is not present in the study area. The dominant genera are *Pteris* L. (5 species) and *Adiantum* L. (4 species). The subfamilies of Ceratopteridoideae, Cheilanthoideae and Vittarioideae are respectively represented by 2 genera. Furthermore, the Pteridoideae subfamily is represented by 3 genera (Fig. 2).

**Figure 3.**
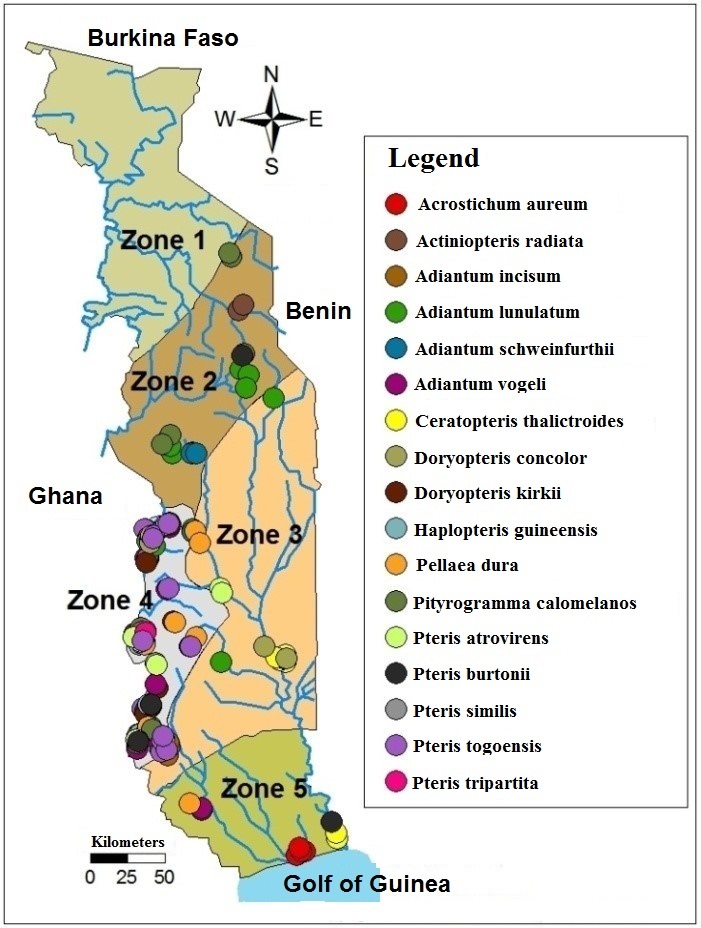
Distribution of Pteridaceae in Togo. Zones 1 to 5 indicated on the map correspond to the ecological zones of Togo as defined by Ern (1979).

**Figure 10.**
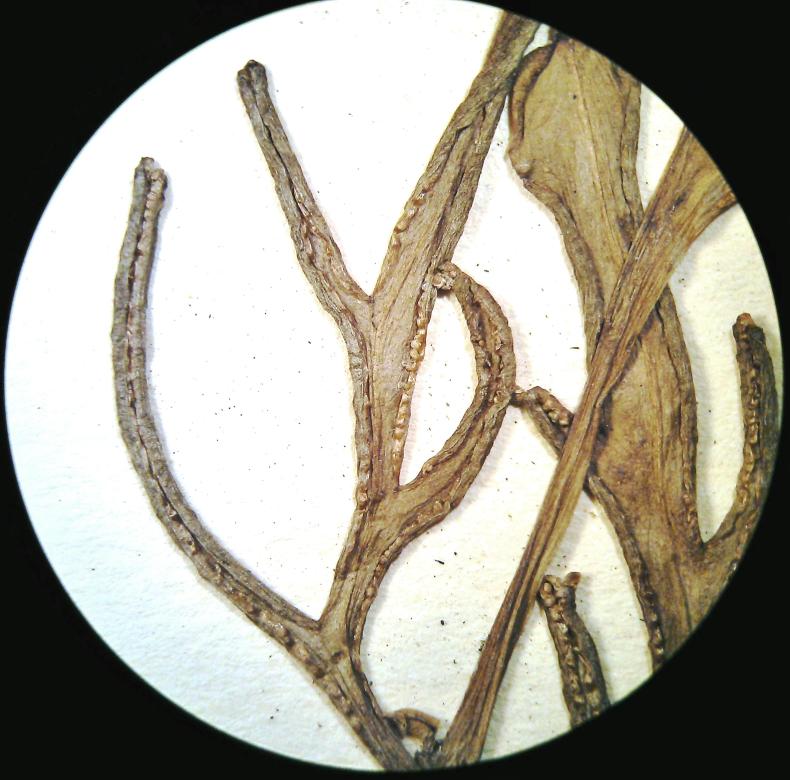
*Ceratopteris thalictroides*: fertile frond.

*Pteris similis* Kuhn was harvested in a riparian forest at Dikpéléou in Adélé region (ASM 0162). This collection confirms the presence of this undocumented species in Togo. The complete list of taxa, their synonyms and their distribution in Togo are indicated in the checklist. The geographic data on the family helped to map species distribution for the whole country (Fig. 3).

The recognition and distinction of taxa within the Pteridaceae species of Togo is based on their habitat, the shape and divisions of their limb, the organization of sporangia in sori, their shape and position. The nature and position of coverings, and exudates are also very decisive. However, the dimensions of the sporophyte are less crucial than the previous characters.

## Discussion

Most of recorded species (14 species of 17 in total) are represented in the ecological zone 4. Some among them are unique to this area (*Adiantum* incisum, *Doryopteris kirkii*, Haplopteris guineensis var. guineensis, *Pteris atrovirens*, *Pteris similis* and *Pteris tripartita*). On the other side, *Actiniopteris radiata* and *Acrostichum aureum* are respectively exclusive to the ecological zones 2 and 5. The entire chain of the mountains of Togo is rich: 16 species out of 17. This distribution shows the role played by biodiversity refuges such as mountains (Moran 1995), which are then comparable to islands. These results also confirm works of Aldasoro et al. (2004) on the biogeography of ferns in sub-Saharan Africa which show that ferns prefer mountainous and altitudinal areas.

Three families in the RIHA database of the herbarium of Lomé correspond to the current delimitation of the Pteridaceae (Christenhusz et al. 2011). These are the former families of "Adiantaceae", "Vittariaceae" and "Pteridaceae". They are now treated within a single taxon: the Pteridaceae family. About 84% (16 names on 19) of the species names in the RIHA database match the current delimitation of the Pteridaceae. However, two other species of the genus *Nephrolepis* are currently integrated within the Nephrolepidaceae (Hennequin et al. 2010). The name *Pellaea doniniana* Link. does not correspond to any described taxon (The Plant List 2015). Among the 16 names corresponding currently to the Pteridaceae, 6 are synonyms (Table 2). Moreover, *Vittaria guineensis* Desv. belongs henceforth to the genus *Haplopteris* but remains a Pteridaceae genus (Crane 1997). Finally, *Pteris similis* Kuhn, harvested in the ecological zone 4 is not present in the collections of Lomé and Paris, referents herbaria of this study, nor in GBIF and RIHA databases. Indeed, this species was reported in Togo by Roux (2001) without reference to samples consulted for this purpose.

Togo ecological zone 4 concentrates 94.12 % of the species diversity of Togolese Pteridaceae. Mountain forests of this area seem like refuges for biodiversity (Aldasoro et al. 2004). The distribution of Pteridaceae in Togo then corroborates works of Moran (1995), highlighting the close link which exists between the presence of mountain ranges, humidity, shade and diversity of ferns of an area. In Togo, rainforest of the ecological zone 4 constitute the ideal habitat for the development of Pteridaceae but unfortunately, reforestation efforts undertaken since 1908 goes mainly through the establishment of extensive plantations of teak (*Tectona grandis*), an exotic plant (MERF 2009). This gradual replacement of natural ecosystems by monoculture plantations pushed back the natural habitat of ferns in general and of Pteridaceae in particular. Indeed, from all the plantations covered, only *Adiantum lunulatum* could be harvested in young teak plantations, old plantations being devoid of all plant species.

The expansion of croplands is also a real threat to the Pteridaceae diversity. Indeed, with the expansion of agriculture added to the effect of bushfires at the detriment of the natural ecosystems (MERF 2011), Togo may lose much of its diversity, whereby the *Pteris similis* Kuhn is a species which distribution in Togo is not yet well known. Inadequacy of policies of reforestation with the conservation of the biodiversity, and the lack of specialists and systematic studies on some groups, including ferns, result in the abandonment of the systematic component in favor of a macro-ecological and especially economic components.

Similarly, the current prerogatives on the carbon stock promote species that store more carbon without necessarily referring to the local flora. Therefore, timber plantations like *Tectona grandis*, *Eucalyptus* spp., *Gmelina arborea*, *Nauclea diderrichii* and *Senna siamea* which are mostly exotic are encouraged (MERF 2009) for their ability to store more carbon and/or for their economic value. The impact of these exotic species on the biodiversity of natural developments which they replace is often minimized. At the pace where deforestation is increasing in Togo (20,000 hectares per year according to FAO 2011), the Pteridaceae might disappear at middle term from the local flora due to loss of habitat.

### Conclusion

This study clarifies the nomenclature and systematic boundaries of the Pteridaceae in Togo. The Pteridaceae accounts for 20 % of the species diversity and 23.7% of the generic diversity of Monilophytes in Togo. It comprises a variety of 17 species included into 9 genera. Major genera are *Pteris* L. and *Adiantum* L. with respectively 5 and 4 species for each. Despite its relatively high diversity (the highest among ferns in Togo), the Pteridaceae are not distributed uniformly over the study area. Approximately 94.12 % of the species are confined in the ecological zone 4 which enjoys a tropical climate and is covered by a semi-deciduous evergreen forest. The zone is like a refuge for biodiversity. Few species are still subservient to the ecological zone 2 (*Actiniopteris radiata*) or 5 (*Acrostichum aureum*).

This study is a basis for future valorization of the family. Considerations should be given to *ex-situ* conservation as well as restoration and conservation of some natural degraded areas to conserve the diversity of ferns and other Pteridaceae in Togo. Further studies extended to the whole West African sub-region would be needed for a better knowledge of the Pteridaceae.

## Supplementary Material

Supplementary material 1 Number of Pteridaceae species by gender in Togo Data type: occurences
File: oo_41787.xls ABOTSI Komla Elikplim

XML Treatment for Acrostichum aureum

XML Treatment for Actiniopteris radiata

XML Treatment for Adiantum incisum

XML Treatment for Adiantum lunulatum

XML Treatment for Adiantum schweinfurthii

XML Treatment for Adiantum vogelii

XML Treatment for Ceratopteris thalictroides

XML Treatment for Doryopteris concolorvar.nicklesii

XML Treatment for Doryopteris kirkii

XML Treatment for Haplopteris guineensisvar.guineensis

XML Treatment for Pellaea dura

XML Treatment for Pityrogramma calomelanosvar.calomelanos

XML Treatment for Pteris atrovirens

XML Treatment for Pteris burtonii

XML Treatment for Pteris similis

XML Treatment for Pteris togoensis

XML Treatment for Pteris tripartita

## Figures and Tables

**Figure 4a.**
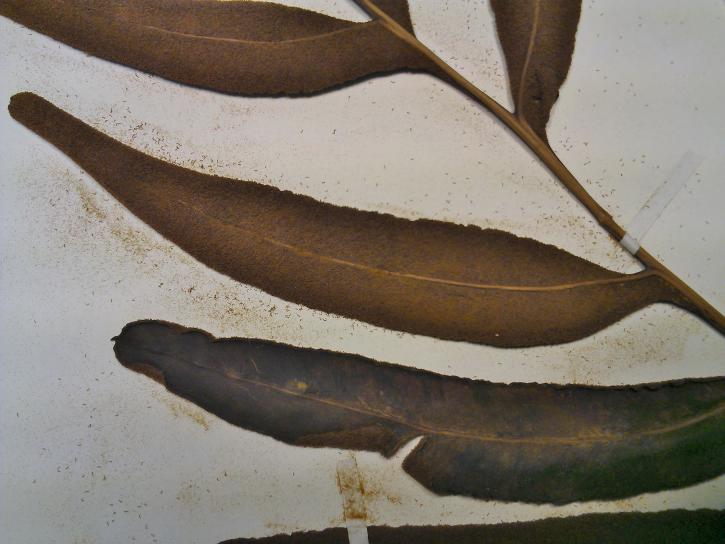
General aspect of pinnae

**Figure 4b.**
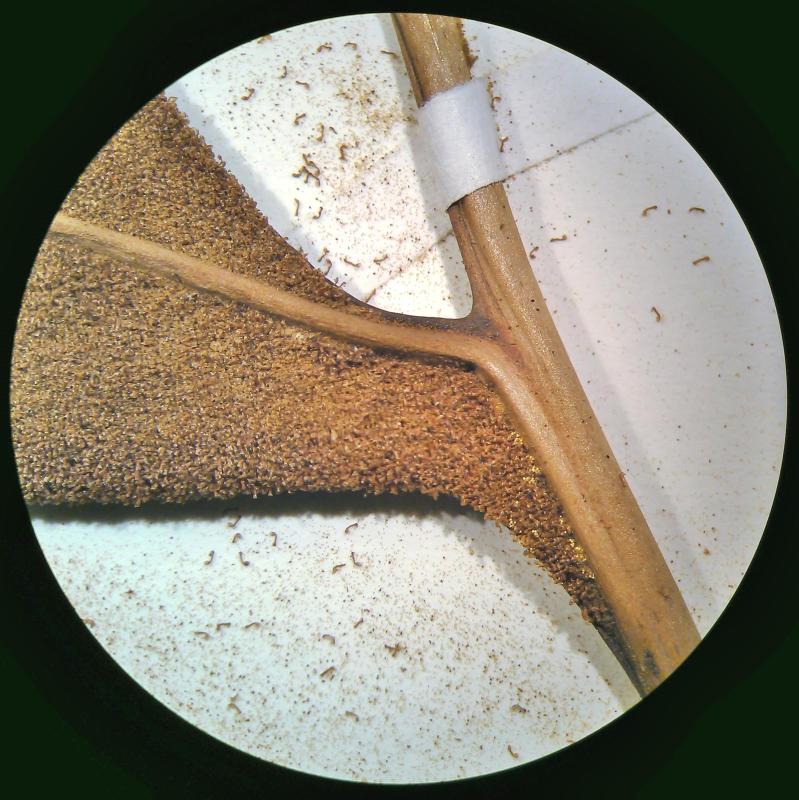
Sporangia on the underside of pinnae

**Figure 5a.**
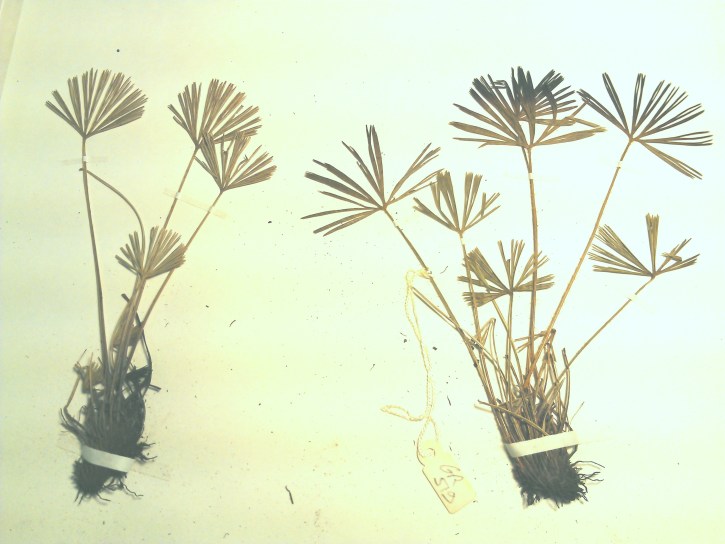
General aspect of the fern

**Figure 5b.**
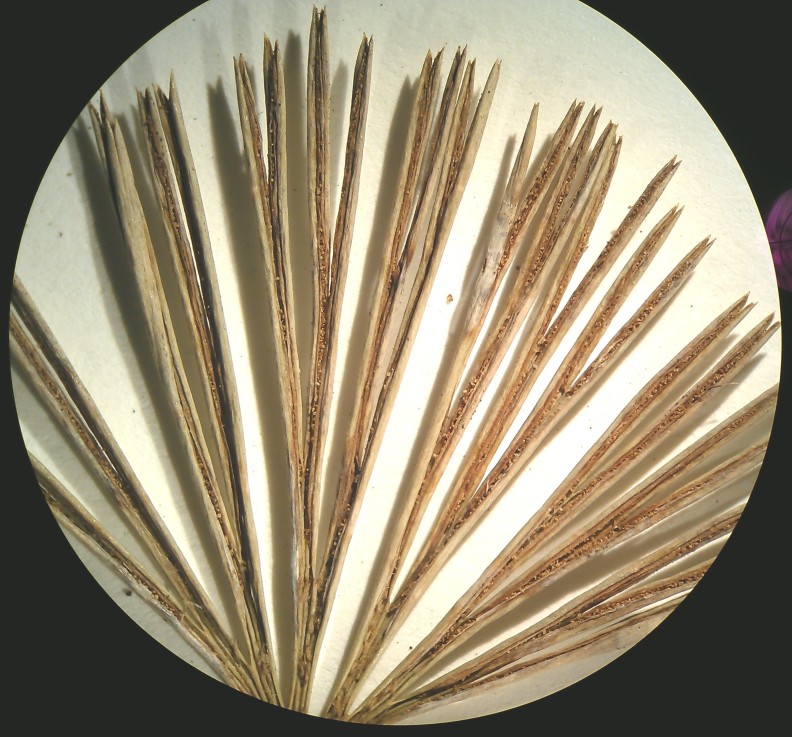
Details of the lamina

**Figure 6a.**
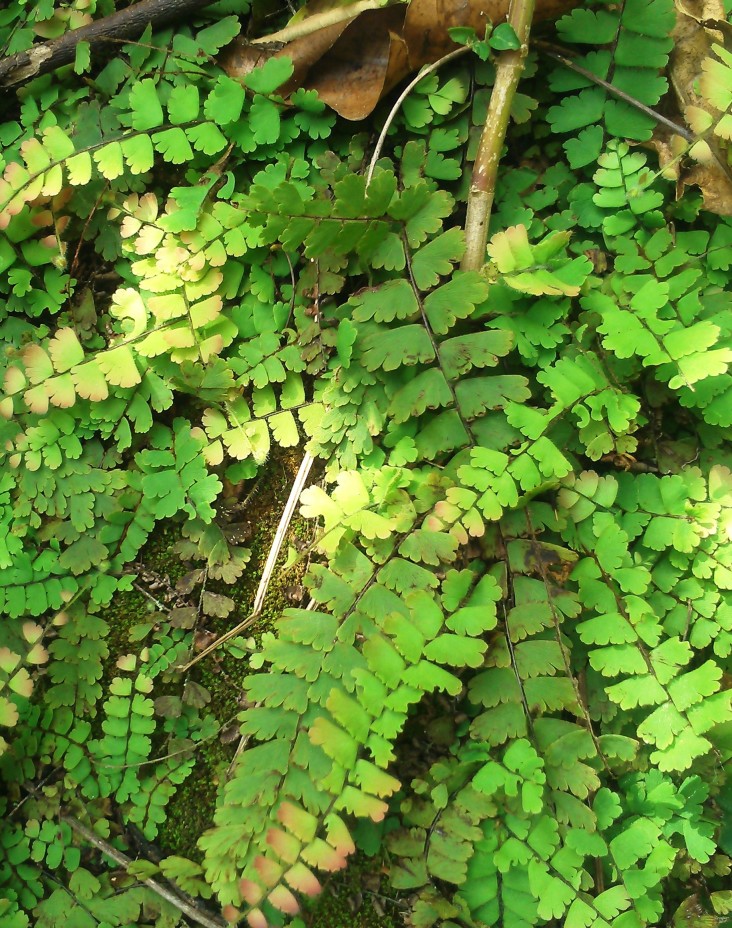
Entire fronds

**Figure 6b.**
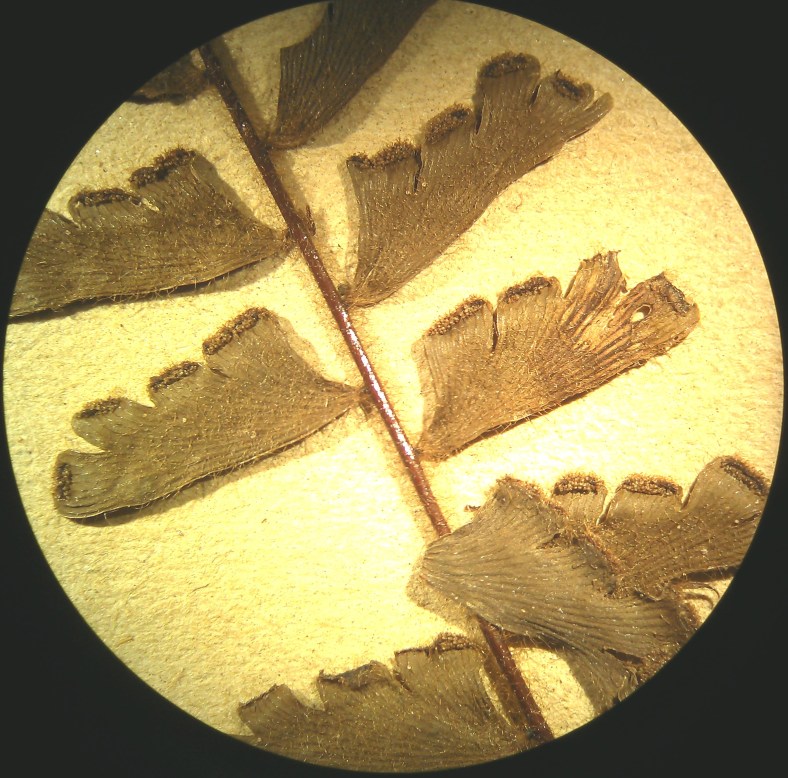
Pinnae

**Figure 6c.**
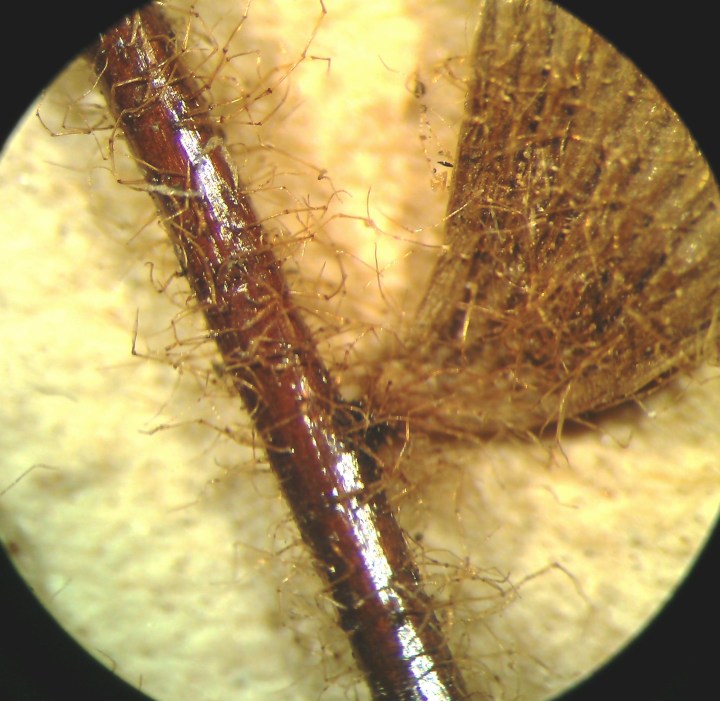
Hairs on the stipe and pinnae

**Figure 6d.**
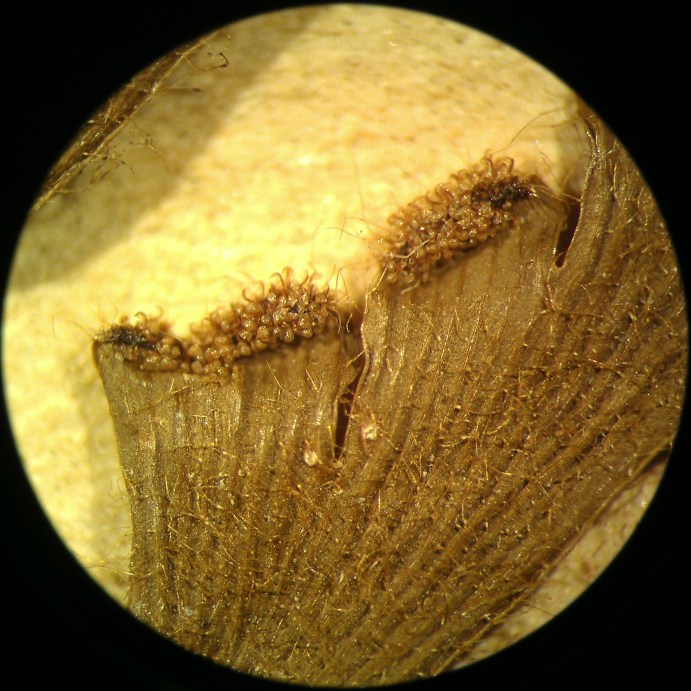
Sori

**Figure 7a.**
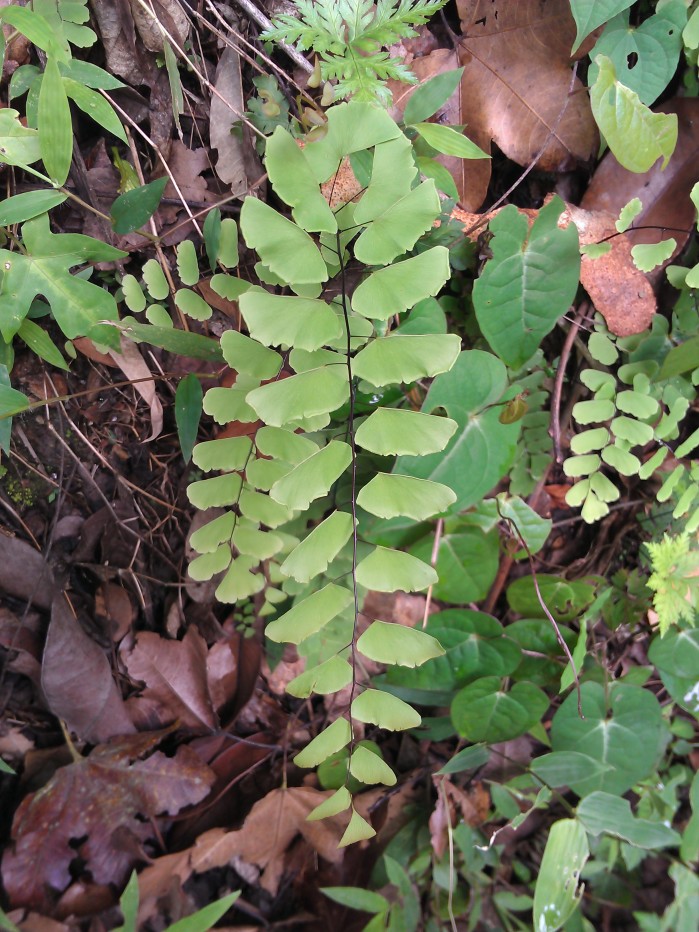
Entire frond

**Figure 7b.**
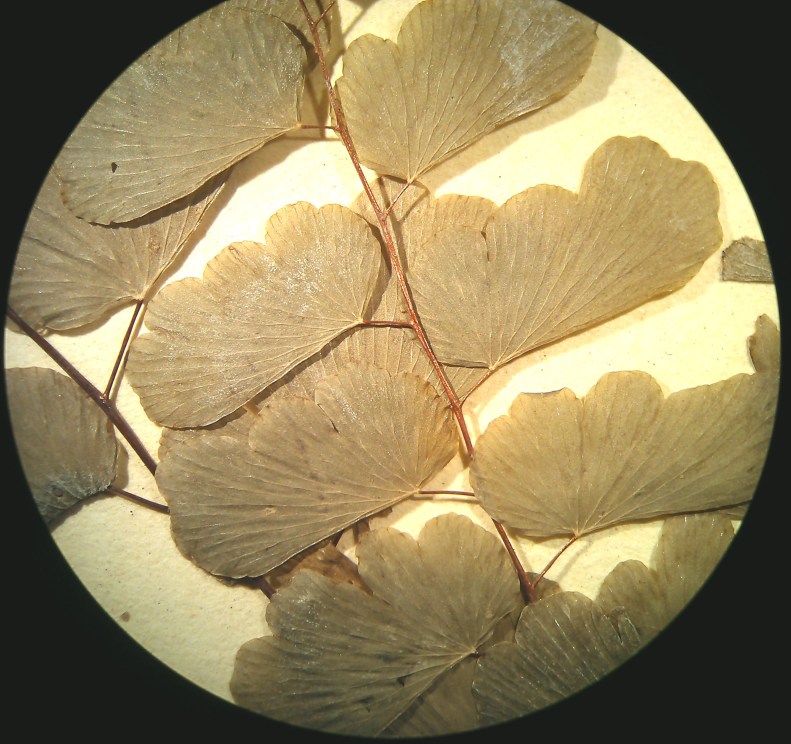
Lateral pinnae

**Figure 7c.**
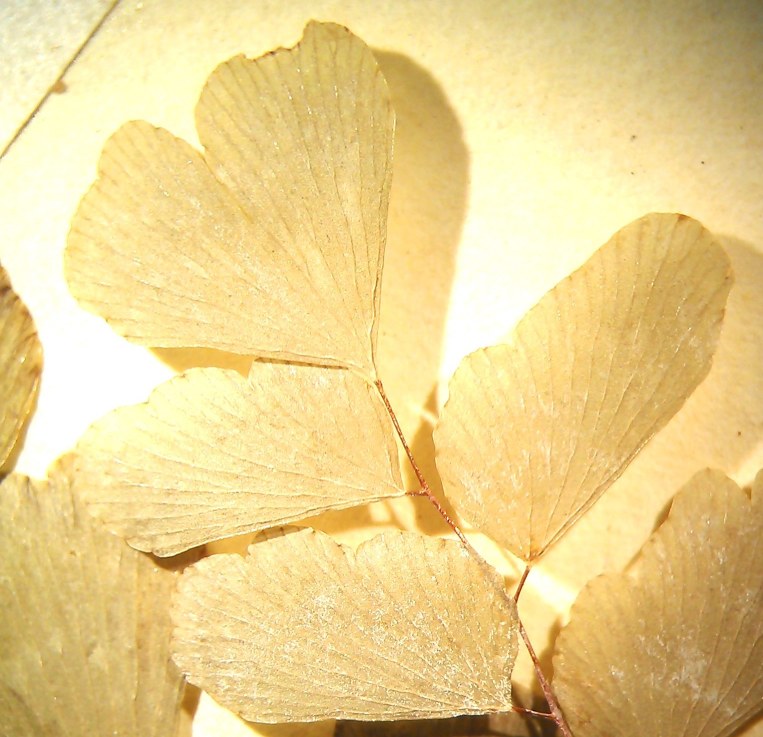
Terminal pinnae

**Figure 7d.**
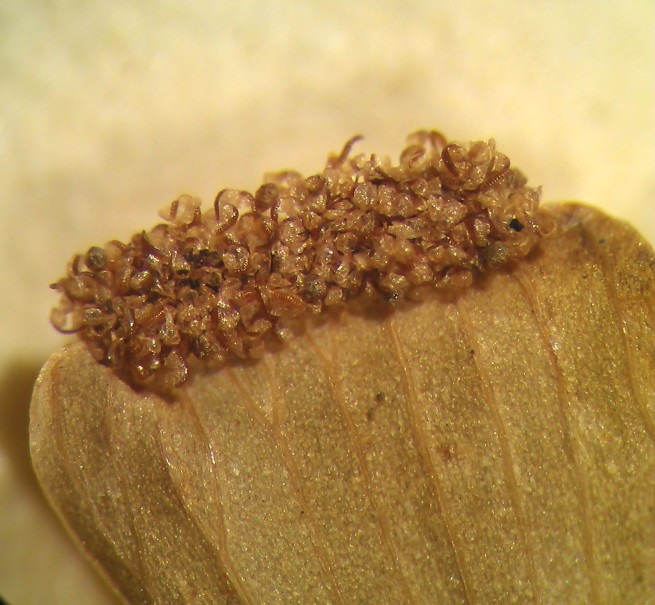
Sori

**Figure 8a.**
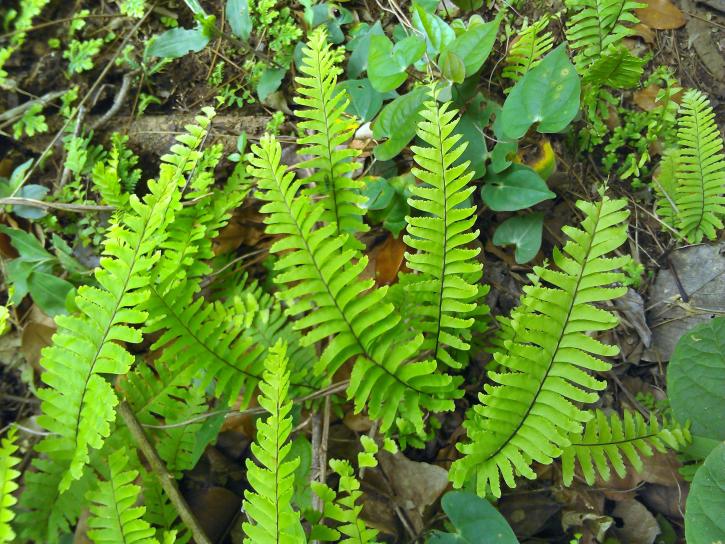
Entire fronds

**Figure 8b.**
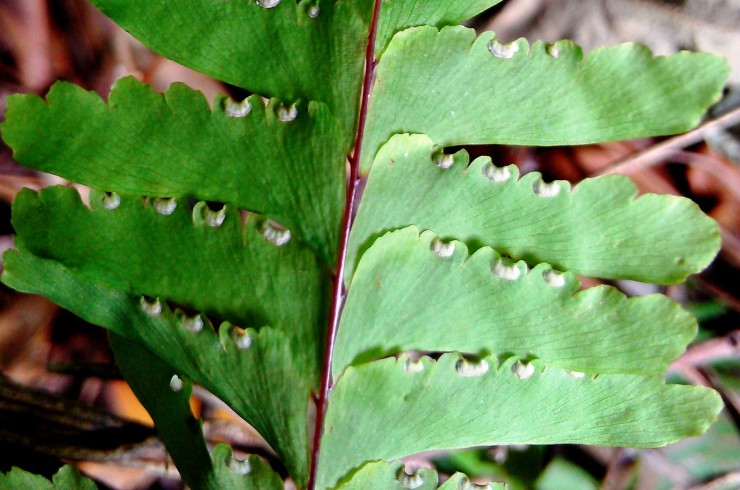
Pinnae showing sori on the upper margin

**Figure 9a.**
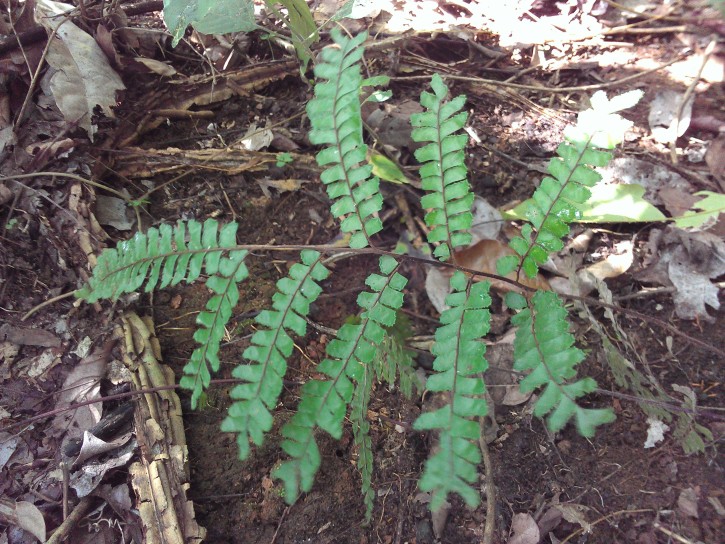
Entire frond

**Figure 9b.**
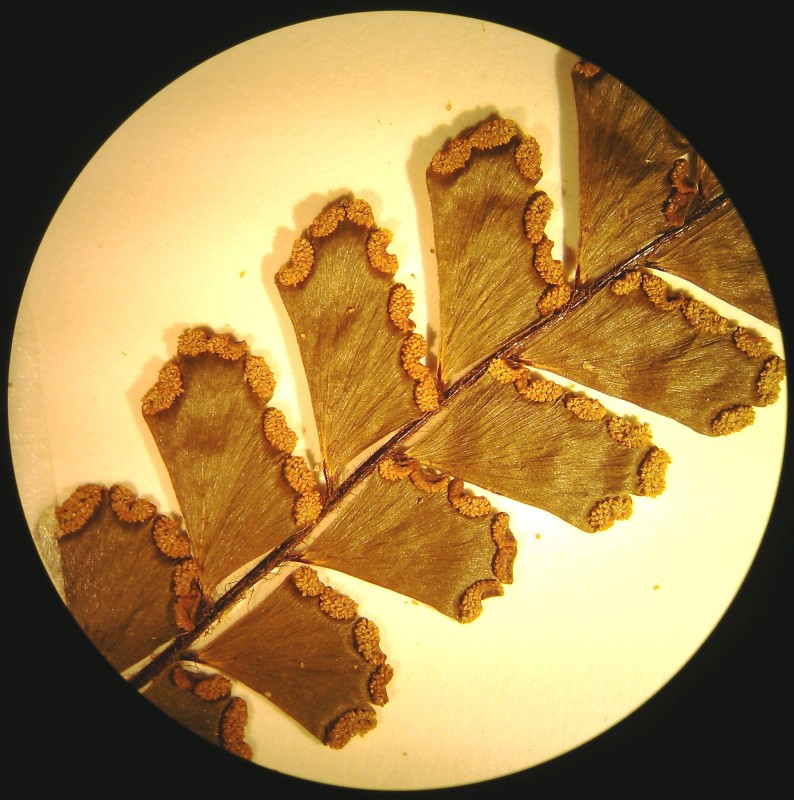
Details of pinnae and sori

**Figure 11a.**
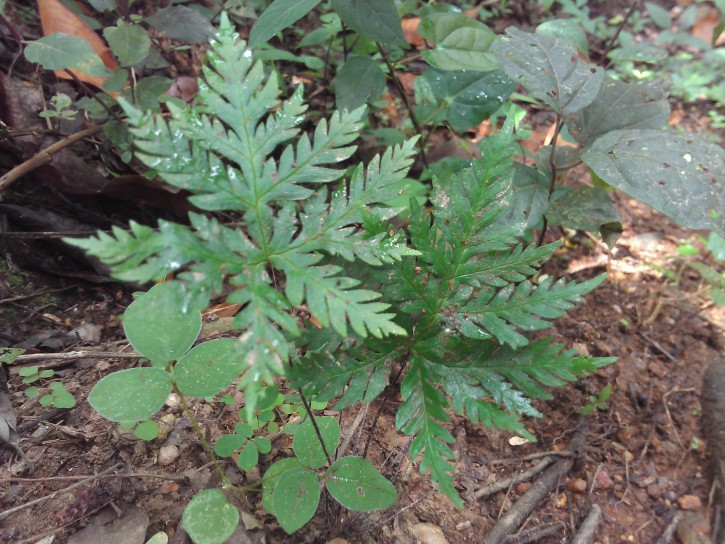
General aspect of the fern

**Figure 11b.**
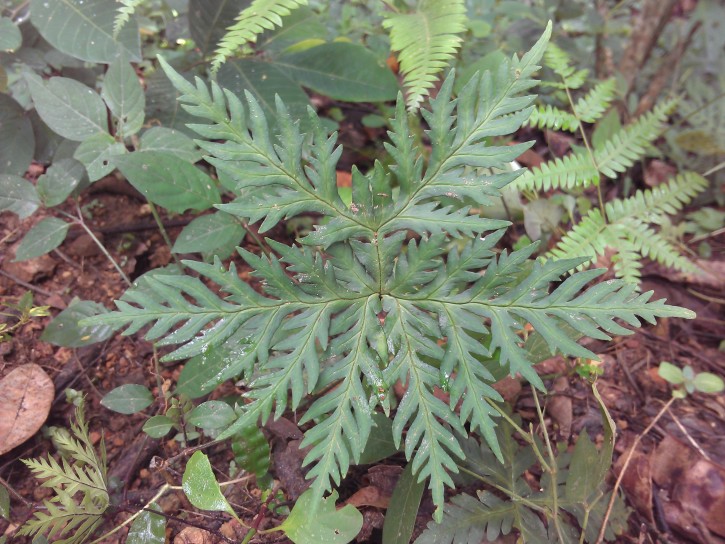
Shape of the lamina

**Figure 11c.**
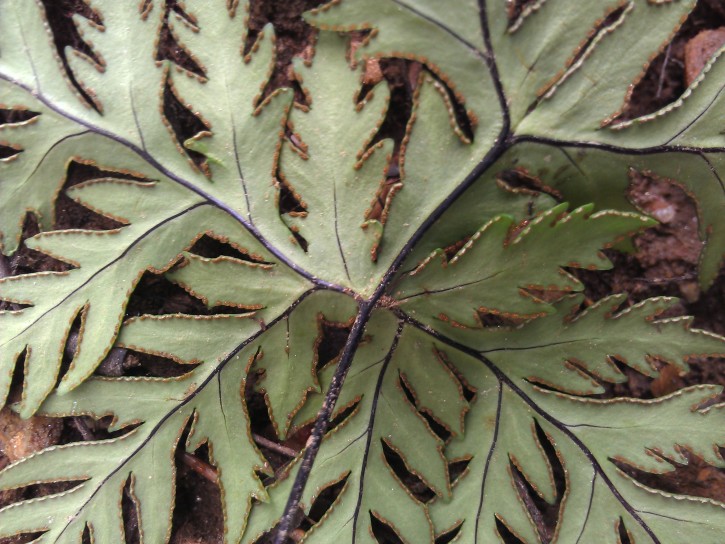
Underside of a fertile frond

**Figure 11d.**
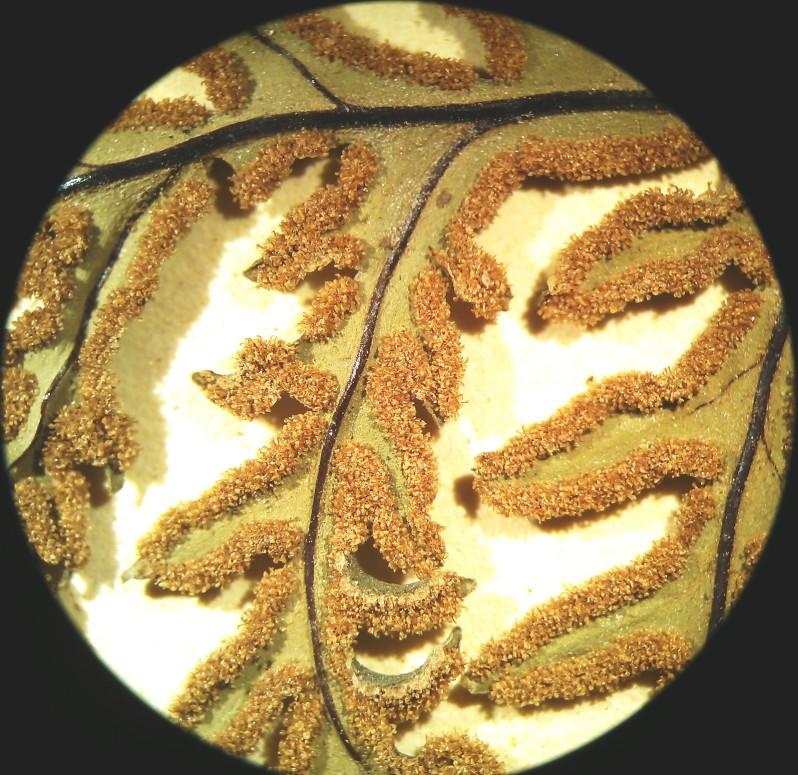
Details of sori

**Figure 12a.**
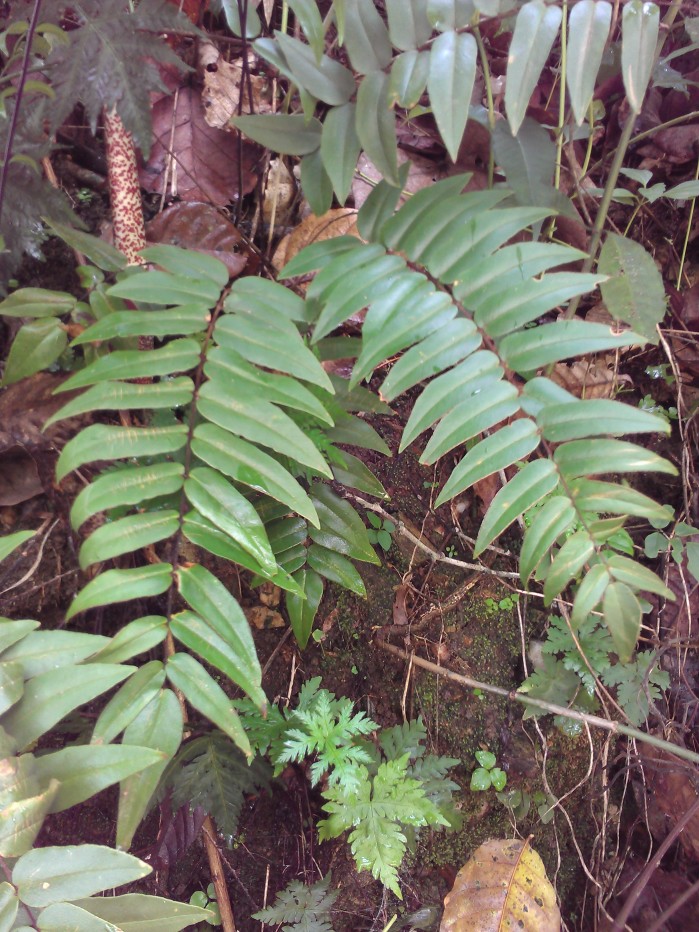
General aspect of the fern (upper face)

**Figure 12b.**
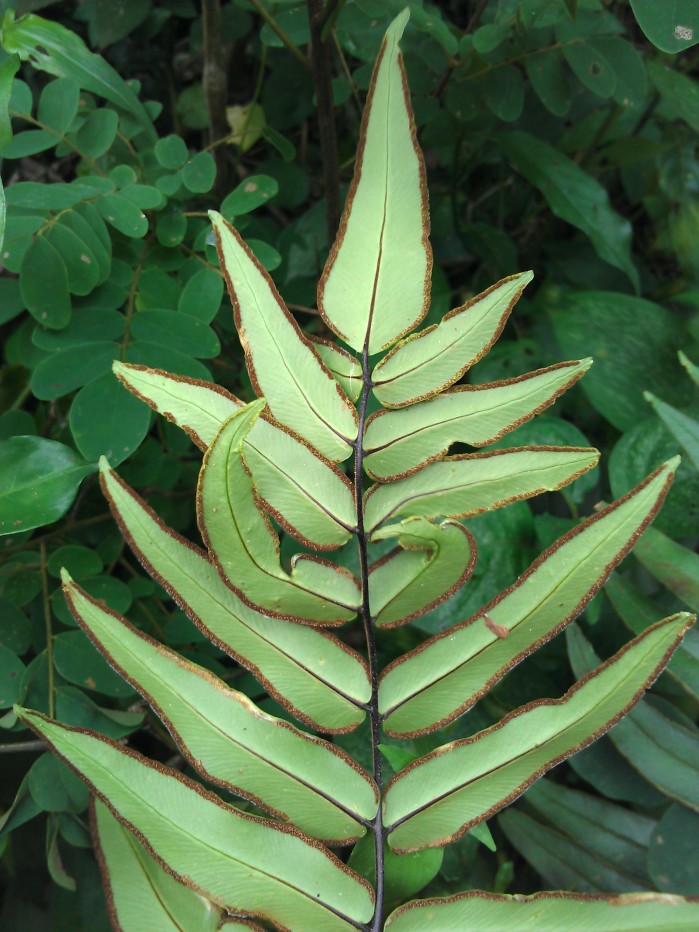
Lower face of a fertile frond

**Figure 12c.**
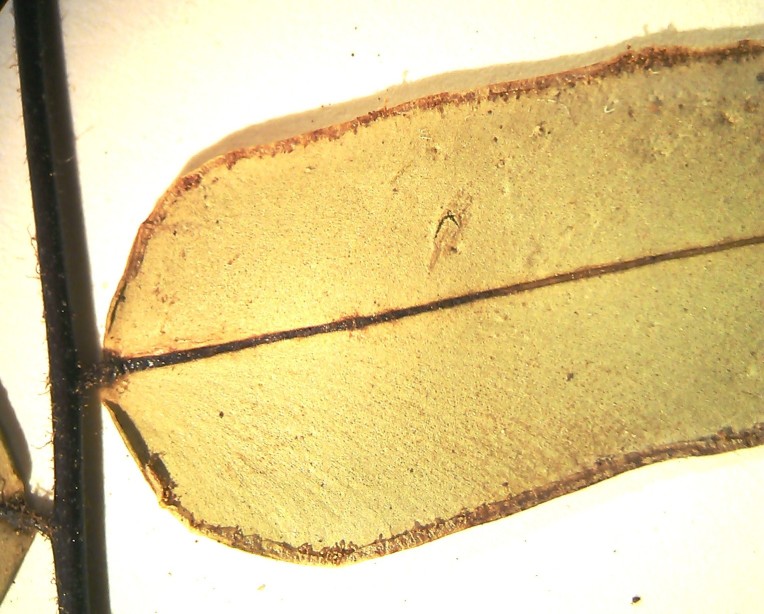
Insertion of a fertile pinnae of the rachis: overview of the false indusium

**Figure 12d.**
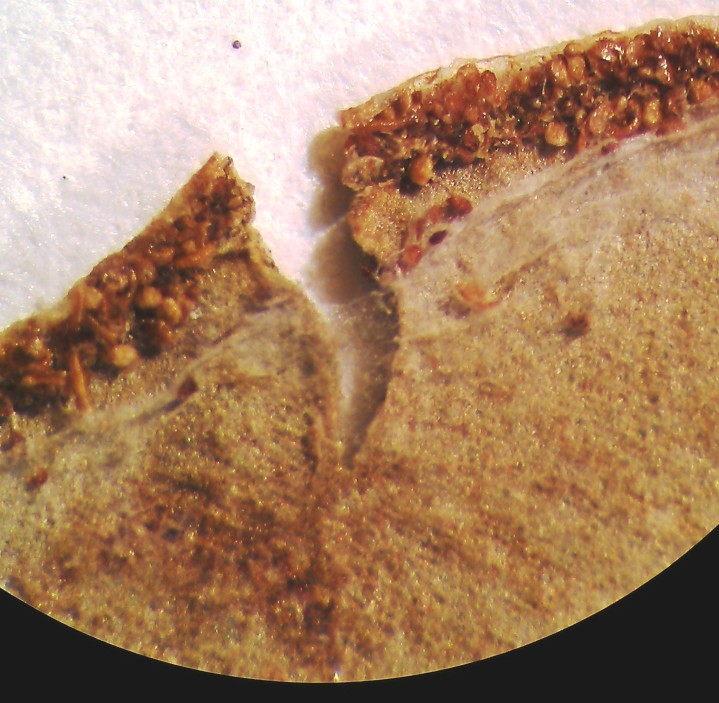
Sori

**Figure 13a.**
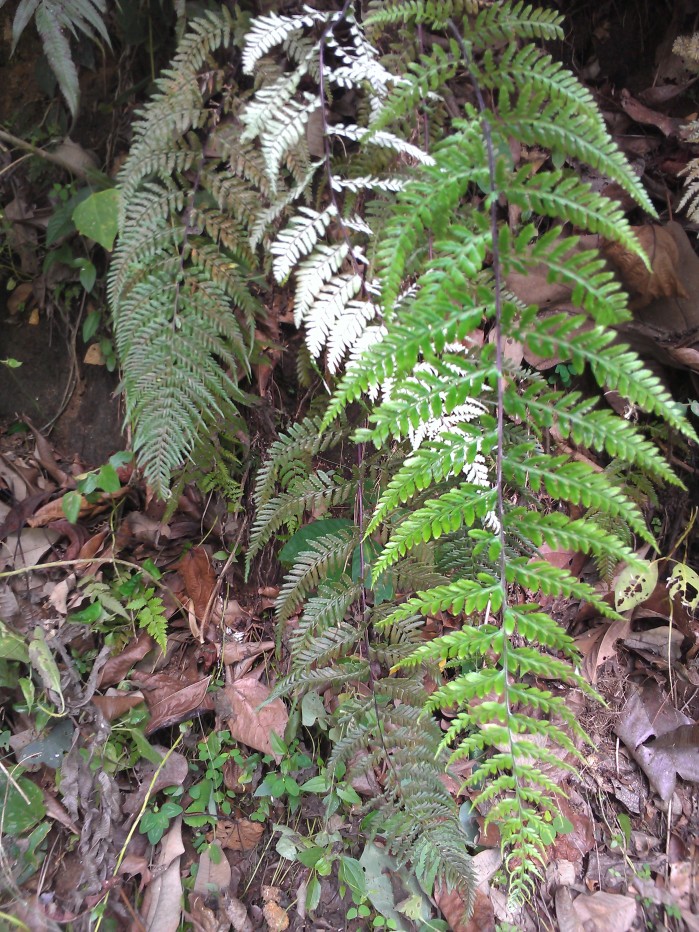
Upper face of the frond

**Figure 13b.**
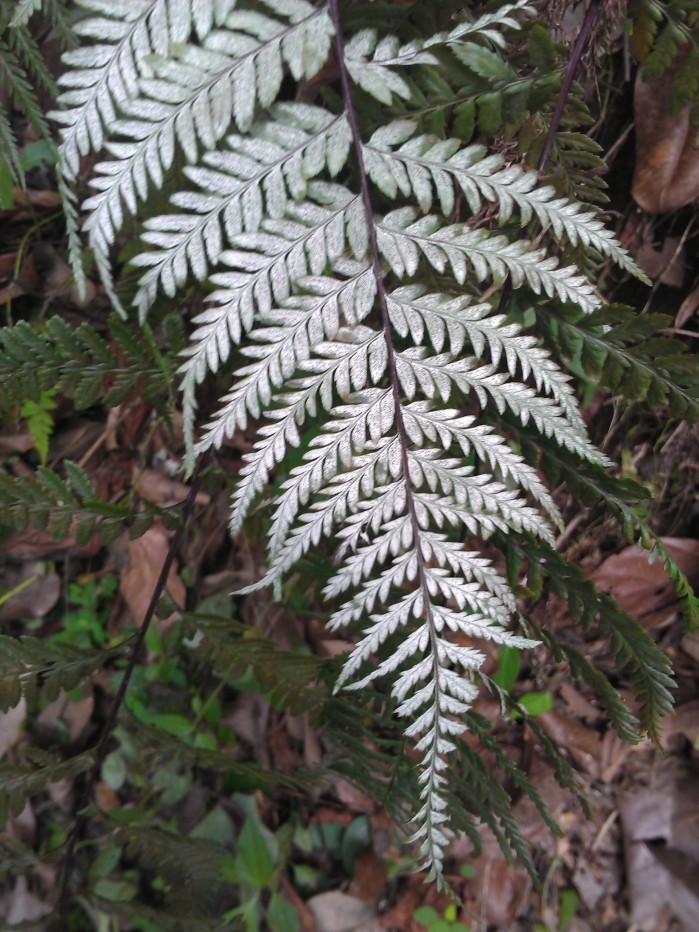
Lower face of the frond

**Figure 14a.**
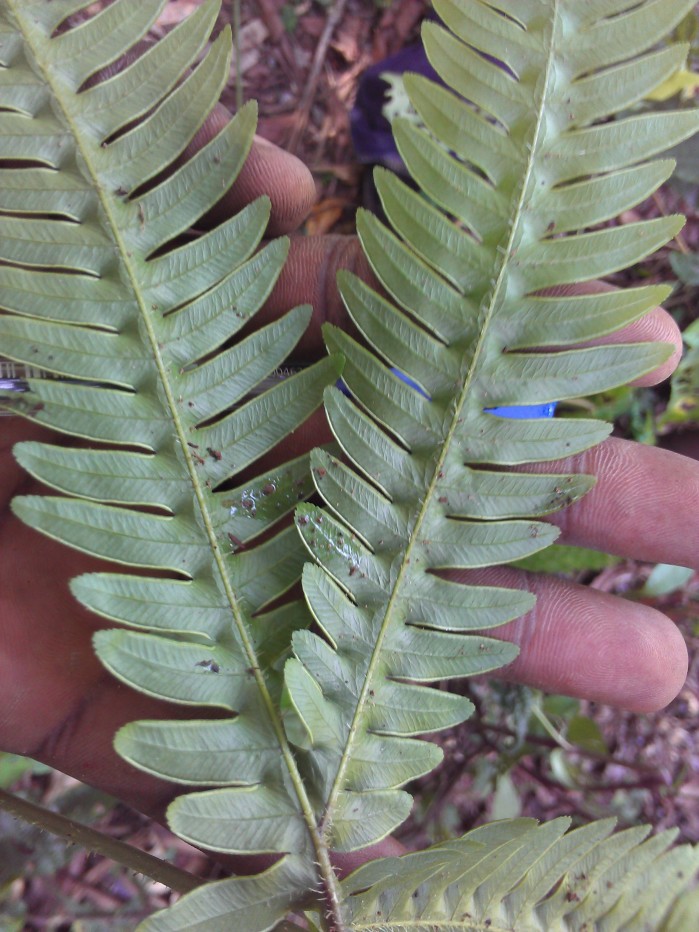
Pinnae

**Figure 14b.**
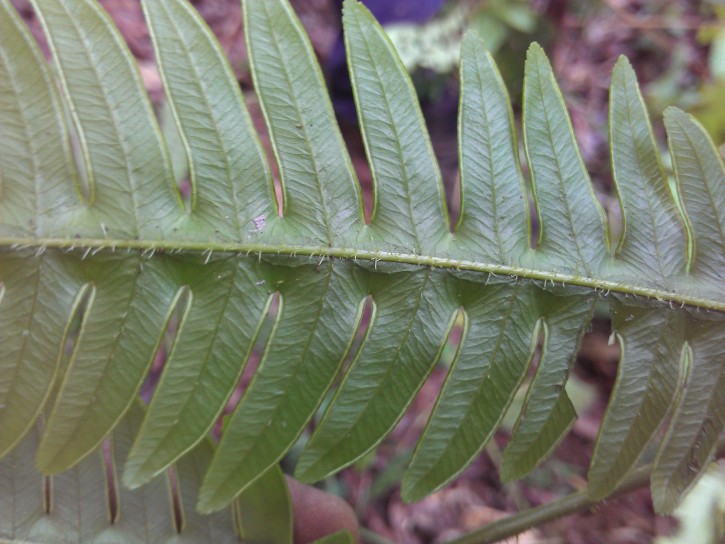
Pinnules

**Figure 15a.**
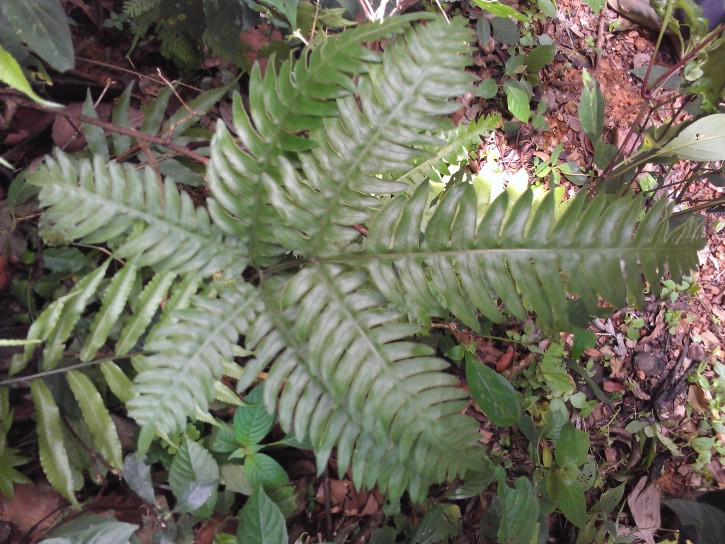
General aspect of the frond (upper face)

**Figure 15b.**
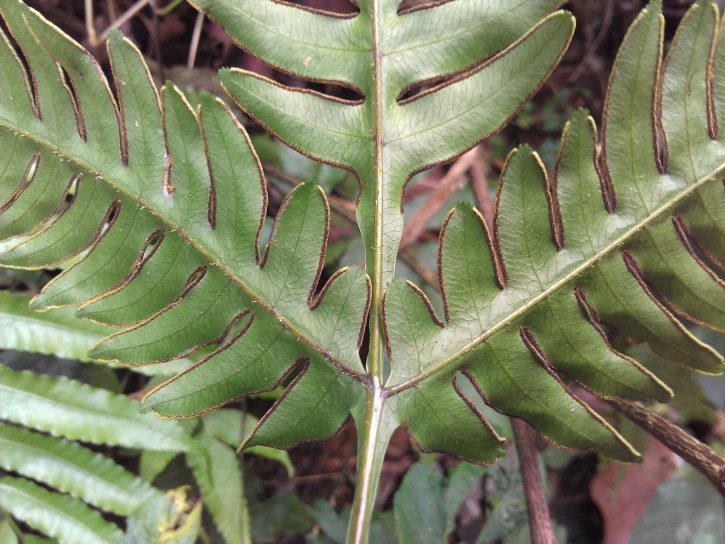
Lower face of the lamina, showing sori

**Figure 16a.**
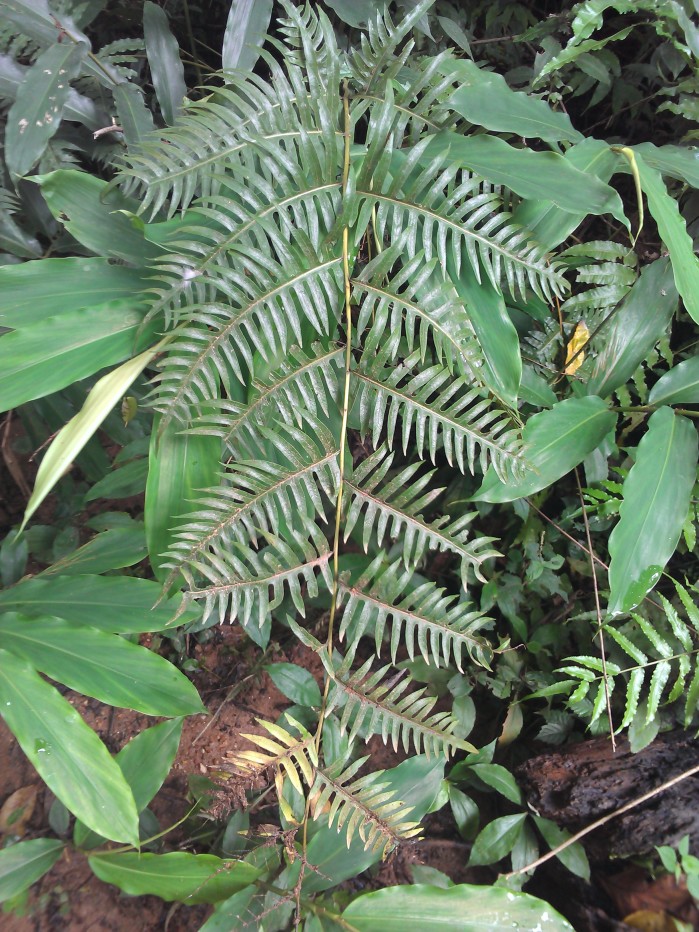
General aspect of the frond

**Figure 16b.**
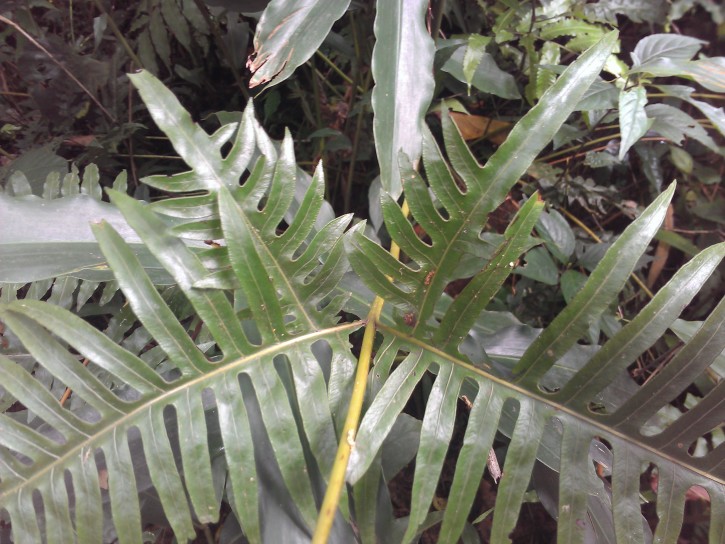
Base of the lamina showing basal spur

**Figure 16c.**
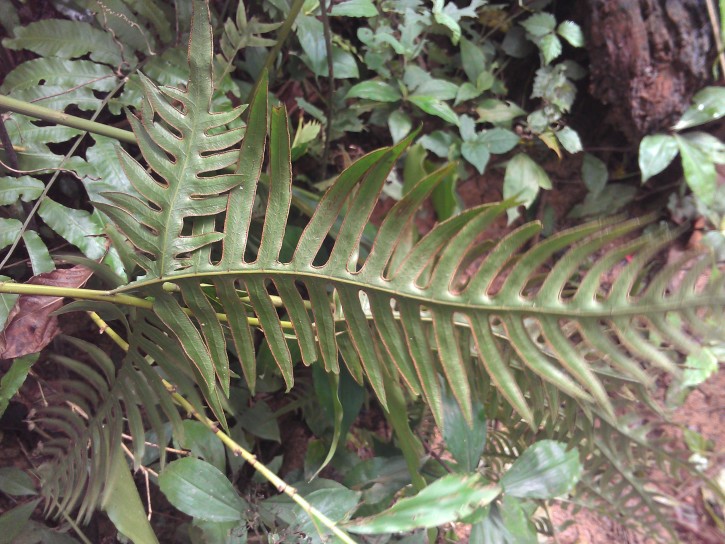
Lower face of the frond

**Figure 16d.**
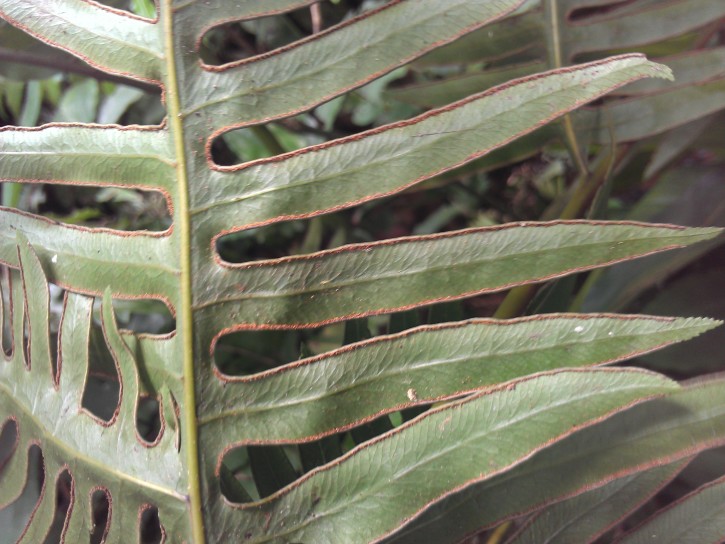
Sori on pinnules

**Figure 17a.**
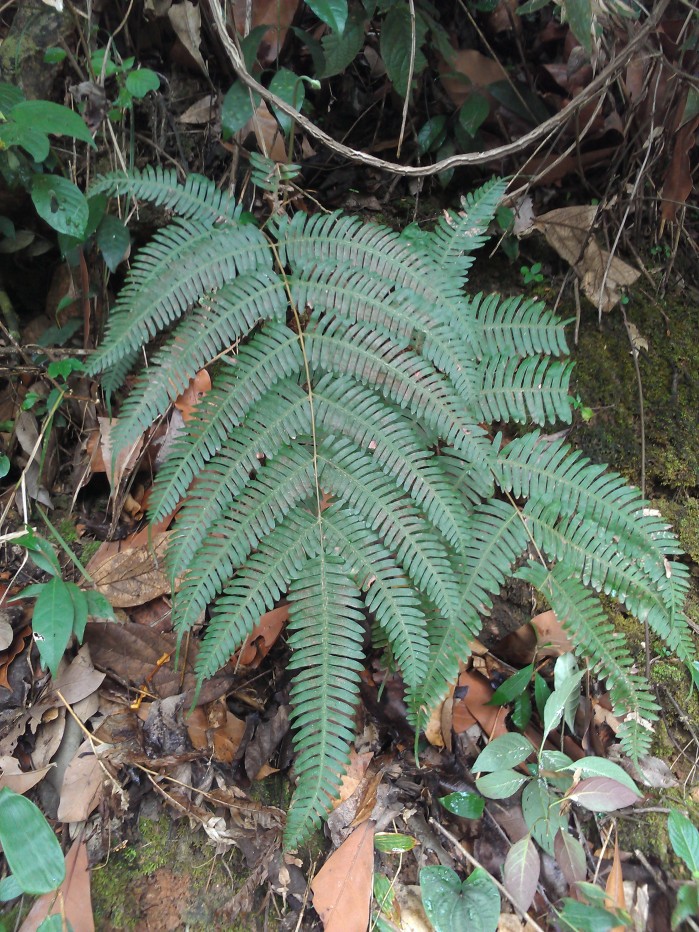
General aspect of the fern (upper face)

**Figure 17b.**
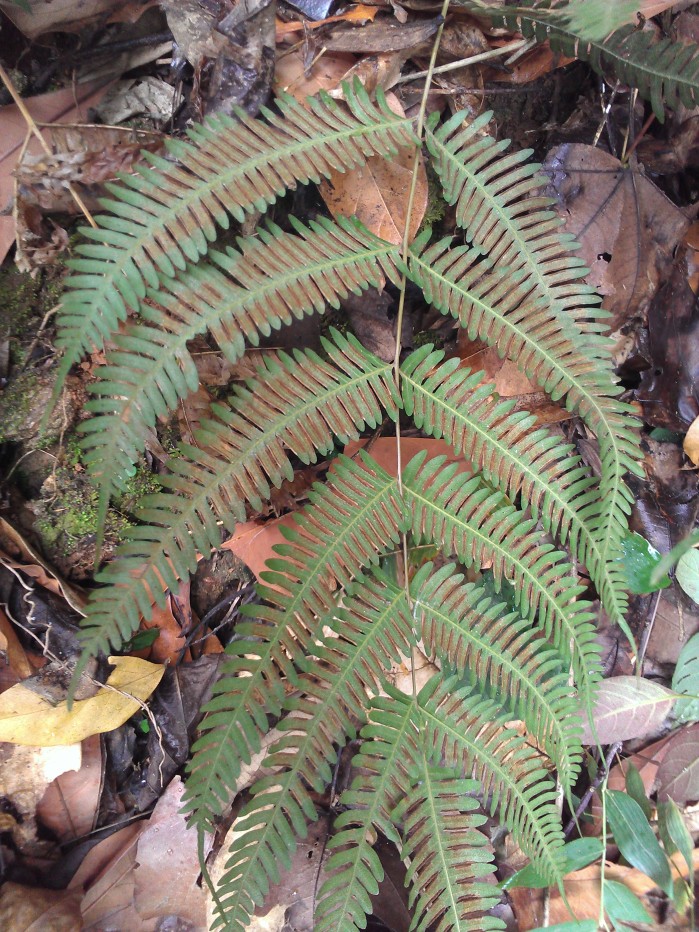
General aspect of the fern (lower face)

**Figure 17c.**
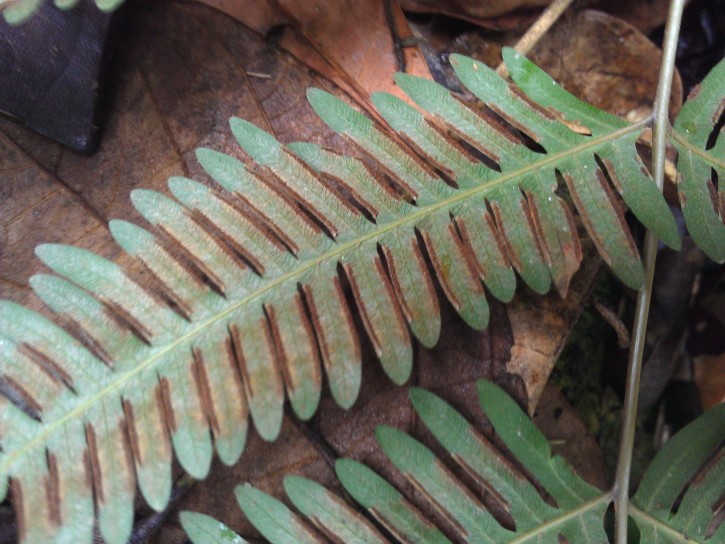
Lower face of a pinnae showing linear sori

**Figure 17d.**
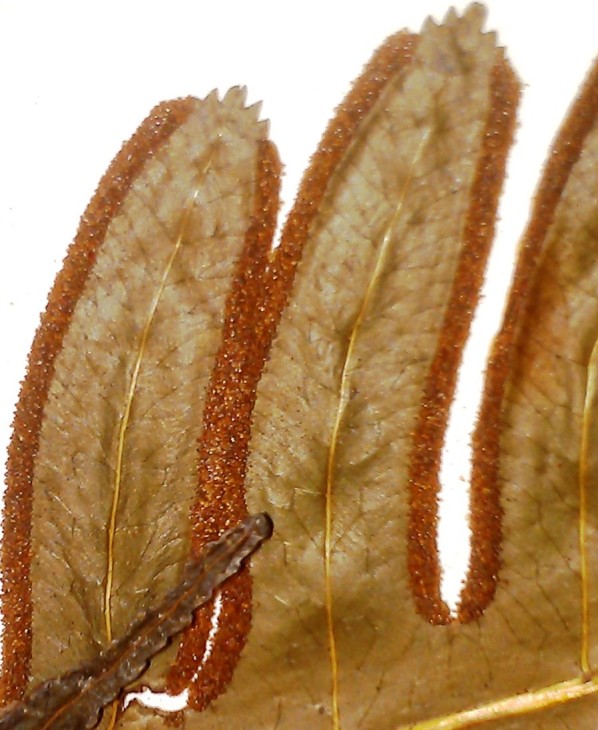
Sori

**Figure 18a.**
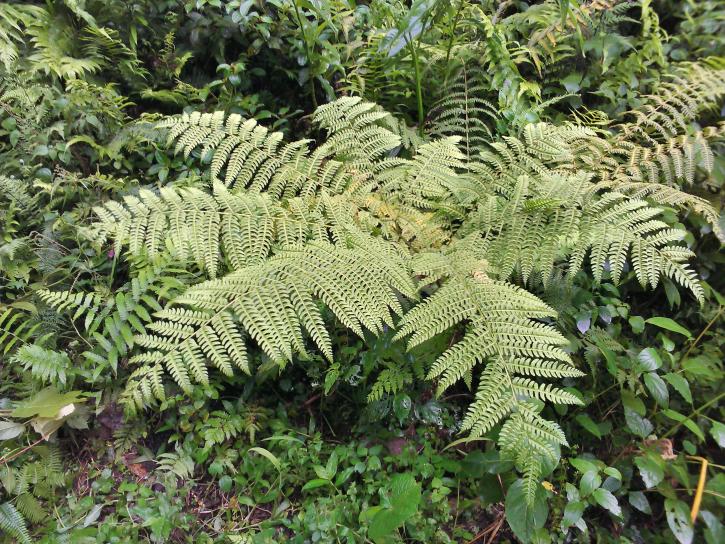
General aspect of the frond

**Figure 18b.**
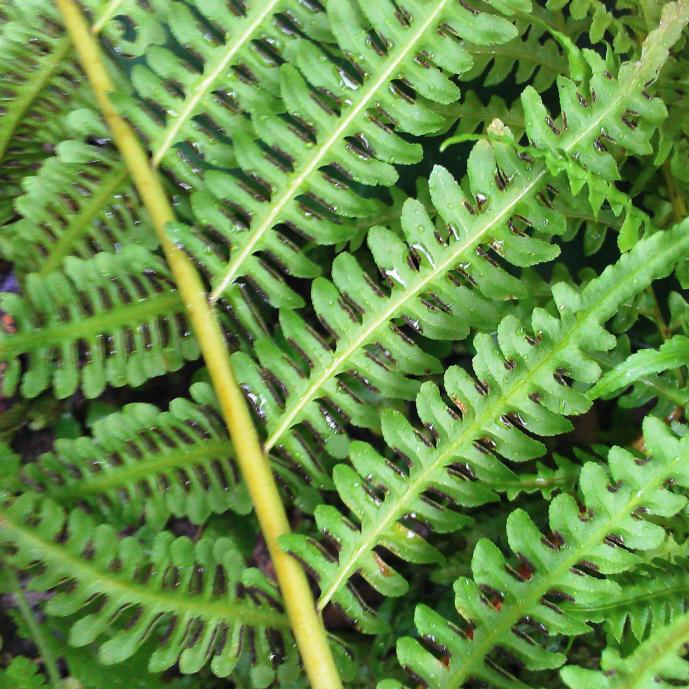
Lower face of the lamina showing sori

**Table 1.** List of descriptors with their discriminating power. The index value is the ratio between the number of couples with no common values and the total number of couples. For example, the value of the descriptor "habitat" index is 0.12 corresponding to 16 couples with no common values and a total number of 136 available couples.

**Descriptors**	**XPER**
1. type of habitat	16/136 (0.12)
2. type of terrestrial habitat	21/120 (0.18)
3. biotope	30/136 (0.22)
4. fronds dimorphism	52/136 (0.38)
5. type of dimorphism	0/6 (0.0)
6. frond length	39/136 (0.29)
7. frond width	85/136 (0.63)
8. fronds insertion on the rhizome	60/136 (0.44)
9. limb general shape	102/136 (0.75)
10. limb length	56/136 (0.41)
11. limb width	82/136 (0.6)
12. limb length/width ratio	83/136 (0.61)
13. limb texture	72/136 (0.53)
14. number of limb divisions	90/136 (0.66)
15. pinnae length	38/105 (0.36)
16. pinnae width	29/105 (0.28)
17. pinnae length/width ratio	44/105 (0.42)
18. pinnae dimorphism	36/105 (0.34)
19. position of the dimorphism	28/66 (0.42)
20. basal pinnae basiscopical development	0/10 (0.0)
21. terminal pinnae shape	7/28 (0.25)
22. pinnae general shape	38/105 (0.36)
23. pinnae base shape	76/105 (0.72)
24. pinnule general shape	24/45 (0.53)
25. pinnae insertion on the rachis	54/105 (0.51)
26. pinnae stipe length	16/28 (0.57)
27. winged frond	56/105 (0.53)
28. frond 3-branched	10/21 (0.48)
29. type of veins organization	96/136 (0.71)
30. type of dichotomy	5/15 (0.33)
31. veins organization	4/15 (0.27)
32. limb margin	83/136 (0.61)
33. stipe color	84/136 (0.62)
34. stipe length	55/136 (0.4)
35. rhizome size	42/136 (0.31)
36. rhizome habit	36/136 (0.26)
37. roots insertion on the rhizome	0/136 (0.0)
38. frond type	0/136 (0.0)
39. sporangia organization	30/136 (0.22)
40. sori position	14/105 (0.13)
41. sori position in relation to veins	0/0 (0.0)
42. sori position in relation to lamina	33/91 (0.36)
43. sori shape	54/105 (0.51)
44. indusium	86/136 (0.63)
45. indusium opening	0/1 (0.0)
46. indusium shape	0/1 (0.0)
47. indusium texture	0/1 (0.0)
48. covering	30/136 (0.22)
49. position of covering	6/105 (0.06)
50. rhizome covering	0/91 (0.0)
51. rhizome scale’s shape	48/91 (0.53)
52. rhizome scale’s color	32/91 (0.35)
53. stipe covering	15/36 (0.42)
54. stipe scale density	12/21 (0.57)
55. stipe muricule size	0/0 (0.0)
56. stipe hairs density	0/0 (0.0)
57. length of stipe hairs	0/0 (0.0)
58. limb covering	0/6 (0.0)
59. type of covering	6/6 (1.0)
60. limb muricule size	0/0 (0.0)
61. farinose coat color	0/0 (0.0)
62. limb hairs density	0/0 (0.0)

**Table 2.** Current nomenclature of the Pteridaceae species from Togo

**RIHA data**		**Current data**	
**Family**	**Species name**	**Family**	**Species name**
Pteridaceae	*Acrostichum aureum* L.	Pteridaceae	*Acrostichum aureum* L.
Adiantaceae	*Actiniopteris radiata* (Sw.) Link.	Pteridaceae	*Actiniopteris radiata* (Sw.) Link.
Adiantaceae	*Adiantum incisum* Forssk.	Pteridaceae	*Adiantum incisum* Forssk.
Adiantaceae	*Adiantum philippense* L.	Pteridaceae	*Adiantum lunulatum* Burm.
Adiantaceae	*Adiantum schweinfurthii* Kuhn.	Pteridaceae	*Adiantum schweinfurthii* Kuhn.
Adiantaceae	*Adiantum vogelii* Mett. ex Keyserl.	Pteridaceae	*Adiantum vogelii* Mett.ex Keyserl.
Adiantaceae	*Ceratopteris cornuta* (Beauv) Lepr.	Pteridaceae	*Ceratopteris thalictroides* (L) Brongn.
Adiantaceae	Doryopteris concolor var kirkii (Hook.) Alston.	Pteridaceae	*Doryopteris kirkii* (Hook.) Alston.
Adiantaceae	Doryopteris concolor var nicklesii (Tard.) Schelpe	Pteridaceae	Doryopteris concolor var nicklesii (Tard.) Schelpe
Adiantaceae	*Pellaea doniana* J.Sm. ex Hook.	Pteridaceae	*Pellaea dura* (Willd.) Hook.
Adiantaceae	*Pellaea doniniana* Link	-	-
Adiantaceae	Pityrogramma calomelanos (L.) Link. var. calomelanos	Pteridaceae	Pityrogramma calomelanos (L.) Link. var. calomelanos
Adiantaceae	*Nephrolepis bisserata* (Sw.) Schott.	Nephrolepidaceae	*Nephrolepis bisserata* (Sw.) Schott.
Adiantaceae	*Nephrolepis undulata* (Afzel. ex Sw.) J.Sm.	Nephrolepidaceae	*Nephrolepis undulata* (Afzel. ex Sw) J.Sm.
Vittariaceae	Vittaria guineensis (Desv.) var camerooniana	Pteridaceae	Haplopteris guineensis (Desv.) Crane var guineensis
Pteridaceae	*Pteris atrovirens* Wild.	Pteridaceae	*Pteris atrovirens* Wild.
Pteridaceae	*Pteris burtonii* Bak.	Pteridaceae	*Pteris burtonii* Bak.
Pteridaceae	*Pteris marginata* Bory.	Pteridaceae	*Pteris tripartita* Sw.
Pteridaceae	*Pteris togoensis* Hieron.	Pteridaceae	*Pteris togoensis* Hieron.
-	-	Pteridaceae	*Pteris similis* Kuhn.
